# Exploring Methods to Mitigate Fraud in Web-Based Surveys: Multicase Study Analysis

**DOI:** 10.2196/78671

**Published:** 2025-12-01

**Authors:** Madeleine Ennis, Regina-Maria Renner, Claudia Morando-Stokoe, Sharon James, Patricia A Janssen, Sara Leckie, Sheila Dunn, Danielle Mazza, Wendy V Norman

**Affiliations:** 1 Department of Obstetrics and Gynaecology Faculty of Medicine University of British Columbia Vancouver, BC Canada; 2 SPHERE Centre of Research Excellence in Women's Sexual and Reproductive Health in Primary Care, Department of General Practice, School of Public Health and Preventive Medicine Monash University Melbourne Australia; 3 School of Population and Public Health University of British Columbia Vancouver, BC Canada; 4 Department of Family and Community Medicine University of Toronto Toronto, ON Canada; 5 Women's College Research Institute Toronto, ON Canada; 6 Department of Family Practice Faculty of Medicine University of British Columbia Vancouver, BC Canada

**Keywords:** abortion, data collection, fraud, long-acting reversible contraception, methods, pregnancy, reimbursement incentive, surveys and questionnaires, women’s health

## Abstract

**Background:**

Web-based surveys are a cost-effective technique to engage a large population of participants in research projects, including those who were previously difficult to reach due to geographic location, safety, and vulnerability. While web-based surveys have many advantages, they can be more susceptible to fraud, especially when a generic invitation link or a financial incentive is offered. There is a paucity of literature presenting experiences for mitigating this type of fraudulent study response, yet this important foundation is needed to inform the work of researchers and institutional review boards (IRBs) to support the collection of high-quality, appropriate data.

**Objective:**

This study aims to analyze, compare, and contrast the range of strategies used to prevent, detect, and remove fraudulent responses by investigating 4 web-based surveys in Australia and Canada, each of which experienced fraudulent responses.

**Methods:**

Our descriptive multiple case study presents 4 research projects from Australia and Canada that experienced survey fraud. These web-based surveys recruited patients of, or clinicians providing, family planning services. We describe each study’s approach to preventing fraud (primary prevention; eg, CAPTCHA) and a screening protocol to detect fraudulent responses during data collection (secondary prevention). Once fraud was detected, each study team developed strategies to protect data integrity, in consultation with coinvestigators, ethics committees/ IRBs, and biostatisticians, to remove fraudulent respondents from the dataset (tertiary prevention).

**Results:**

All studies recruited via a generic survey link and provided remuneration, which are common risk factors for fraud. Several studies also relied on social media for recruitment. All 4 studies implemented tertiary fraud detection strategies to identify and remove fraudulent responses and maintain data integrity (removing between 16% and 45% of respondents). Including personal identifiers during data collection provided 3 of the studies with a more robust option to identify and remove fraudulent respondents. Where personal identifiers could not be used (eg, to protect the identity of a vulnerable study population), investigators relied on a complex fraud detection algorithm verified by manual team review.

**Conclusions:**

Commonly used web-based anonymized survey methods, particularly those offering incentives for participation, are at substantial risk for fraud. Across these 4 studies, robust fraud detection methods were essential to ensure data reliability, with varying strategies, such as using personal identifiers, applied based on specific survey contexts. Fraud mitigation criteria explored in this multicase analysis can be adapted to other web-based surveys, survey topics, and populations. Implementing the fraud prevention and detection methods within survey design will assist researchers and IRBs in protecting data integrity.

**Trial Registration:**

Australian New Zealand Clinical Trials Registry ACTRN12622000655741; https://www.anzctr.org.au/Trial/Registration/TrialReview.aspx?id=383919 and ClinicalTrials.gov NCT05793944; https://clinicaltrials.gov/study/NCT05793944

## Introduction

Increasingly, as society adapts digital technologies, academic research has moved from in-person, mail, fax, and phone surveys to web-based data collection [1-4]. Benefits of web-based surveys include cost-effective recruitment over a wide geographic area and access to unknown or equity-deserving populations that have previously been difficult to reach [3,5-8]. The internet facilitates anonymous research with fewer barriers for respondents who may take the survey at their convenience [3,5-7].

However, web-based surveys, especially when anonymous and using a generic link for recruitment, increase the risk of fraudulent responses that threaten data integrity [3,6]. Offering financial incentives as well as conducting research on socially or politically sensitive or stigmatized topics are additional risk factors for fraud [3,9]. Fraudulent web-based survey responses can be the result of human and computer-programmed (ie, bot) activity [3]. These can be broadly categorized as (1) those who misrepresent themselves in order to suit the survey’s eligibility criteria, (2) eligible respondents who duplicate their responses to receive additional financial incentive, or (3) those with malicious intent to invalidate the survey results [3].

Researchers have developed approaches and algorithms to decrease the risk for survey fraud with careful choices of their study design, and monitoring incoming survey records for responses that suggest fraud [3,6]. Nonetheless, preventing, identifying, and managing fraudulent responses still pose major challenges for both investigators and ethics committees or institutional review boards (IRBs) [10]. The increase in web-based surveys and associated fraudulent activity has necessitated research on how to reduce the risk of fraud when using anonymized surveys, particularly when financial incentives are offered [8,11-13]. However, little has been written about survey fraud mitigation, with a particular lack of papers that discuss detection strategies and their sensitivity or specificity, nor those that provide recommendations on how to diminish the impact of fraudulent responses on the validity of survey results.

To address this gap, we analyze, compare, and contrast a range of strategies used to prevent, detect, and remove fraudulent responses in 4 web-based surveys in Australia and Canada. Our analysis and implications may assist research teams and IRBs/ethics committees planning web-based surveys by highlighting important tools to protect data integrity as well as respondents’ identities.

## Methods

### Overview

We present a multicase study approach [14] with 4 web-based survey studies included in our descriptive analysis. We documented the study aims, design, setting, population, and data collection dates in Table 1. Each author was a coinvestigator in at least one of the presented case studies. For our analysis, we defined 3 stages of fraud prevention analogous to primary, secondary, and tertiary disease prevention [15]. Primary fraud prevention included survey and recruitment design to minimize the risk of experiencing fraud. Secondary prevention consisted of monitoring incoming responses for early fraud detection. We describe the study designs, recruitment, and primary and secondary prevention strategies in the Methods section. In Table S1 in Multimedia Appendix 1, we map detailed prevention strategies to apply during the stages of study development, recruitment, data cleaning, and analysis. Some of the strategies functioned at multiple levels of primary, secondary, and tertiary prevention. Once studies detected fraud, each developed tertiary prevention approaches to minimize the impact of fraud on the final results by removing fraudulent respondents. This required the development of fraud removal strategies and algorithms, as described in the Results section. Each case study team worked with their local IRB/ethics committee upon detecting fraud in their sample, with approval to conduct the analysis to identify and remove fraudulent respondents as part of their data cleaning. We followed the CHERRIES (Checklist for Reporting Results of Internet E-Surveys) reporting checklist (Table S2 in Multimedia Appendix 1). Each survey is available in Multimedia Appendix 2.

**Table 1:** Characteristics of the 4 case studies of web-based surveys that experienced fraud.

	Study aim	Study design	Setting and population	Timeframe
Case Study 1: CAPS2019^a^	To describe demographics of abortion providers, clinical care characteristics of abortion care services, and provider experiences with stigma and harassment.	Self-administered, cross-sectional, anonymized, bilingual (French and English), web-based survey	Pan-Canadian study including physicians, nurse practitioners, and administrators who provided abortion care in 2019.	Recruitment between July and December 2020.
Case Study 2: AusCAPPS^b^	To examine the knowledge, attitudes, and practices of general practitioners, general practice nurses, and community pharmacists about medication abortion in Australia.	Cross-sectional web-based survey	Australian general practitioners, general practice nurses, and community pharmacists.	Data collection between July and December 2021.
Case Study 3: EXTEND-PREFER	Primary aim: To assess whether a web-based educational video, describing all available contraceptive methods, developed for and with stakeholders and young Australian women living in rural and remote areas and for women from CALDc backgrounds speaking one of 4 languages (Mandarin, Cantonese, Hindi or Arabic), would increase their contraceptive knowledge, preference for, and uptake of LARCd. Secondary aims: To examine participant-reported likelihood of changing contraceptive use, including the use of LARC, after intervention; and to examine the cost and effectiveness of using social media to increase access to reliable contraceptive information.	Single group pre-post intervention design in which a web-based self-report questionnaire was completed at 3 timepoints	Australia based study including women aged 16-25 years, living in rural or remote Australia or from a CALD background who were not currently pregnant, did not wish to become pregnant within a year of the study taking place, had been sexually active with a male partner within the last 6 months or anticipated near-future sexual activity with a male partner and have not undergone any permanent form of contraception (including tubal ligation, hysterectomy or partner vasectomy).	Recruitment between February and April 2023.
Case Study 4: SmartMom RCT^e^	To evaluate the effectiveness of the SmartMom prenatal education texting program on knowledge of healthy pregnancy and childbirth, adoption of healthy behaviors, and maternal, fetal, and newborn health outcomes.	Two-arm, single-blinded (data analysis) RCT	Pan-Canadian study of healthy pregnant people.	Recruitment began in November 2023 and is to be completed in 2026.

^a^CAPS2019: The 2019 Canadian Abortion Provider Survey.

^b^AusCAPPS: Australian Contraception and Abortion Primary Care Practitioner Support Network.

^c^CALD: culturally and linguistically diverse.

^d^LARC: long-acting reversible contraception.

^e^RCT: randomized controlled trial.

### Case Study 1: The 2019 Canadian Abortion Provider Survey

#### Survey Design

Between July and December 2020, this study aimed to document the Canadian abortion care workforce characteristics and distribution, clinical care characteristics, and experiences with stigma and harassment via a survey of physicians, nurse practitioners, and abortion clinic administrators who provided abortion care in 2019 [16]. The Canadian Abortion Provider Survey was available in French and English on a Research Electronic Data Capture (REDCap) platform [17] and included sections on: demographics, clinical care characteristics of abortion care services, care of diverse populations, provider experience with stigma and harassment, and remuneration/contact for study results or future research (Multimedia Appendix 2). Complex skip pattern logic allowed respondents to see only relevant questions based on their prior answers. The survey took 30 to 80 minutes, depending on the range of abortion services respondents provided. As per the IRB’s request, the study team maximized protection of respondents’ identities and collected anonymized data. This study did not collect the IP addresses of respondents.

#### Recruitment

In Canada, abortion providers could include obstetrician/gynecologists, family physicians, and nurse practitioners. The identities and numbers of these individuals within their larger groups are unknown. The researchers partnered with various health care professional organizations, such as the Society of Obstetricians and Gynaecologists of Canada and the College of Family Physicians of Canada, to maximize recruitment of all eligible participants in the target populations. The partnering organizations distributed a generic survey link to their membership via email and newsletters. Additionally, we recruited via relevant institutions such as hospital departments of OB-GYN and family medicine, abortion clinics, and our web-based community of abortion practice platform [18]. The survey invitation listed the inclusion criteria. Initial mandatory survey questions verified respondents’ eligibility, and some questions required open-ended typed responses. Upon survey completion, respondents were directed to a separate elective survey, in which they could include contact details for remuneration, future research, and study results. This was the only survey section that asked respondents to provide a personal identifier in the form of an email. The researchers did not link the data from the 2 surveys in order to maintain the anonymity of respondents.

#### Ethical Considerations

This study received ethics approval (H18-03313) from BC Children’s and Women’s Research Ethics Board (REB) for both original data collection and subsequent fraud detection analysis. Participants reviewed and completed an anonymous consent statement through REDCap prior to the start of the survey. Respondents were offered a CAD $50 (equal to US $37.30) gift certificate as financial compensation, as detailed in the consent form, but not advertised in recruitment materials. All surveys were conducted and data stored using REDCap, hosted by BC Children’s Hospital Research Institute, aligned with Canadian privacy regulations.

#### Primary Fraud Prevention Strategies

This case implemented several primary fraud prevention strategies. The researchers recruited through professional organizations and university medical departments rather than via social media. Recruitment material did not indicate remuneration. Respondents could not request remuneration until they had completed all survey sections relevant to their designation. The beginning of the survey included a CAPTCHA to detect “bots.” The initial survey question asked respondents to confirm that they had not taken the survey before.

#### Secondary Fraud Prevention and Initial Fraud Detection

The research team monitored incoming responses every other week. Based on the literature, this consisted of confirming sensical combinations between select questions in the demographics [19]. The team identified suspicious patterns (answer combinations not possible within a regulatory or medical context) starting October 6, 2020, and determined that a more rigorous approach was required to identify and remove all fraudulent data prior to analysis.

The researchers identified the approaches of Ballard et al [3] and Teitcher et al [6], an evaluation and review of fraud detection techniques in web-based surveys, as the most applicable for informing the development of their tertiary fraud detection methods. They used a variety of criteria, such as questionnaire/instrument level data, software for detecting bots such as CAPTCHA, tracking respondents’ nonquestionnaire data, including personal information such as email addresses, and study design level decisions, such as consent and compensation.

### Case Study 2: The Knowledge, Attitudes and Practices of Australian Primary Care Clinicians in the Provision of Long-Acting Reversible Contraception and Medication Abortion

#### Survey Design

Launched on July 19, 2021, three national web-based surveys aimed to describe the knowledge, attitudes, and practices of Australian general practitioners, practice nurses, and community pharmacists regarding long-acting reversible contraception (LARC) and medication abortion. Based on previous LARC and medication abortion surveys we conducted this as part of the Australian Contraception and Abortion Primary Care Practitioner Support Network study (ACTRN12622000655741) [20]. An ethics committee approved respondent explanatory statement was embedded on the survey landing page that gave details about the research purpose, inclusion criteria (eg, Australian Health Practitioner Registration), and investigator team. From piloting, the survey took an estimated 10 to 15 minutes to complete and consisted of a maximum of 50 questions over 9 pages, written in English language. Questions could be revisited by respondents prior to completion. Questions were open and closed in design and included demographics about age, gender, postcode, and number of years they had been practicing as a clinician. The survey used timestamps and complex branching logic. Answer options included Likert scales and free-text (eg, “other” responses).

#### Recruitment

Using a generic survey link, the research team used convenience sampling methods, such as through research team contacts, a purchased professional address list from the Australasian Medical Publishing Company and trial partners from industry and professional organization newsletters relating to the 3 clinician groups (eg, Royal Australian College of General Practitioners, Australian Primary Health Care Nurses Association, Pharmaceutical Society of Australia). The social media platforms used for recruitment included Facebook, X, and LinkedIn; tagging these clinician groups and women’s health organizations (eg, Sexual Health Victoria, Family Planning New South Wales). Recruitment concluded 6 months following launch, once enrolment reached the desired sample target of approximately 500 verified responses for each professional group.

#### Ethical Considerations

The Monash University Human Research Ethics Committee approved the Australian Contraception and Abortion Primary Care Practitioner Support Network knowledge, attitudes, and practices (#28002) survey documents as well as any changes to respondent verification to improve the fraud detection process. Participation was voluntary, and consent was implied by survey completion. Recruiting materials advertised an Aus $40 (equal to US $30.05) e-gift card incentive and the time required to complete the surveys. All surveys were conducted using REDCap, hosted and managed by Helix (Monash University) [21,22]. Once collected, the data were exported to Microsoft Excel for cleaning and saved in line with Monash University data storage requirements.

#### Primary Fraud Prevention Strategies

Given the reimbursement offer for survey completion, response inclusion was first determined through manual matching of the respondent’s name and postcode to their listing on the publicly available Australian Health Practitioner Registration Agency website. A combination of human and computer-based strategies were then applied, including: duplicate removal, ensuring only those who met the inclusion criteria could complete the survey (eg, Australian Health Practitioner Registration, used Australian postcodes and worked in general practice as a primary or secondary place of employment), a minimum set time of 5 minutes for survey completion (determined at piloting), timestamp review, review for nonsensical email addresses (eg, mostly numbers or no relationship with the respondent’s name) and responses (eg, phrases given instead of a name), and respondent behaviors such as survey completion in “batches.”

#### Secondary Fraud Prevention and Initial Fraud Detection

The researchers recorded an influx of general practitioner and practice nurse surveys in which respondent names did not match email addresses over a period of 4 days. The practice nurse survey received the majority of unverified responses over the course of survey recruitment. The fraudulent survey activity was detected approximately 2 weeks after launch. At this time, the team learned that the surveys had been advertised on private Facebook groups and through one organizational newsletter. The researchers then adopted a more rigorous approach by trying to match recruitment methods (eg, the identification of particular social media posts) to legitimate or fraudulent survey response activity.

### Case Study 3: The EXTEND-PREFER Project

#### Study Design

The EXTEND-PREFER project aimed to assess whether a co-designed web-based educational video could increase contraceptive knowledge, preference for, and uptake of LARC in young women from priority populations [23]. From February 2023 to April 2023, web-based advertising recruited young culturally and linguistically diverse (CALD) women and women living in rural and remote areas aged 16-25 years. Respondents completed a prevideo survey (S1; 60 questions across 5 pages); watched a 13-minute co-designed video, and completed a follow-up survey (S2; 35 questions across 3 pages) immediately after; and another survey (S3; 40 questions across 3 pages) 6 months later. The prevideo survey (S1) collected information relating to demographic characteristics and provided a baseline for contraceptive knowledge, preference for, and uptake of LARC (using a Likert scale). The prevideo survey (S1) also collected personal identifiers, including participants’ first name, last name, email address, phone number, postcode, and IP address for verification purposes and so that they could be contacted to complete the follow-up survey (S2). The follow-up survey (S2) addressed respondents’ contraceptive knowledge and preference after watching a 13-minute co-designed video detailing all contraceptive methods available in Australia. The 6-month follow-up survey (S3) collected data on contraceptive methods currently used to assess whether the video changed participants’ preference for, knowledge of, or uptake of LARC. All surveys were completed using Qualtrics (Qualtrics International Inc) [23], and the data were exported to Microsoft Excel for cleaning and analysis. After completion of S1 and S2, respondents were offered an Aus $20 (equal to US $15.025) e-gift card for their time. Respondents who completed S3 were offered an additional Aus $20 (equal to US $15.025) e-gift card resulting in a maximum of Aus $40 (equal to US $30.05) for participants’ time.

#### Recruitment

The project used posts on LinkedIn and X and paid advertising on Facebook and Instagram to recruit participants. In order to appropriately target respondents, the research team developed 5 sets of advertising assets, which included one ad set targeted at rural communities and 4 ad sets targeted at CALD communities speaking Arabic, Cantonese Mandarin, or Hindi. The ad assets were in English, Arabic, Cantonese, Mandarin, and Hindi and each ad asset had a generic survey link for the language being used.

#### Ethical Considerations

This study received ethical approval (#24907) from the Monash University Human Research Ethics Committee for data collection and analysis. Consent was implied by survey completion. Respondents were provided a maximum of Aus $40 (equal to US $30.05) (Aus $20 for completing S1 and S2, Aus $20 for S3 [Aus $20 equal to US $15.025]). Survey incentives were advertised on recruitment materials. All surveys were conducted and data stored using Qualtrics, hosted and managed by Monash University.

#### Primary Fraud Prevention Strategies

In order to take part in the study, prospective respondents were required to complete an initial eligibility survey (first half of S1) that included questions about the respondents’ age, relationship status, pregnancy status, pregnancy intention, ethnicity, rurality and sexual activity during the 6-month period prior to commencing the survey. Respondents were unable to proceed if they failed to meet the study inclusion criteria based on their responses.

#### Secondary Fraud Prevention and Initial Fraud Detection

The research team detected fraudulent activity approximately 2 weeks after the survey recruitment launch when a researcher noted duplicate entries (ie, responses with the same identifiers and responses with identical answers) while reviewing the survey data to arrange respondent remuneration. The team continued to implement planned primary fraud detection strategies and additionally began to conduct basic manual screening to check respondents who met the eligibility criteria if they were flagged by fraud detection features in Qualtrics (ie, ID Duplicate, Relevant ID Duplicate Score, Relevant ID Fraud Score, and ReCAPTCHA score).

### Case Study 4: SmartMom Randomized Controlled Trial

#### Study Design

Launched in November 2023, the SmartMom randomized controlled trial (ClinicalTrials.gov NCT05793944, UBC REB H22-00603) aimed to evaluate the effectiveness of the SmartMom prenatal education texting program [24]. The main goals were to determine if healthy pregnant people receiving the SmartMom program have improvements in (1) knowledge of healthy pregnancy, labor, and birth, (2) standardized measures of mental health, (3) adoption of positive health behaviors in pregnancy, and (4) maternal, fetal, and newborn health outcomes. Inclusion criteria included (1) the ability to read and understand English at a Grade 8 level; (2) living in Canada but outside of British Columbia and Nova Scotia (SmartMom was already offered in these provinces); (3) 15 weeks pregnant or less; (4) singleton pregnancy; (5) aged 15 years or older; (6) without prepregnancy conditions requiring medication including hypertension, cardiac disease, diabetes, mental health disorder, or neurological disorder; (7) having a cell phone with cellular and internet access; and (8) not having been previously enrolled in SmartMom. Participants randomized to the Treatment arm received the SmartMom texting program, which includes evidence-based text messages 3 times per week through their pregnancy. Control participants received general interest messages once per week with study updates and pregnancy topics that were not aimed at promoting behavior change. Participants were asked to provide their personal health number to link to third-party health authority databases, and complete 3 web-based surveys: after enrolment, in late pregnancy, and after birth. Surveys took about 15-20 minutes to complete in total. There is one question per screen, with multiple screens to complete the questionnaire. Participants could edit answers to previously answered questions. Completion rates for initial surveys were 90% and for final surveys 72%. Surveys are available in Multimedia Appendix 2. All surveys were conducted and data stored using REDCap, hosted by BC Children’s Hospital Research Institute, in a manner compliant with Canadian privacy regulations. PHNs were encrypted and sent through dedicated secure file transfer servers to provincial or national data registries for linking with personal health data. Personal health data will be sent to the researchers using an encrypted file without the PHN or any other identifying information.

#### Recruitment

To maximize geographic and demographic representation, the study was advertised via paid ads on Instagram, Facebook, and Reddit, targeting women aged 18-44 years in Canada. Ads mentioned that gift cards would be provided to participants and included a link to the study website. Individuals interested in the study could complete a web-based screening form via a generic link to a REDCap survey consisting of eligibility questions. If responses indicated the individual met eligibility criteria, they were automatically directed to a second survey to provide their phone number, province, due date, and whether they were older than 18 years. Study staff reviewed the screening information and then contacted the individuals by text initially and then via a phone call. Study staff described participation in the study, answered questions, and then provided a link to the digital e-consent form via text. Once the e-consent form was complete, we randomized the participant to a study arm. Participants received survey links by text for treatment or control arms. Recruitment for the trial is ongoing.

#### Ethical Considerations

This study received ethical approval (H22-00603) from BC Children’s and Women’s REB for both original data collection and subsequent fraud detection analysis. Participants signed a consent form prior to randomization. Respondents were offered a gift certificate for each of 3 surveys completed (CAD $1, CAD $20, and CAD $25; equal to US $0.74, US $14.82, and US $18.52, respectively) as financial compensation, as detailed in the consent form but not advertised in recruitment materials. All surveys were conducted and data stored using REDCap, hosted by BC Children’s Hospital Research Institute, aligned with Canadian privacy regulations.

#### Primary Fraud Prevention Strategies

Web-based screening and survey forms included CAPTCHA to minimize activity by bots. Study staff reviewed information provided on the screening form, and if information was inconsistent or nonsensical, it was reviewed with the individual for accuracy. If the individual did not, then provide information that was consistent (ie, due date with gestational age), study staff deemed them ineligible. Survey responses included the participant’s phone number, allowing the removal of duplicates. Cookies were not used, nor did we check the IP addresses of respondents’ computers.

#### Secondary Fraud Prevention and Initial Fraud Detection

The team first detected fraudulent activity following a large increase in web-based screening activity over one weekend, with the number of screens exceeding the number of ad clicks, a high number of screens indicating ineligibility, and numerous screening forms filled out just minutes apart. In addition, the researchers found multiple screens with the same contact phone number, an increase in screens with inconsistent information (ie, due date and gestational age not matching), and an increase in responses to an introductory text asking for communication via text without a phone call. Similar to case study 2, the team learned that the study information had recently been shared via web-based platforms (eg, Facebook groups) related to completing surveys for remuneration, and not restricted to the population relevant for study eligibility (ie, pregnant people). The researchers identified the need for a rigorous review of enrolled participants and for updated screening prior to enrolment.

In response, the team updated the initial web-based screening, with REB approval, to include phone number and due date in addition to the second screening form, to help detect multiple screening attempts by the same person. A detailed phone script included questions about how the pregnancy was going, whether the pregnancy had been confirmed, and what the due date was based on. If individuals provided inconsistent, nonsensical, vague, or minimal information, then the study staff requested documentation of their due date or gestational age, for example, a copy of their ultrasound report.

## Results

### Overview

These 4 cases of web-based surveys from Canada and Australia all used primary prevention strategies to prevent fraud, secondary prevention to detect suspicious activity for fraud, and once fraudulent responses were identified, developed tertiary prevention methods to identify and remove fraudulent data. All 4 studies implemented multiple primary and secondary fraud prevention techniques into the study design, such as the use of CAPTCHA questions and incorporation of a time stamp. While all 4 studies monitored incoming survey responses for fraud using secondary prevention techniques, as highlighted above, an increase in the number of incoming survey responses was the first detected sign of fraud in 2 of the 4 cases. Nonsensical or noncoherent responses and detection of duplicate responses alerted researchers to possible fraud in 3 of the 4 surveys. The researchers adapted several of the primary and secondary prevention strategies into tertiary strategies, as highlighted in Table S1 in Multimedia Appendix 1. In the following paragraphs and Table S1 in Multimedia Appendix 1 we describe the tertiary prevention strategies of each case study in more detail.

### Case Study 1: Tertiary Fraud Prevention and Identification and Removal of Fraudulent Responses

The 2019 Canadian Abortion Provider Survey: Following a literature review on fraud in web-based surveys, this team mapped applicable criteria as listed in Table S1 in Multimedia Appendix 1 to their survey in collaboration with the applicable IRB and coinvestigators. The team created and tested an algorithm aiming to protect valid responses and detect fraud, then allocated negative fraud points to protective criteria (eg, sensical free-text responses) components and positive fraud points to components considered suspicious (eg, nonsensical answer combinations such as being a maternal-fetal medicine subspecialist but registered with the College of Family Physicians of Canada), with a higher number of points in either direction the stronger the assumed predictive value. The team focused on the mandatory questions that all respondents had completed. Additionally, IRB permission was obtained to temporarily link the email addresses provided when requesting remuneration to the respondent’s answers. The algorithm was piloted, designating respondents with known or institution emails as negative controls, and those with highly suspicious pattern-based emails as positive controls to assess each fraud criterion. Sensitivity and specificity analyses were conducted, respectively, on each criterion component and are presented in Table S3 in Multimedia Appendix 1. Free text answers had the highest sensitivity (92.7%) and specificity (100%) while nonsensical answer combinations had lower sensitivity (54.5% combined with a high specificity (99.1). Where possible the algorithm criteria were programmed in R Statistical Software (R Foundation for Statistical Computing) and at least 2 independent researchers manually assigned fraud points to the remaining criteria. Disagreements were discussed among multiple team members until a consensus was reached. The pilot data was reviewed and the team reached consensus on the criteria to include in the final algorithm.

The final fraud detection algorithm (Figure S1 in Multimedia Appendix 1) included the assessment of responses or response combinations for being nonsensical or nonprobable, and of those protected by sensical free text (eg, providing explanations to response options where “other” answer option was selected). It included the survey submission date before or after we detected fraud and remuneration criteria, in which not asking for remuneration was protective. No single criterion was used to exclude or include respondents. Depending on the combination of scoring results from the different criteria, the team included (low fraud score; n=342), excluded (high fraud score; n=302), or marked a participant for further review (midrange fraud score; n=271). These cut points were determined using the results from the sensitivity/specificity analysis described above. Then, responses marked for further review were manually assessed for additional free-text protective criteria responses or further nonsensical/nonprobable criteria responses. This survey had 1050 respondents, and after removal of participants due to data cleaning (n=135; duplicates, etc) and fraud detection (415/915, 45.3%), 500 respondents were included for analysis.

### Case Study 2: Tertiary Fraud Prevention and Identification and Removal of Fraudulent Responses

The Knowledge, Attitudes and Practices of Australian Primary Care Clinicians in the Provision of Long-Acting Reversible Contraception and Medication Abortion: Within the first 2 weeks after survey launch, the general practitioner and practice nurse surveys recorded over 300 responses over 4 days, in which respondent names did not match email addresses and were completed in batches from the same email account. Following advice from the research team and Monash University’s information technology and REDCap staff, the team added a text-based CAPTCHA and a “honeypot” question not visible to human respondents to detect bots using the REDCap @HIDDEN-SURVEY function for improved primary fraud prevention. Changes were also made to the respondent explanatory statement, indicating full name verification would be requested via email prior to reimbursement. Following a reply email from the respondent, the team began to verify each respondent’s name alongside the practice postcode given in the survey with their details on the AHPRA website. Active recruitment resumed following ethics committee approval of these changes. The team also developed an internal protocol for respondent verification and reimbursement. Across 3 surveys, 5598 (100%) respondents started the survey. From these, 45.5% (n=2547) could not be verified or were discontinued. Other reasons for excluded responses included duplicates or if the respondent did not complete the mandatory questions or did not work in general practice. A total of 27.2% (n=1521) responses were included in the final analyses.

### Case Study 3: Tertiary Fraud Prevention and Identification and Removal of Fraudulent Responses

The EXTEND-PREFER Project: Throughout the recruitment phase, the research team continued to implement the initiated primary and secondary fraud prevention measures and additionally conducted rigorous manual screening. This included a researcher checking all new responses daily for duplicate entries and if entries were flagged by fraud detection features in Qualtrics, including “ID Duplicate,” “Relevant ID Duplicate Score,” “Relevant ID Fraud Score,” and “ReCAPTCHA score”). Team members decided to conduct a more rigorous manual screening than initially planned due to a sudden influx (over a 48 hour period) of more than 800 likely fraudulent responses roughly one month into the recruitment phase. During this screening process, the researchers identified and excluded any completed responses deemed likely to be fraudulent or a duplicate entry, based on personal information provided (eg, respondent’s name, email address, and postcodes), computer information available (eg, IP address and timestamp), and those with unrealistically short response times (eg, <8 minutes).

For the rural and remote surveys, 3614 (100%) respondents started the initial survey. From these, 8.3% (n=300) respondents did not meet the eligibility criteria or could not be verified and were therefore discontinued. For the CALD surveys, 2348 (100%) respondents started the initial survey. From these, 16.4% (n=385) of respondents did not meet eligibility criteria or could not be verified and were therefore discontinued. Responses were excluded if there were multiple entries with a duplicated name, mobile number, or email address (retaining first response, and excluding additional entries), if it took the participant less than 800 s (approximately 13 mins) to complete the survey or if there were multiple responses with the same IP address (retaining first response, and excluding additional entries).

### Case Study 4: Tertiary Fraud Prevention and Identification and Removal of Fraudulent Responses

SmartMom Randomized Controlled Trial: To identify fraud, the researchers manually reviewed enrolled participants and categorized likelihood of fraud based on suspicious activity including (1) more than one screening attempt; (2) failure to provide personal health number; (3) inconsistency of information provided across surveys and screening; (4) same response provided for all questions in multiple choice standard scale surveys; (5) notably high interest in gift cards, manifested through asking several questions; and (6) low engagement with the texting program (ie, not texting keywords to receive optional content or notify of baby’s birth). Multiple screening attempts, high interest in remuneration and inconsistent or nonsensical responses on screening were assessed by the team to be most consistent with fraudulent activity. Repeat questions in surveys over time were less indicative, as the team noted high inconsistency across their study. It appeared that poor recall, high error rates in responses, and lack of attention to detail were common in this perinatal study population. Based on the high fraud risk criteria above, each participant was categorized as low, medium, or high likelihood of being fraudulent. Data for participants with high fraud likelihood was excluded from analysis, and the team will undertake sensitivity analysis with and without those with medium fraud likelihood.

### Synthesis of Findings Across Case Studies

Across the 4 case studies, the researchers implemented primary fraud prevention strategies, but all surveys experienced fraud. We found that when primary and secondary fraud prevention techniques were more robust and included personal identifiers, researchers were able to save time and use less complicated tertiary fraud prevention techniques. Many fraud prevention and detection strategies, such as IP address monitoring and screening questions relevant to the inclusion criteria, can be used across prevention levels (primary, secondary, and tertiary) and have been deliberately included in the study design phase. Protecting participant identity by foregoing the collection of personal identifiers came at the cost of weakening fraud prevention strategies. Strategies to recruit an unidentifiable population, such as the use of a generic survey link and recruiting on social media, likely increased the risk of fraud by sharing the study with a wider audience than those who were eligible. Closely monitoring response patterns, while being a time-sensitive and time-consuming technique, proved critical in all 4 case studies in order to promptly identify the onset of suspicious and ultimately fraudulent respondent patterns. All 4 case studies offered remuneration for completing their surveys. Once fraud was detected, inclusion of checking questions that verify previous responses, mandatory free text questions, minimum time specification or timestamps, and inclusion of personal identifier fields were among the most commonly used and helpful tertiary prevention methods. Ultimately, 45.3% (n=415), 45.5% (n=2547), and 16.4% (n=385) of respondents were removed in case studies 1, 2, and 3, respectively. Case study 4 had not completed data analysis and, therefore, is unable to report fraud detection rates.

## Discussion

### Principal Findings

Our findings highlight that prevention and correct identification of fraud in web-based surveys are essential to maintain data integrity. Despite using primary prevention strategies to prevent fraud, all 4 research projects experienced substantial fraud, and needed to remove up to 45% of survey responses. Each study used secondary prevention methods that identified participants likely to be fraudulent. In consultation with members of our respective coinvestigator teams, ethics committees/IRBs, and, in some cases, biostatisticians, each study team developed tertiary prevention strategies to remove fraudulent respondents from the dataset and was able to move forward with recruitment and analysis.

### How to Prevent Fraud in Future Studies?

There is limited literature on survey fraud mitigation, with a particular gap for any papers that discuss detection and prevention methods. A further gap is evident for the paucity (or lack) of articles that provide recommendations on how to reduce the impact of experiencing fraud on the validity of survey results across studies with varying degrees of personal identifiers and web-based recruitment strategies. Our manuscript builds on previously described fraud removal approaches and showcases how these can be tailored to surveys with different recruitment methods and data collection, including degrees of personal identifiers. Our cases highlight the importance of carefully weighing the pros and cons of study design, survey instruments, and recruitment decisions, while balancing the individual study needs versus the risk of experiencing fraud. A proactive, multilayered approach to fraud prevention throughout the study design, recruitment, and data analysis is crucial.

While case studies 1 and 2 recruited clinicians, and case studies 3 and 4 recruited end users of health care, all encountered fraud, indicating the pervasiveness of fraudulent behavior. All studies recruited using a generic survey link to widely recruit a population of at least partially unknown individuals. This is known to be a higher risk for fraud compared with sending individual survey invitation links [25]. Recruitment on social media further compounded the fraud risk in studies 2-4, although using only professional networks did not sufficiently protect study 1 from fraudulent respondents either [26]. Given that each study offered remuneration for participation, monetary incentives appear to have been a motivator for fraudsters. This aligns with prior research suggesting that financial compensation can drive fraudulent behavior in survey-based studies [9,27,28]. Avoiding remuneration, not advertising remuneration, or choosing options such as a lottery has been found to be protective [29]. Fortunately, there are evolving mechanisms to identify or remove bots, including the use of CAPTCHA or Honeypot questions [30].

One significant risk for fraudulent participation and a serious obstacle to identifying fraudulent responses is exemplified by the first case study, in which the absence of personal identifiers during data collection made the identification of fraudulent respondents more complex and time-consuming. While this approach was designed with the IRB to protect participant anonymity when researching a sensitive study population, it potentially increased the risk of fraud and further hindered the research team’s ability to quickly and effectively remove fraudulent responses, and required the implementation of tertiary strategies. This contrasts with case studies 2 through 4, which were able to rely on well-documented methods to detect fraud, such as personal identifiers and IP addresses that may have been protective and required a less rigorous tertiary prevention protocol [6,28,31]. Contacting respondents to verify their information and eligibility criteria (as in case studies 2 and 4), has been used as an effective method to detect fraud [3].

These 4 case studies highlight the conflict between safeguarding participant privacy, especially when surveying vulnerable populations, and ensuring the accuracy and reliability of collected data. While the first case study, which did not use identifiers, effectively used tertiary prevention strategies, it increased the time commitment and complexity of detecting fraud. Similar to our first case study, Lawlor et al [28] described a framework to identify suspected web-based survey fraud, in which they emphasize the importance of using multiple criteria combined in an algorithm to carefully determine the threshold to exclude a respondent. Among the multiple criteria in the tertiary fraud detection and removal algorithm of the first case, the following were especially effective: multiple screening attempts; implausible or inconsistent responses for clinical information (though can also be a sign of survey fatigue); refusal to provide requested identifier; several questions about remuneration; and lack of interest or questions about the purpose and nature of the study. Some of these effective criteria, such as open-ended questions or embedding several questions that can be linked to expose nonsensical answer combinations, are simple to implement and should be considered at the survey development stage. Unusual email addresses with patterns that switched between numbers and letters and did not appear to include initials or a name have been found to be associated with fraudsters in other studies, and are consistent with several of our case studies [3,10,32,33].

### Strengths and Limitations

We presented 4 case-studies from 2 countries, which surveyed clinician as well as patient respondent populations, enhancing the relevance of the findings across different reproductive health topic areas, populations, and geographical contexts. We included large-scale national web-based surveys, providing valuable insights into the scalability and effectiveness of fraud prevention strategies. All case studies are recent and applicable to contemporary issues in fraud prevention. Additionally, the variation in study design between case studies enabled us to conduct a rich exploration of primary, secondary, and tertiary fraud prevention strategies, offering a nuanced description of how these approaches can be applied at different stages of study design and how they are interconnected.

The case studies are based on a convenience sample of studies conducted by teams that included one or more of our author group, and all focus on reproductive health, which limits the generalizability of the findings to other areas of public health. Furthermore, the tertiary prevention strategies presented are specifically tailored to each case study context and may require adaptation to be effective in other contexts. This limitation is mitigated to some extent by our presentation of broad components, offering a framework that can be adapted in different contexts. While all studies removed fraudulent responses, as they were unable to definitively determine which participants were fraudsters, it is possible they excluded some valid respondents.

### Conclusions

Web-based surveys are at substantial risk for fraudulent responses, and prevention strategies are essential to enable researchers and IRBs to protect data integrity. Ideally, strategies should be incorporated into the survey and recruitment during the study design phase. Developing strong primary fraud prevention strategies, paired with having a predefined approach to monitoring for fraud (secondary prevention), can reduce the reliance on tertiary methods to remove fraudulent responses and ensure data integrity. The criteria we have presented in the fraud detection algorithm can be adapted to other web-based surveys, survey topics, and populations.

## Appendix

Multimedia Appendix 1 Additional tables.

Multimedia Appendix 2 The surveys for case studies 1-4.

## Abbreviations

CALD: culturally and linguistically diverse
CHERRIES: Checklist for Reporting Results of Internet E-Surveys
IRB: institutional review board
LARC: long-acting reversible contraception
REB: Research Ethics Board
REDCap: Research Electronic Data Capture

## Data Availability

The datasets generated or analyzed in these studies have varying availability based on ethical and funding approvals. For the 2019 Canadian Abortion Provider Survey (case study 1) and the Australian Contraception and Abortion Primary Care Practitioner Support Network knowledge, attitudes, and practices (case study 2), the datasets are not publicly available due to a lack of approval from the respective research ethics boards. Similarly, for EXTEND-PREFER (case study 3), the datasets cannot be shared publicly without approval from the funder or the research ethics board. In contrast, the datasets from The SmartMom randomized controlled trial (case study 4) are available from the corresponding author upon reasonable request.
