# Supplementary material for: Exploring Methods to Mitigate Fraud in Web-Based Surveys: Multicase Study Analysis
Source: J Med Internet Res. 2025 Dec 1;27:e78671. doi: 10.2196/78671 (PMC12706441; doi:10.2196/78671)

Table S1. Use of primary, secondary and tertiary fraud prevention strategies in case studies of web-based surveys.

Category	Fraud Prevention Strategies	Functionality/benefit	Considerations	CAPS 2019 (Case Study 1)	Aus CAPPs (Case study 2)	EXTEND-PREFER (Case study 3)	SmartMom RCT (Case study 4)
**Study development**							
	Insertion of CAPTCHA question[1]	Widely used mechanism for testing a human versus bot response	Human responses may score below the ReCAPTCHA threshold<br><br>Slows survey completion time	Yes	Yes[4]	Yes	Yes
	Two-factor authentication[1,2,3]	Adds additional layer of security to the authentication process	Time consuming<br><br>Requires personal identifiers	No	No	No	No
	IP address monitoring for duplicate response and ensuring target audience is reached.[1,2,3]	Eliminates duplicates<br><br>For location-based target audiences this method allows researchers to confirm if respondents are from target audience.	Time consuming<br><br>Respondents may be from target audience but completing the survey at the same location<br><br>Does not completely mitigate for bot activity	No	No	Yes	No
	Incorporation of a 'honeypot'	Only visible to a bot	May be detected and	No	Yes[4]	No	No

	(hidden) question[1]		bypassed by some bots				
	Incorporation of timestamp[2,3]	Flags dates and times, bundles of participants beginning and completing the survey at the same time	Time of day/night survey completion is not a reason on its own to exclude a respondent	Yes	Yes	Yes	Yes
	Incorporation of minimum time specification for survey completion[2,3]	Eliminates respondents who completed the survey impossibly fast	Bots can imitate human response times	No	Yes	Yes	No
	Add screening question/s relevant to the inclusion criteria[1,2,3]	Confirms respondent eligibility	Does not completely mitigate for bot activity<br>Slows survey completion time	Yes	Yes	Yes	Yes
	Include a “Where did you hear about this survey?” style question[2,3]	Opportunity to tailor recruitment strategies aligned with valid respondents	Slows survey completion time<br>Does not completely mitigate for bot activity	No	Yes	No	Yes
	Additional demographic questions e.g. clinician registration name/type/ number, state, post/zip code[2,3]	Assists with verification processes<br>Adds demographic data for analysis	Slows survey completion time	Yes	Yes	Yes	Yes

	Collect personal identifiers[1,2,3]	Allows you to verify respondents' identities, and when detecting fraud, use suspicious email addresses, names, usernames, passwords, or phone numbers.<br><br>May not be approved by REBs	Decreases participant's anonymity<br><br>Respondent may construe this as an invasion of privacy<br><br>Slows survey completion time	No	No	Yes	Yes
	Include email address or phone number field for verification[1,2,3]	Allows a mechanism for research team contact<br><br>Discourages the creation of easily obtainable email addresses<br><br>Allows for easier detection of nonsensical email addresses	Slows survey completion time<br><br>Respondent may construe this as an invasion of privacy<br><br>IRBs may not approve	No	Yes	Yes	Yes
	Inclusion of a mandatory (cannot be bypassed) free text question[1,2,3]	Allows monitoring of nonsensical responses<br><br>Additional mechanism for data collection	Does not completely mitigate for bot activity	No	Yes	No	No
	Inclusion of question combination s that allows	Allows checking for data consistency	Risk of respondent error	No	Yes	No	Yes

	verification of sensical answer between multiple questions[11] [2,3]	within the same survey	Does not completely mitigate for bot activity				
**Recruitment**							
	Targeted recruitment e.g. organizational newsletters[1]	Limits recruitment to the desired sample	Does not mitigate for bot activity<br>Potential for:<br>Self-selection bias<br>Fraudulent responses still possible within the desired sample<br>May increase recruitment costs	Yes	Yes	No	No
	Recruitment via social media[1]	Recruitment materials reach a large audience at lower cost	Increases risk of recruiting outside target sample, including fraudsters	No	Yes	Yes	Yes
	Use of generic link[1]	Reach participants when they are not identifiable (e.g. you do not know who your sample is)	Increases risk of fraudsters accessing your survey	Yes	Yes	Yes	Yes
**Data collection**							

	Routine review of respondent patterns[2]	Track sources of valid responses<br><br>Opportunity to boost/change recruitment approaches<br><br>Opportunity to manually review for non-sensical response combinations	Time consuming	Yes	Yes	Yes	Yes
**Incentives**	Provide remuneration[1,2,3]	Provides financial incentive for legitimate participants	Provides financial incentive that motivates fraudulent respondents	Yes	Yes	Yes	Yes
	Advertising remuneration[1]	Increases interest from people who are motivated by remuneration	Attracts interested from fraudulent responses	No	Yes	Yes	Yes
	Use a lottery system[1]	Removes guaranteed financial incentive that motivates fraudulent responses	Removes guaranteed financial incentive for legitimate participants	No	No	No	No

[1] Primary fraud prevention

[2] Secondary fraud prevention

[3] Tertiary fraud prevention

[4] Added to the survey after fraud was initially detected

Table S2. Checklist for Reporting Results of Internet E-Surveys (CHERRIES)

Checklist Item	***Explanation***	***Case Study 1: Page Number***	***Case Study 2: Page Number***	***Case Study 3: Page Number***	***Case Study 4: Page Number***

Describe survey design	Describe target population, sample frame. Is the sample a convenience sample? (In "open" surveys this is most likely.)	Page 4-5	Page 6	Page 8	Page 9
IRB approval	Mention whether the study has been approved by an IRB.	Page 10	Page 10	Page 10	Page 9
Informed consent	Describe the informed consent process. Where were the participants told the length of time of the survey, which data were stored and where and for how long, who the investigator was, and the purpose of the study?	Page 4	Pages 6-7	Page 8	Page 9
Data protection	If any personal information was collected or stored, describe what mechanisms were used to protect unauthorized access.	Page 4	Pages 7, 13	Page 8	Page 9
Development and testing	State how the survey was developed, including whether the usability and technical functionality of the electronic questionnaire had been tested before fielding the questionnaire.	Page 4-6	Page 6	Page 8	Page 9
Open survey versus closed survey	An "open survey" is a survey open for each visitor of a site, while a closed survey is only open to a sample which the investigator knows (password-protected survey).	Page 5	Page 6	Page 8	Page 9
Contact mode	Indicate whether or not the initial contact with the potential participants was made on the Internet. (Investigators may also send out questionnaires by mail and allow for Web-based data entry.)	Page 5	Page 6	Page 8	Page 9
Advertising the survey	How/where was the survey announced or advertised? Some examples are offline media (newspapers), or	Page 5	Page 6	Page 8	Page 9

	online (mailing lists – If yes, which ones?) or banner ads (Where were these banner ads posted and what did they look like?). It is important to know the wording of the announcement as it will heavily influence who chooses to participate. Ideally the survey announcement should be published as an appendix.				
Web/E-mail	State the type of e-survey (eg, one posted on a Web site, or one sent out through e-mail). If it is an e-mail survey, were the responses entered manually into a database, or was there an automatic method for capturing responses?	Page 4-5	Page 6	Page 8	Page 10
Context	Describe the Web site (for mailing list/newsgroup) in which the survey was posted. What is the Web site about, who is visiting it, what are visitors normally looking for? Discuss to what degree the content of the Web site could pre-select the sample or influence the results. For example, a survey about vaccination on a anti-immunization Web site will have different results from a Web survey conducted on a government Web site	Page 4-5	Page 6	Page 8	Page 9
Mandatory/voluntary	Was it a mandatory survey to be filled in by every visitor who wanted to enter the Web site, or was it a voluntary survey?	Page 5	Page 6	Page 8	Page 9
Incentives	Were any incentives offered (eg, monetary, prizes, or non-monetary incentives such as an offer to provide the survey results)?	Page 5	Page 7	Page 8	Page 9

Time/Date	In what timeframe were the data collected?	Page 4	Pages 6-7	Page 8	Page 9
Randomization of items or questionnaires	To prevent biases items can be randomized or alternated.	Not applicable	Not applicable	Not applicable	Page 10
Adaptive questioning	Use adaptive questioning (certain items, or only conditionally displayed based on responses to other items) to reduce number and complexity of the questions.	Page 4	Page 6	Page 8	Not applicable
Number of Items	What was the number of questionnaire items per page? The number of items is an important factor for the completion rate.	Multimedia Appendix 1	Page 6	Page 8	Page 9
Number of screens (pages)	Over how many pages was the questionnaire distributed? The number of items is an important factor for the completion rate.	Multimedia Appendix 1	Page 6	Page 8	Page 9
Completeness check	It is technically possible to do consistency or completeness checks before the questionnaire is submitted. Was this done, and if “yes”, how (usually JAVAScript)? An alternative is to check for completeness after the questionnaire has been submitted (and highlight mandatory items). If this has been done, it should be reported. All items should provide a non-response option such as “not applicable” or “rather not say”, and selection of one response option should be enforced.	Multimedia Appendix 1	Multimedia Appendix 3	Page 14	Page 9 Eligibility Screen
Review step	State whether respondents were able to review and change their answers (eg, through a Back button or a Review step which displays a summary of the responses and asks the	Multimedia Appendix 1	Page 6	Not applicable	Page 9

	respondents if they are correct).				
Unique site visitor	If you provide view rates or participation rates, you need to define how you determined a unique visitor. There are different techniques available, based on IP addresses or cookies or both.	Page 5	Page 13	Not applicable	Not applicable
View rate (Ratio of unique survey visitors/unique site visitors)	Requires counting unique visitors to the first page of the survey, divided by the number of unique site visitors (not page views!). It is not unusual to have view rates of less than 0.1 % if the survey is voluntary.	Not obtained and not reported	Not obtained and not reported	Not obtained and not reported	Not obtained and not reported
Participation rate (Ratio of unique visitors who agreed to participate/unique first survey page visitors)	Count the unique number of people who filled in the first survey page (or agreed to participate, for example by checking a checkbox), divided by visitors who visit the first page of the survey (or the informed consents page, if present). This can also be called "recruitment" rate.	Page 12	Page 13	Page 14	Page 9
Completion rate (Ratio of users who finished the survey/users who agreed to participate)	The number of people submitting the last questionnaire page, divided by the number of people who agreed to participate (or submitted the first survey page). This is only relevant if there is a separate "informed consent" page or if the survey goes over several pages. This is a measure for attrition. Note that "completion" can involve leaving questionnaire items blank. This is not a measure for how completely questionnaires were filled in. (If you need a measure for this, use the word "completeness rate".)	Page 12	Page 13	Page 14	Page 9

Cookies used	Indicate whether cookies were used to assign a unique user identifier to each client computer. If so, mention the page on which the cookie was set and read, and how long the cookie was valid. Were duplicate entries avoided by preventing users access to the survey twice; or were duplicate database entries having the same user ID eliminated before analysis? In the latter case, which entries were kept for analysis (eg, the first entry or the most recent)?	Page 5	No cookies used.<br>Page 7	No cookies used.<br>Page 8-9	Page 9
IP check	Indicate whether the IP address of the client computer was used to identify potential duplicate entries from the same user. If so, mention the period of time for which no two entries from the same IP address were allowed (eg, 24 hours). Were duplicate entries avoided by preventing users with the same IP address access to the survey twice; or were duplicate database entries having the same IP address within a given period of time eliminated before analysis? If the latter, which entries were kept for analysis (eg, the first entry or the most recent)?	Page 5	Not used	Page 13	Page 10
Log file analysis	Indicate whether other techniques to analyze the log file for identification of multiple entries were used. If so, please describe.	Not reported/Not applicable	Not reported/Not applicable	Not reported/Not applicable	Not reported/Not applicable
Registration	In “closed” (non-open) surveys, users need to login first and it is easier	Not applicable	Not applicable	Not applicable	Page 10

	to prevent duplicate entries from the same user. Describe how this was done. For example, was the survey never displayed a second time once the user had filled it in, or was the username stored together with the survey results and later eliminated? If the latter, which entries were kept for analysis (eg, the first entry or the most recent)?				
Handling of incomplete questionnaires	Were only completed questionnaires analyzed? Were questionnaires which terminated early (where, for example, users did not go through all questionnaire pages) also analyzed?	Page 11	Page 13	Page 14	Study recruitment ongoing
Questionnaires submitted with an atypical timestamp	Some investigators may measure the time people needed to fill in a questionnaire and exclude questionnaires that were submitted too soon. Specify the timeframe that was used as a cut-off point, and describe how this point was determined.	Multimedia Appendix 1	Page 6	Page 14	Page 10
Statistical correction	Indicate whether any methods such as weighting of items or propensity scores have been used to adjust for the non-representative sample; if so, please describe the methods.	Page 11-12	Not reported	Not reported	Analysis of RCT not done yet as trial is ongoing

This checklist has been modified from Eysenbach G. Improving the quality of Web surveys: the Checklist for Reporting Results of Internet E-Surveys (CHERRIES). J Med Internet Res. 2004 Sep 29;6(3):e34 [erratum in J Med Internet Res. 2012; 14(1): e8.]. Article available at https://www.jmir.org/2004/3/e34/; erratum available https://www.jmir.org/2012/1/e8/. Copyright ©Gunther Eysenbach. Originally published in the Journal of Medical Internet Research, 29.9.2004 and 04.01.2012.

Table S3: Fraud Detection Criteria for Case Study 1 (CAPS2019), Sensitivity and Specificity Examples and Piloting Results

Criteria	Description	Example	Sensitivity	Specificity	Interpretation and piloting comments
Nonsensical or non-probable	Questions and question combinations that could indicate fraudulent responses, either because they were nonsensical (not possible within regulatory or medical context) or non-probable (unlikely true within regulatory and medical context). Assessed on R Statistical Software.	Respondents received positive fraud points if their medical specialty did not match their professional certifying organization	54.5%	99.1%	The majority of the questions and question combinations selected remained after piloting. We assigned a high number of positive fraud points due to its specificity. Due to lack of sensitivity high number of positive fraud points were weighted more heavily in the final algorithm than low number of points.
		Respondents received positive fraud points when answers between questions were contradictory (e.g. method of abortion provided).	9.1%	99.1%	As above
Free-text	Answering selected non-mandatory free-text questions was considered protective. Assessed both manually and with R Statistical Software.	Respondents received negative fraud points when they gave an answer to a free-text question that asked: "What impacts has	92.7%	100%	Assigned high number of negative (protective) points due to high sensitivity and specificity and heavily

		COVID-19 had on your individual abortion practice and/or access to abortion in your province?"			weighted in algorithm.
Time-to-complete	A short length of time taken to complete select survey sections as well as short time interval between completed surveys was considered a risk of fraud. Assessed with R Statistical Software.	Respondents received positive fraud points if they finished the Demographics Survey in the fastest quartile.	50%	33.0%	Not used in final algorithm due to low sensitivity and specificity
Non-mandatory	Within select survey sections, a low number of answered non-mandatory questions was considered a risk of fraud. Assessed with R Statistical Software.	Respondents received positive fraud points if they answered fewer than 5 non-mandatory questions in the Demographics Survey.	0%	100%	Not used in final algorithm due to low sensitivity. As many valid respondents skipped non-mandatory questions, this criterion was not effective at identifying fraud.
Survey submission date criteria	Respondents who participated after Oct 6th, 2020, the date after we noted an increase in suspicious responses, were considered at increased risk of fraud. Assessed with R Statistical Software.	A respondent who completed the survey on August 5th, 2020 was more protected when running the fraud detection algorithm than a respondent who completed the survey on for example	NA	NA	This remained in the final fraud detection algorithm as none of our positive controls[1] completed the survey prior to October 6th, 2020.

		November 15th, 2020.			
Remuneration	This criterion included: the email address provided in the remuneration survey; what respondents wanted to be contacted for in the future (options: remuneration, future research, study results); whether respondents confirmed eligibility via email after we stopped automatic remuneration; and if/when respondents redeemed their gift cards. How we summarized the remuneration criteria is outlined in Appendix 2. Assessed manually.	Respondents received positive fraud points if they requested to be contacted for remuneration but did not request to be contacted for future research or study results.	100%	89.0%	Due to high sensitivity and specificity, we heavily weighted this criterion.

[1]Respondents with highly-suspicious pattern based emails were designated as positive controls and those with known or institution emails as negative controls

[2]Registered through the Canadian Abortion Provider Support Network

Figure S1. Case Study 1 (CAPS2019) final two-phased fraud detection and categorization algorithm

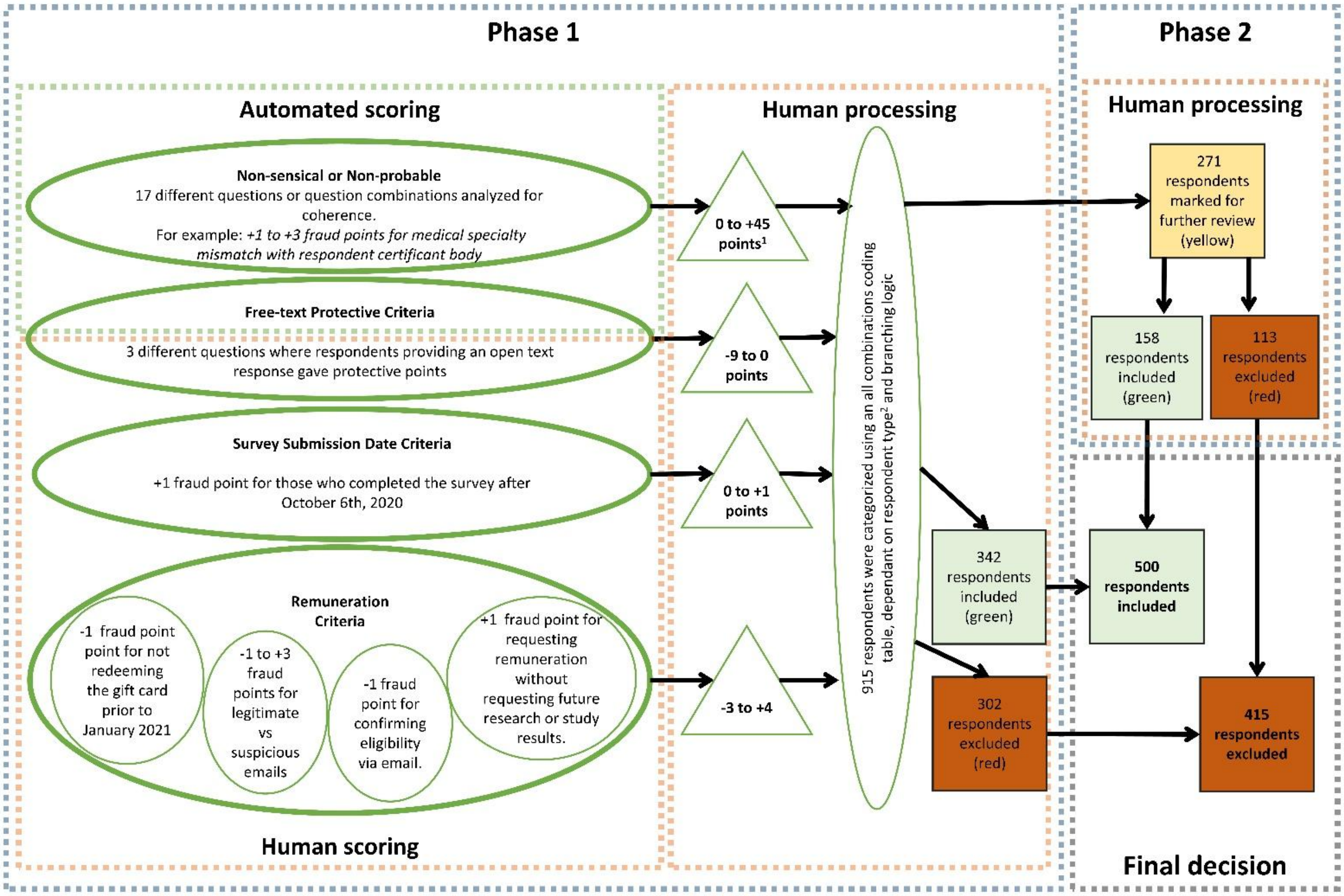

Supplement: Multimedia Appendix 1 [file jmir_v27i1e78671_app1.pdf]
