# Supplementary material for: Exploring Methods to Mitigate Fraud in Web-Based Surveys: Multicase Study Analysis
Source: J Med Internet Res. 2025 Dec 1;27:e78671. doi: 10.2196/78671 (PMC12706441; doi:10.2196/78671)
Supplement: Multimedia Appendix 2 [file jmir_v27i1e78671_app2.pdf]

# Consent Statement for Canadian Abortion Provider Survey (CAPS) 2019 Participants

Time survey opened ________________________________

Principal Investigators Regina Renner, MD, MPH, FRCSC, FACOG, Clinical Associate Professor Department of Obstetrics and Gynaecology, University of British Columbia, T: 1-877-922-7890

regina.renner@ubc.ca, cart-grac.ubc.ca

Marie-Soleil Wagner, MD, MSc, FRCSC, FACOG, Clinical Associate Professor, Department of Obstetrics and Gynaecology, University of Montreal, T: 1-877-922-7890

marie-soleil.wagner.hsj@ssss.gouv.qc.ca

Co-Investigators Jon Barrett, MBBch, FRCOG, MD, FRCSC, University of Toronto

Stephanie Begun, PhD, MSW, RSW, University of Toronto

Melissa Brooks, MD, FRCSC, Dalhousie University

Denise Bryant-Lukosius, RN, CON(C), BScN, MScN, PhD, McMaster University

Damien Contandriopoulos, BSc, MSc, PhD, University of Victoria

Elizabeth Darling, BArtsSc (Hons), BHSc, MSc, PhD, McMaster University

Brigid Dineley, MD, MHS, FRCSC, University of British Columbia

Sheila Dunn, MD, MSc, CCFP (EM), FCFP, University of Toronto

Roopan Gill, MD, MPH, FRCSC, University of British Columbia

Edith Guilbert, MD, MSc, Laval University

Janusz Kaczorowski, MA, PhD, University of Montreal

Ruth Martin-Misener, NP, PhD, Dalhousie University

Kim McGrail, MPH, PhD, University of British Columbia

Sarah Munro, PhD, University of British Columbia

Wendy Norman, MD, FCFP, DTM&H, MHSc, University of British Columbia

Helen Pymar, MD, FRCSC, MPH, University of Manitoba

Sponsors Canadian Institutes of Health Research (CIHR)

Invitation and Study Purpose You are being invited to take part in this survey as a research project conducted by the Contraception and Abortion Research Team-Groupe de recherche sur l'avortement et la contraception (CART-GRAC-http://www.cart-grac.ca/). CART/GRAC is a collaborative national network that conducts research to improve family planning health services in Canada.

By conducting this cross-sectional online survey we aim to explore the changes in medical and surgical abortion services workforce and clinical practices compared to our 2012 survey in Canada; particularly in relationship to the 2017 introduction of mifepristone medical abortion, to policy changes, changes in scope of practice for nurse practitioners that allow them now to independently provide medical abortion and changes to updated guidelines.

This survey is part of a series begun in 1997 about the practices and opinions of abortion providers. Results from these surveys have both informed clinical practice and directed research questions. 2012 survey results were published in the journal Contraception in 2016 (2016;62: e201-e208). Some of you may have answered the 2012 iteration of this survey. It has now been more than seven years since the last comprehensive survey; so, your participation in the 2019 revised survey is vital to enable us to describe important changes in abortion practice since 2012.

Our goal is to provide high-quality pan-Canadian survey data to inform abortion care planning for leaders of health policy, systems, services, and professional organizations in order to ensure and improve equitable high-quality abortion care in Canada.

Inclusion criteria: We invite you to participate in the survey if you meet all of the following inclusion criteria:

Are a physician or nurse practitioner (NP) providing abortion care who has completed your professional training (school, residency, or fellowship)

OR

Are an abortion service administrator such as program manager or medical director or operation lead

AND

Have provided abortion care for a live embryo/fetus/pregnancy in 2019, as described below:

Have prescribed at least one first trimester medical abortion functioning as an independent MRP (most responsible provider)

OR

Have performed at least one surgical abortion as an independent MRP (most responsible provider)

OR

Have provided at least one second or third trimester medical abortion functioning as an independent MRP (most responsible provider)

OR

Have provided administrative support for abortion services

AND

Are able to read and write in English or French

Study Procedures If you chose to complete the consent statement you will be invited to complete an online survey using REDCap. REDCap is a secure web platform for building and managing online databases and surveys.

The survey has the following sections (estimated time to complete the section): Section 1   Demographics (5 min)

Section 2   Clinical abortion practices:

- First trimester medical abortion (FTMA) (15 min)
- First trimester surgical abortion (FTSA) (15 min)
- Second trimester surgical abortion (STSA) (15 min)
- Second/Third trimester medical abortion (STMA; Induction of labour) (10 min)  Section 3   Administrator (10 min)

Section 4   Diverse populations (5 min)

Section 5   Stigma and resilience (Experiences as a provider or administrator) (5 min)

Section 6   Remuneration and future research (1 min)

You will only see the questions that apply to the services that you indicate you are providing. All participants will be asked to complete sections 1, 4, 5 and 6. Clinicians will be asked to complete section 2. The greater the range of abortion care a participant provides (covered in section 2), the longer it will take to complete the survey. Administrators will be asked to complete section 3 instead of section 2.

Study Results You can be directly sent a copy of the study results. The study results will be published in academic journals and presented at professional and academic conferences. In any publication or presentation, we will report information in such a way that you cannot be identified. Publications captured from this study will be available online on the CART-GRAC website (www.cart-grac.ubc.ca).

Potential Risks of the Study To reduce the risk of inadvertent disclosure of your survey response and your identity, we are taking multiple steps. Please see confidentiality section of further information. The survey includes a section on stigma and resilience. If you were to feel distress after answering this section please find here some national resources which are also included at the end of the stigma and resilience survey section:

1. CART-GRAC: https://cart-grac.ubc.ca/

2. Action Canada: https://www.actioncanadashr.org/

Access Line number: 1-888-642-2725

3. National Abortion Federation: https://prochoice.org/

Potential Benefits of the Study The survey results will allow you to learn about practice patterns across Canada and compare your practices with those of other abortion providers in Canada. In addition, your answers will inform new guidelines, policy makers and researchers in reproductive health.

Confidentiality All of your data will be stored in a database located on a Research Electronic Data Capture's (REDCap) private, relational MySQL Database at the data centre which is located on-site at the BC Children's Hospital Research Institute (BCCHRI) in Vancouver, BC. BCCHRI Information Technology is responsible for creating REDCap database backups and these backups are stored at Iron Mountain Canada, ensuring that all data and backups are stored in Canada. The Clinical Research Support Unit (CRSU) stores study data in a secure, firewall protected server with only the https port available to internet. There is a web application server that is the only gate to connect to the Database server, where the information is stored.

The only personal identifier that will be collected is your e-mail address, which we ask for in order to provide you with a gift certificate after you complete the survey, or in case they would like to be contacted either with study results or for future research. REDCap does not collect IP addresses. Personal identifiers collected on the survey are stored in a separate project within REDCap. Only the Principal Investigator (PI) and the designate will be given permission to see the personal identifier. We will export personal identifiers from the REDCap BCCHRI server weekly and store them in an encrypted file, in a restricted access folder on the "W" drive which is stored on the secured PHSA server.

The remainder of the survey data will be collected in its own project within REDCap and only the PI, designate and the data analyst will have access to the anonymized data. Once the survey is closed we export this data from the REDCap BCCHRI server and will store it in a format ready for analysis, in an encrypted file, in a restricted access folder on the "W" drive which is stored on the secured PHSA server. Only the PI, designate and the data analyst will have access to this anonymized data. No survey data set will include personal information.

No data will be published that would allow individual facilities or providers to be identified. In case of small numbers, groups of analysis will be combined into larger groups to prevent disclosure of your identity.

You are able to stop the survey at any time by clicking “Save and Return Later” at the bottom of the page. You will be provided with a pop-up of a randomly generated 8-character return code, which is needed to return to the survey. You get the following information on a page with 3 options:

1) You can click “Continue Survey Now”

OR

2) You can bookmark the page to return to the survey later. When you return, you will be able to input your code and continue at the point at which you left off.

OR

3) You can choose to have the survey link e-mailed to you by providing your e-mail address. The return code will not be included in the e-mail and your e-mail addresses will not be stored. When you return, you will be able to input your code and continue at the point at which you left off.

Any e-mail communication to you will come from a secure e-mail address. If you provide us with your personal e-mail address, please be aware that your e-mail platform might be an unsecured e-mail that we will be communicating to.

Remuneration At the end of the survey we offer you remuneration in form of a $50.00 CAD Amazon gift certificate which we will send via e-mail if you provide us with your e-mail address.

Contact for Information about the Study If you have any questions about this survey, please contact Dr. Renner or Dr. Wagner, the Canadian Lead Co-Principal Investigators. Their contact information is listed at the top of this form.

Contact for Complaints about the Study If you have any concerns or complaints about your rights as a research participant and/or your experiences while participating in this study, contact the Research Participant Complaint Line in the University of British Columbia Office of Research Ethics by e-mail at RSIL@ors.ubc.ca or by phone at

604-822-8598 (Toll Free: 1-877-822-8598). Please reference the study number (H18-03313) when contacting the Complaint Line so the staff can better assist you.

Participant Consent and Signature Page Your consent indicates that:

You have read and understood the participant information and consent statement.

You consent to participate in this study.

Taking part in this study is entirely up to you. You have the right to refuse to participate in this study. If you decide to take part, you may choose to pull out of the study at any time without giving a reason and without any negative consequences.

You have been offered a copy of this consent statement for your own records.

You are not waiving any of your legal rights as a result of signing this consent statement.

By selecting yes, you indicate that you have read and understood the information above and consent to participate in the study. The survey will now begin.

---

Do you consent to this study?

○ Yes
○ No

---

You may download a copy of this consent here.

[Attachment: "CAPS2019 Consent Statement Version 4 2020-07-13- clean.pdf"]

# Demographics

Please answer this section of the survey in regards to your demographcis.

This section will take you approximately 5 minutes to complete.

Thank you very much for participating in this survey.

---

Time survey opened ________________________________

---

You are able to stop the survey at any time by clicking "Save & Return Later" at the bottom of each page. You will be provided with a pop-up of a section-specific randomly generated 8-character return code. To return to the survey, you will need both the return code for the specific section and the survey return link.

We suggest that you retrieve your return code and link now and at the beginning of each subsequent section in case of any technical issues which may result in disconnection from the survey prior to completion.

Click here to download "Save & Return Later" instructions

---

Have you completed this 2019 Canadian Abortion Provider Survey before?

◯ Yes
◯ No

**This survey explores abortion services provided throughout all of 2019 and therefore, prior to COVID-19 restrictions. Since it can be tough to recall experiences, we kindly suggest that before beginning this survey, you take thirty seconds to picture yourself in 2019.**

**Where did you work? What did your clinical practice look like?**

Did you provide any of the following abortion care in 2019 for a live embryo/fetus/pregnancy (rather than management of a miscarriage/intrauterine fetal demise (IUFD))? (Check all that apply)

- ☐ Provide first and/or second and/or third trimester medical abortion
- ☐ Perform first and/or second trimester surgical abortion
- ☐ Administrative support for medical or surgical abortion care
- ☐ None of the above

**Please answer the remainder of this survey in regards to your practice of abortion care (first and/or second and/or third trimester) excluding management of miscarriage/IUFD**

What describes your role(s) in abortion care? (Check all that apply)

☐ Clinician (physician, nurse practitioner) who has completed their professional training (school, residency, fellowship)
☐ Administrator
☐ None of the above

What is your profession/license to practice? (Check one)

◯ Physician
◯ Nurse practitioner (NP)
◯ None of the above

Are you in training (i.e. as a medical student, resident, fellow, or nurse practitioner)?

◯ Yes
◯ No

What is your primary specialty? (Check one)

◯ General OB/GYN
◯ OB/GYN with Maternal-fetal medicine subspecialization/Perinatology
◯ Emergency Medicine
◯ Family Medicine/Family Practice/General Practice
◯ Pediatrics
◯ Other

Please specify, other specialty:

__________________________________

Are you currently a certificant of the following organizations? (Check all that apply)

☐ Certificant of the College of Family Physicians of Canada (CCFP)
☐ Fellowship in the College of Family Physicians (FCFP)
☐ Fellow of the Royal College of Physicians and Surgeons of Canada (FRCSC, FRCPC)
☐ None of the above

Please explain, none of the above:

__________________________________

Are you currently a member of the following organizations? (Check all that apply)

☐ Canadian Nurses Association
☐ Nurse Practitioner Association of Canada
☐ I don't know
☐ Other

Please specify, other:

__________________________________

Do you independently prescribe or dispense a medication for FIRST trimester medical abortion as the most responsible provider (MRP)?

◯ Yes
◯ No

What type(s) of abortion have you provided (as either the prescriber, surgeon, or physician providing labour induction) in 2019? (Check all that apply)

- ☐ FIRST trimester medical abortion (≤ 10+0 weeks or 70 days of gestation)
- ☐ FIRST trimester surgical abortion (≤ 13+6 weeks of gestation)
- ☐ SECOND trimester surgical abortion (≥ 14+0 weeks of gestation)
- ☐ SECOND trimester medical abortion (≥ 14+0 - ≤ 23+6 weeks of gestation)
- ☐ THIRD trimester medical abortion (≥ 24+0 weeks of gestation)
- ☐ None of the above

What best describes your role in the care team for FIRST trimester medical abortion? (Check all that apply)

- ☐ Independently prescribe or dispense a medication for FIRST trimester medical abortion as the most responsible provider (MRP)
- ☐ Support FIRST trimester medical abortion provision by MIDWIVES under a WRITTEN or VERBAL collaboration/inter-professional care agreement
- ☐ Support FIRST trimester medical abortion provision by REGISTERED NURSES under a WRITTEN or VERBAL collaboration/inter-professional care agreement
- ☐ Support FIRST trimester medical abortion provision by NURSE PRACTITIONERS under a WRITTEN or VERBAL collaboration/inter-professional care agreement
- ☐ Other

Please specify, other role:

__________________________________

How old are you?

__________________________________
(years)

How would you describe your gender? (Check one)

- ◯ Woman
- ◯ Man
- ◯ Transgender
- ◯ Prefer not to say
- ◯ Other

Please specify, other gender:

__________________________________

Please specify the province or territory of your PRIMARY practice (practice where you perform most of your clinical activities). (Check one)

- ◯ Alberta
- ◯ British Columbia
- ◯ Manitoba
- ◯ New Brunswick
- ◯ Newfoundland and Labrador
- ◯ Nova Scotia
- ◯ Northwest Territories
- ◯ Nunavut
- ◯ Ontario
- ◯ Prince Edward Island
- ◯ Quebec
- ◯ Quebec: Nunavik or Cree First Nations of James Bay
- ◯ Saskatchewan
- ◯ Yukon
- ◯ I do not work in Canada

What are the first three digits of the postal code of your PRIMARY practice (e.g. G3N)?

__________________________________
(first three digits of postal code)

| | |
|---|---|
| Do you provide abortion services in any ADDITIONAL clinical location(s) in the same province? | ◯ Yes<br>◯ No |
| What are the first three digits of the postal code of your ADDITIONAL practice(s) in the same province? (e.g. G3N, G2A) | ______________________<br>(first three digits of postal code) |
| Do you provide abortion services in any other province or territory? | ◯ Yes<br>◯ No |
| In which ADDITIONAL province(s) or territory/territories do you provide abortion services? (Check all that apply) | ☐ Alberta<br>☐ British Columbia<br>☐ Manitoba<br>☐ New Brunswick<br>☐ Newfoundland and Labrador<br>☐ Nova Scotia<br>☐ Northwest Territories<br>☐ Nunavut<br>☐ Ontario<br>☐ Prince Edward Island<br>☐ Quebec<br>☐ Quebec: Nunavik or Cree First Nations of James Bay<br>☐ Saskatchewan<br>☐ Yukon |
| What are the first three digits of the postal code of your ADDITIONAL practice(s) in Alberta (e.g. G3N, G2A)? | ______________________<br>(first three digits of postal code) |
| What are the first three digits of the postal code of your ADDITIONAL practice(s) in British Columbia (e.g. G3N, G2A)? | ______________________<br>(first three digits of postal code) |
| What are the first three digits of the postal code of your ADDITIONAL practice(s) in Manitoba (e.g. G3N, G2A)? | ______________________<br>(first three digits of postal code) |
| What are the first three digits of the postal code of your ADDITIONAL practice(s) in New Brunswick (e.g. G3N, G2A)? | ______________________<br>(first three digits of postal code) |
| What are the first three digits of the postal code of your ADDITIONAL practice(s) in Newfoundland and Labrador (e.g. G3N, G2A)? | ______________________<br>(first three digits of postal code) |
| What are the first three digits of the postal code of your ADDITIONAL practice(s) in Nova Scotia (e.g. G3N, G2A)? | ______________________<br>(first three digits of postal code) |
| What are the first three digits of the postal code of your ADDITIONAL practice(s) in the Northwest Territories (e.g. G3N, G2A)? | ______________________<br>(first three digits of postal code) |
| What are the first three digits of the postal code of your ADDITIONAL practice(s) in Nunavut (e.g. G3N, G2A)? | ______________________<br>(first three digits of postal code) |

What are the first three digits of the postal code of your ADDITIONAL practice(s) in Ontario (e.g. G3N, G2A)?

__________________ (first three digits of postal code)

What are the first three digits of the postal code of your ADDITIONAL practice(s) in Prince Edward Island (e.g. G3N, G2A)?

__________________ (first three digits of postal code)

What are the first three digits of the postal code of your ADDITIONAL practice(s) in  Quebec (e.g. G3N, G2A)?

__________________ (first three digits of postal code)

What are the first three digits of the postal code of your ADDITIONAL practice(s) in Quebec: Nunavik or Cree First Nations of James Bay (e.g. G3N, G2A)?

__________________ (first three digits of postal code)

What are the first three digits of the postal code of your ADDITIONAL practice(s) in Saskatchewan (e.g. G3N, G2A)?

__________________ (first three digits of postal code)

What are the first three digits of the postal code of your ADDITIONAL practice(s) in the Yukon (e.g. G3N, G2A)?

__________________ (first three digits of postal code)

About what percentage of your clinical work focuses on contraception and abortion?

__________________ (%)

How many clinicians (physicians or nurse practitioners) that you know of provide the following services in the city/town where you have your PRIMARY practice?
(Please estimate number of clinicians for each option, including yourself. If you cannot provide an estimate, please specify below)

a. FIRST trimester medical abortion (≤ 10+0 weeks or 70 days of gestation)

__________________ (number of clinicians)

b. FIRST trimester surgical abortion (≤ 13+6 weeks of gestation)

__________________ (number of clinicians)

c. SECOND trimester surgical abortion (≥ 14+0 weeks of gestation)

__________________ (number of clinicians)

d. SECOND trimester medical abortion (≥ 14+0 - ≤ 23+6 weeks of gestation)

__________________ (number of clinicians)

e. THIRD trimester medical abortion (≥ 24+0 weeks of gestation)

__________________ (number of clinicians)

Please explain why you could not provide an estimate for how many physicians and/or nurse practitioners provide the above services in your city/town.

__________________

| | |
|---|---|
| Please specify which guidelines you follow to provide abortion care? (Check all that apply) | ☐ Society of Obstetricians & Gynaecoloigsts of Canada  (SOGC)<br>☐ National Abortion Federation (NAF, Clinicial Policy Guideline 2018)<br>☐ I don't know<br>☐ Other |
| Please specify, other | __________________________________________ |

# First Trimester Medical Abortion (FTMA)

Please answer this section of the survey in regards to your practice of induced FIRST trimester medical abortion care EXCLUDING management of miscarriage.
This section will take you approximately 15 minutes to complete.
Thank you very much for participating in this survey.

---

Time survey opened

__________________________________

---

Survey progress (%)

---

You are able to stop the survey at any time by clicking "Save & Return Later" at the bottom of each page. You will be provided with a pop-up of a section-specific randomly generated 8-character return code. To return to the survey, you will need both the return code for the specific section and the survey return link.

We suggest that you retrieve your return code and link now and at the beginning of each subsequent section in case of any technical issues which may result in disconnection from the survey prior to completion.

Click here to download "Save & Return Later" instructions

---

What is the total number of FIRST trimester medical abortions in the calendar year 2019? (Please estimate for each option)

---

You signed the prescription or dispensed independently as the most responsible provider (MRP)

__________________________________
(number of FIRST trimester medical abortions in 2019)

---

For which you supported nurse practitioners or registered nurses in providing

__________________________________
(number of FIRST trimester medical abortions in 2019)

---

For which you supported midwives in providing

__________________________________
(number of FIRST trimester medical abortions in 2019)

---

What is the total number of FIRST trimester medical abortions in the calendar year 2019 you signed a prescription or dispensed independently as the most responsible provider (MRP)?

__________________________________
(number of FIRST trimester medical abortions in 2019)

---

In how many clinical locations do you provide FIRST trimester medical abortion care (e.g. 1 hospital and 2 clinics = 3 locations)?

__________________________________
(number of clinical locations)

---

Please specify the clinical location of your PRIMARY practice providing FIRST trimester medical abortion care. (Check one)

- ◯ Outside of hospital
- ◯ Community hospital
- ◯ Academic hospital
- ◯ Other

---

Please specify, other:

__________________________________

| | |
|---|---|
| Does this PRIMARY clinical location outside of the hospital provide any services other than abortion care? (Check all that apply) | ☐ Yes, other reproductive health care<br>☐ Yes, general health care other than reproductive health care<br>☐ No, only abortion services |
| What best describes how your PRIMARY hospital-based clinical location provides abortion care? (Check one) | ◯ Provides organized abortion only service/dedicated abortion clinic<br>◯ Provides abortion care mixed in with other medical care |
| Does this PRIMARY clinical location provide any services other than abortion care? (Check one) | ◯ Yes, other reproductive health care<br>◯ Yes, general health care other than reproductive health care<br>◯ No, only abortion services |
| Please specify the clinical location(s) of your ADDITIONAL practice(s) providing FIRST trimester medical abortion care. (Check all that apply) | ☐ Outside of hospital<br>☐ Community hospital<br>☐ Academic hospital<br>☐ Other |
| Please specify, other: | __________________________________ |
| Does this (do these) ADDITIONAL clinical location(s) outside of the hospital provide any services other than abortion care? (Check all that apply) | ☐ Yes, other reproductive health care<br>☐ Yes, general health care other than reproductive health care<br>☐ No, only abortion services |
| What best describes how your ADDITIONAL hospital-based clinical location(s) provides abortion care? (Check all that apply) | ☐ Provides organized abortion only service/dedicated abortion clinic<br>☐ Provides abortion care mixed in with other medical care |
| Does this (do these) ADDITIONAL other clinical location(s) provide any services other than abortion care? (Check all that apply) | ☐ Yes, other reproductive health care<br>☐ Yes, general health care other than reproductive health care<br>☐ No, only abortion services |
| How many years of experience do you have providing FIRST trimester medical abortions (with a METHOTREXATE/MISOPROSTOL and/or MIFEPRISTONE/MISOPROSTOL and/or MISOPROSTOL-ONLY regimen) after completion of training (e.g. number of years after residency or fellowship or after nurse practitioner education)? (Check one) | ◯ < 5 years<br>◯ 5 to 10 years<br>◯ 11 to 15 years<br>◯ 16 to 20 years<br>◯ > 20 years |

**If you provide services in more than one clinical location, please answer the subsequent questions in regards to the clinical location you provide most of your abortion care at.**

Under which of the following circumstances do you usually provide FIRST trimester medical abortion (in the absence of a risk factor for OR a clinical symptom of an ectopic pregnancy)? (Check all that apply)

- ☐ Patient less than 18 years of age
- ☐ Patient who lives > 2 hours away from access to an emergency D&C (dilation & aspiration)
- ☐ Patient who lives > 2 hours from an emergency unit
- ☐ Patient self-referred
- ☐ Patient referred to you from outside of your office/clinic
- ☐ Patient referred to you by a colleague inside your clinic
- ☐ Patient living in another town/city than the one where you provide abortion services
- ☐ Patient travelling from far away (more than 50 km)
- ☐ None of the above
- ☐ Other

Please specify, other: __________________________________

Which do you consider to be a risk factor(s) for OR a clinical symptom(s) of an ectopic pregnancy? (Check all that apply)

- ☐ Previous ectopic pregnancy
- ☐ Previous tubal surgery
- ☐ Pregnancy conceived with assisted reproductive techniques
- ☐ Prior tubal ligation
- ☐ IUD (intrauterine device) in place
- ☐ Prior history of salpingitis or pelvic inflammatory disease (PID)
- ☐ Abdominal pain during first trimester
- ☐ Vaginal bleeding during first trimester
- ☐ All of the above
- ☐ None of the above

For which indication(s) do you usually provide FIRST trimester medical abortion in a live embryo/fetus? (Check all that apply)

- ☐ Patient request without other factors
- ☐ Genetic anomaly not compatible with life
- ☐ Any genetic anomaly
- ☐ Congenital anomaly/malformation not compatible with life
- ☐ Any congenital anomaly/malformation
- ☐ Maternal medical indication
- ☐ All of the above
- ☐ Other

Please specify, other: __________________________________

In which case(s) do you usually require a pre-procedure ultrasound before FIRST trimester medical abortion? (Check all that apply)

- ☐ In all patients
- ☐ In case of unsure last menstrual period (LMP)
- ☐ In case of discrepancy between physical exam and LMP
- ☐ In case of a risk factor for or a clinical symptom of an ectopic pregnancy
- ☐ Never
- ☐ Other

Please specify, other: __________________________________

| | |
|---|---|
| Where do you usually access ultrasound for your FIRST trimester medical abortion patients? (Check all that apply) | ☐ Through an ultrasound in my clinic<br>☐ Through diagnostic imaging in my health region or hospital/radiology<br>☐ Through maternal-fetal medicine/perinatology in my health region or hospital<br>☐ Other |
| Please specify, other: | __________________ |
| Do you experience barriers to obtain an ultrasound for your FIRST trimester medical abortion patients? | ◯ No<br>◯ Yes |
| Please specify, yes: | __________________ |
| Who usually performs ultrasound for your FIRST trimester medical abortion patients? (Check all that apply) | ☐ Trained non-licensed staff<br>☐ Licensed sonogram technician/radiology technician<br>☐ Licensed practical nurse (LPN)<br>☐ Registered nurse (RN)<br>☐ Nurse practitioner (NP)<br>☐ Physician assistant (PA) [Not applicable to Quebec]<br>☐ Midwife<br>☐ Physician (non-Radiologist)<br>☐ Physician (Radiologist)<br>☐ Me<br>☐ I don't know<br>☐ Other |
| Please specify, other: | __________________ |
| Which of the following regimens do you provide for FIRST trimester medical abortion? (Check all that apply) | ☐ Mifepristone/misoprostol<br>☐ Methotrexate/misoprostol<br>☐ Misoprostol-only for medical abortion (excluding miscarriages)<br>☐ Other |
| Please specify, other: | __________________ |

In approximately what percentages of your FIRST trimester medical abortions do you use the following regimens? (Please estimate percentage for each option. Please be sure that the percentages sum up to 100%)

| | |
|---|---|
| Mifepristone/misoprostol: | __________________ (%) |
| Methotrexate/misoprostol: | __________________ (%) |
| Misoprostol-only for medical abortion (excluding miscarriages): | __________________ (%) |
| Other regimen (as indicated in previous question): | __________________ (%) |

Total percentage of regimens used in FIRST trimester medical abortions:

__________________________________
(%)

Your total does not sum to 100%.

How did you receive training for FIRST trimester medical abortion with a MIFEPRISTONE/MISOPROSTOL regimen? (Check all that apply)

- ☐ Online training with Society of Obstetricians and Gynaecologists of Canada (SOGC)
- ☐ Online training with National Abortion Federation (NAF)
- ☐ Preceptorship or clinical traineeship
- ☐ Live Workshop
- ☐ None
- ☐ Other

Please specify, other:

__________________________________

Were your training needs met? (Check one)

- ◯ Yes
- ◯ No
- ◯ Prefer not to answer

Please specify, unmet training needs:

__________________________________

What is the minimum gestational age/criteria at which you provide FIRST trimester medical abortion with a MIFEPRISTONE/MISOPROSTOL regimen (in the absence of uncertainty about gestational age
AND
in the absence of a risk factor for or a clinical symptom of an ectopic pregnancy)? (Check one)

- ◯ As soon as the pregnancy test is positive, regardless of gestational age
- ◯ Starting at a certain gestational age (specify below)
- ◯ If ultrasound is performed, only after an intrauterine yolk sac or fetal pole are seen on ultrasound
- ◯ Other

Please specify, minimum gestational age (days) at which you offer FIRST trimester medical abortion:

__________________________________
(days of gestation)

Please specify, other:

__________________________________

What is/are the criteria for which you provide FIRST trimester medical abortion with a MIFEPRISTONE/MISOPROSTOL regimen in the setting of a pregnancy of unknown location (absence of yolk sac or embryo within an intrauterine gestational sac on ultrasound)
AND
in the absence of a risk factor for or a clinical symptom of an ectopic pregnancy? (Check all that apply)

- ☐ No intrauterine gestational sac is seen on ultrasound and the serum βhCG level is > 2000 IU/L
- ☐ No intrauterine gestational sac is seen on ultrasound and the serum βhCG level is ≤ 2000 IU/L
- ☐ A likely intrauterine gestational sac is seen on ultrasound without a yolk sac or fetal pole
- ☐ I do not provide first trimester medical abortion in the case of pregnancy of unknown location
- ☐ Other

Please specify, other:

__________________________________

Up to what maximum gestational age do you provide FIRST trimester medical abortion with a MIFEPRISTONE/MISOPROSTOL regimen?

__________________________________
(days of gestation)

Which factor(s) determines your upper gestational age limit for FIRST trimester medical abortion? (Check all that apply)

- ☐ Evidence-based guidelines
- ☐ Personal preference
- ☐ Clinic/Facility regulations
- ☐ Provincial/Territorial regulations
- ☐ Other

Please specify, other: ______________________

Please specify which guideline(s) you follow to determine the upper gestational age limit for abortion. (Check all that apply)

- ☐ Society of Obstetricians and Gynaecologists of Canada (SOGC)
- ☐ National Abortion Federation (NAF) Clinical Policy Guidelines 2018
- ☐ Other

Please specify, other: ______________________

Prior to prescribing a MIFEPRISTONE/MISOPROSTOL regimen for a FIRST trimester medical abortion, I assess the patient for the following relative and absolute contraindications: (Check all that apply)

- ☐ Ectopic pregnancy
- ☐ Chronic renal failure
- ☐ Inherited porphyria
- ☐ Uncontrolled asthma
- ☐ Known hypersensitivity to product ingredients
- ☐ Patient ambivalence
- ☐ Uncertain gestational age
- ☐ Intrauterine device in place
- ☐ Concurrent long-term systemic corticosteroid therapy
- ☐ Haemorrhagic disorder or using concurrent anticoagulant therapy
- ☐ All of the above
- ☐ None of the above

What route of misoprostol administration do you usually recommend when using a MIFEPRISTONE/MISOPROSTOL regimen for FIRST trimester medical abortion? (Check one)

- ○ Vaginal
- ○ Oral (swallowed)
- ○ Sublingual (under tongue)
- ○ Buccal (between the teeth and the cheek)
- ○ Other

Please specify, other route: ______________________

Under which of the following circumstances do you provide a METHOTREXATE/MISOPROSTOL regimen for FIRST trimester medical abortion? (Check all that apply)

- ☐ For all cases of medical abortion
- ☐ In case of pregnancy of unknown location (PUL) without a risk factor for or a clinical symptom of an ectopic pregnancy (no intrauterine yolk sac visible on ultrasound)
- ☐ In case of PUL with a risk factor for or a clinical symptom of an ectopic pregnancy
- ☐ In case of suspected ectopic pregnancy
- ☐ In case of patient not being able to afford MIFEPRISTONE/MISOPROSTOL combination
- ☐ Other

Please specify, other: ______________________

| | |
|---|---|
| What route of misoprostol administration do you usually recommend when using a METHOTREXATE/MISOPROSTOL regimen for FIRST trimester medical abortion? (Check one) | ◯ Vaginal<br>◯ Oral (swallowed)<br>◯ Sublingual (under tongue)<br>◯ Buccal (between the teeth and the cheek)<br>◯ Other |
| Please specify, other route: | __________________________ |
| Under which of the following circumstances do you provide a MISOPROSTOL-ONLY regimen for FIRST trimester medical abortion? (Check all that apply) | ☐ For all cases of medical abortion<br>☐ In case of pregnancy of unknown location (PUL) without a risk factor for or a clinical symptom of an ectopic pregnancy (no intrauterine yolk sac visible on ultrasound)<br>☐ In case of PUL with a risk factor for or a clinical symptom of an ectopic pregnancy<br>☐ In case of suspected ectopic pregnancy<br>☐ In case of patient not being able to afford MIFEPRISTONE/MISOPROSTOL combination<br>☐ Other |
| Please specify, other: | __________________________ |
| What misoprostol dose do you use in a MISOPROSTOL-ONLY regimen for FIRST trimester medical abortion? (Check one) | ◯ 400 mcg<br>◯ 600 mcg<br>◯ 800 mcg<br>◯ 1000 mcg |
| What misoprostol route do you usually recommend in a MISOPROSTOL-ONLY regimen for FIRST trimester medical abortion? (Check one) | ◯ Vaginal<br>◯ Oral (swallowed)<br>◯ Sublingual (under tongue)<br>◯ Buccal (between the teeth and the cheek)<br>◯ Other |
| Please specify, other route: | __________________________ |
| Do you provide FIRST trimester medical abortion to patients with a twin gestation? | ◯ Yes<br>◯ No |
| Up to what maximum gestational age do you provide FIRST trimester medical abortion to patients with a twin gestation? | __________________________<br>(days of gestation) |
| Who usually provides pre-abortion patient education/counselling for FIRST trimester medical abortion? (Check all that apply) | ☐ Unlicensed trained counsellor/educator/health care worker<br>☐ Licensed counsellor/social worker or similarly credentialed person<br>☐ Licensed practical nurse (LPN)<br>☐ Registered nurse (RN)<br>☐ Nurse practitioner (NP)<br>☐ Physician assistant (PA) [Not applicable to Quebec]<br>☐ Midwife<br>☐ Physician (attending, resident or fellow)<br>☐ Me<br>☐ Educational video<br>☐ Other |

Please specify, other:

________________________________

Who primarily discusses pre-abortion informed consent for FIRST trimester medical abortion? (Check all that apply)

- ☐ Unlicensed trained counsellor/educator/healthcare worker
- ☐ Licensed counsellor/social worker or similarly credentialed person
- ☐ Licensed practical nurse (LPN)
- ☐ Registered nurse (RN)
- ☐ Nurse practitioner (NP)
- ☐ Physician assistant (PA) [Not applicable to Quebec]
- ☐ Midwife
- ☐ Physician (attending, resident or fellow)
- ☐ Me
- ☐ Other

Please specify, other:

________________________________

For which percentage of FIRST trimester medical abortion patients do you perform or request each of the following tests? (Please estimate percentages)

a. Ultrasound

________________________________
(%)

b. Urine hCG

________________________________
(%)

c. Quantitative serum βhCG

________________________________
(%)

d. General physical exam

________________________________
(%)

e. Vaginal and pelvic/bimanual exam

________________________________
(%)

f. Past medical history

________________________________
(%)

g. Rh status

________________________________
(%)

h. Hemoglobin or hematocrit

________________________________
(%)

i. Sexually transmitted infection (STI) testing

________________________________
(%)

j. Other

________________________________
(%)

Please specify, other test(s):

________________________________

Do you assess Rh status in the following patients? (Check one)

- ◯ In all patients
- ◯ Only if gestational age is ≥ 7+0 weeks
- ◯ Only if gestational age is ≥ 8+0 weeks
- ◯ Never
- ◯ Other

Please specify, other:

________________________________

If a patient is Rh (-), do you offer Rh immune globulin administration? (Check one)

- ◯ To all patients
- ◯ Only if gestational age is ≥ 7+0 weeks
- ◯ Only if gestational age is ≥ 8+0 weeks
- ◯ Never
- ◯ Other

Please specify, other:

________________________________

Where does MIFEPRISTONE/MISOPROSTOL get dispensed to the patient? (Please estimate percentage for each option. Please be sure that the percentages sum to 100%)

At the hospital pharmacy:

________________________________
(%)

At the community pharmacy:

________________________________
(%)

In the office/clinic:

________________________________
(%)

Other:

________________________________
(%)

Please specify, other location:

________________________________

Total percentage of where MIFEPRISTONE/MISOPROSTOL gets dispensed:

________________________________
(%)

Your total does not sum to 100%.

Where does the patient take the mifepristone dose? (Please estimate percentage for each option. Please be sure that the percentages sum to 100%).

a. Mandated to be taken in the office/clinic

______________________ (%)

b. Home/outside of clinic as not available in the office/clinic

______________________ (%)

c. Per patient's preference in office/clinic

______________________ (%)

d. Per patient's preference at home/outside of clinic

______________________ (%)

e. Other

______________________ (%)

Please specify, other location:

______________________

Total percentage of where patient takes mifepristone dose:

______________________ (%)

Your total does not sum to 100%.

Survey progress (%)

Which analgesic(s) or adjunct medication(s) do you usually recommend, prescribe or dispense to FIRST trimester medical abortion patients for management of side effects? (Check all that apply)

- ☐ Acetaminophen
- ☐ Nonsteroidal anti-inflammatory drug (NSAID; e.g. Ibuprofen®)
- ☐ Opioid
- ☐ Anti-emetic
- ☐ None
- ☐ Other

Please specify, other:

______________________

What percentage of your patients receives an opioid for FIRST trimester medical abortion?

______________________ (%)

Which opioid do you usually prescribe or dispense (either by itself or combined with acetaminophen)? (Check one)

- ◯ Codeine
- ◯ Tramadol
- ◯ Hydromorphone
- ◯ Oxycodone
- ◯ Morphine
- ◯ Other

Please specify, other:

______________________

Please specify, regarding the medication chosen above, what is the usual number of pills and refills you prescribe or dispense.

Number of pills: ______________________________

Number of refills: ______________________________

Do you provide an antibiotic prophylaxis in FIRST trimester medical abortion? (Check one)

- ◯ Always
- ◯ Based on risk factors
- ◯ Never
- ◯ Other

Please specify, other: ______________________________

When do you usually recommend a follow-up contact/assessment or visit after a FIRST trimester medical abortion? (Check one for each situation)

a. For a confirmed intrauterine pregnancy

- ◯ 1-6 days after misoprostol is taken
- ◯ 7-14 days after misoprostol is taken
- ◯ 15 days or more after misoprostol is taken
- ◯ Other

Please specify, other: ______________________________

b. For a pregnancy of unknown location (absence of yolk sac or embryo within an intrauterine gestational sac on ultrasound)
AND
in the absence of a risk factor for or a clinical symptom of an ectopic pregnancy

- ◯ I do not provide first trimester medical abortion in the case of pregnancy of unknown location
- ◯ 1-6 days after misoprostol is taken
- ◯ 7-14 days after misoprostol is taken
- ◯ 15 days or more after misoprostol is taken
- ◯ Other

Please specify, other: ______________________________

How do you usually follow-up to assess if the FIRST trimester medical abortion is complete? (Check all that apply)

- ☐ Ultrasonography
- ☐ Serial serum quantitative βhCG testing
- ☐ Low sensitivity/Semi-quantitative urine hCG testing
- ☐ Physical exam
- ☐ Standardized evaluation by telephone
- ☐ Standardized evaluation by electronic communication
- ☐ Other

Please specify, other: ______________________________

When do you advise a repeat dose of misoprostol for FIRST trimester medical abortion patients using a MIFEPRISTONE/MISOPROSTOL regimen? (Check all that apply)

- ☐ Never
- ☐ If no bleeding occurs in 24 - 48 hours after the first dose of misoprostol
- ☐ For heavy bleeding
- ☐ For prolonged bleeding up to 30 days
- ☐ For SYMPTOMATIC retained nonviable sac, decidua or blood on ultrasound
- ☐ For ASYMPTOMATIC prolonged retention of a non-viable gestational sac on ultrasound
- ☐ For ASYMPTOMATIC thickened endometrial lining without gestational sac
- ☐ For ongoing viable pregnancy
- ☐ Routinely offered
- ☐ Other

Please specify, other:

__________________________________

When do you advise a repeat dose of misoprostol for FIRST trimester medical abortion patients using a METHOTREXATE/MISOPROSTOL regimen? (Check all that apply)

- ☐ Never
- ☐ If no bleeding occurs in 24 - 48 hours after the first dose of misoprostol
- ☐ For heavy bleeding
- ☐ For prolonged bleeding up to 30 days
- ☐ For SYMPTOMATIC retained nonviable sac, decidua or blood on ultrasound
- ☐ For ASYMPTOMATIC prolonged retention of a non-viable gestational sac on ultrasound
- ☐ For ASYMPTOMATIC thickened endometrial lining without gestational sac
- ☐ For ongoing viable pregnancy
- ☐ Routinely offered
- ☐ Other

Please specify, other:

__________________________________

When do you advise a repeat dose of misoprostol for FIRST trimester medical abortion patients using a MISOPROSTOL-ONLY regimen? (Check all that apply)

- ☐ Never
- ☐ If no bleeding occurs in 24 - 48 hours after the first dose of misoprostol
- ☐ For heavy bleeding
- ☐ For prolonged bleeding up to 30 days
- ☐ For SYMPTOMATIC retained nonviable sac, decidua or blood on ultrasound
- ☐ For ASYMPTOMATIC prolonged retention of a non-viable gestational sac on ultrasound
- ☐ For ASYMPTOMATIC thickened endometrial lining without gestational sac
- ☐ For ongoing viable pregnancy
- ☐ Routinely offered
- ☐ Other

Please specify, other:

__________________________________

What proportion of your FIRST trimester medical abortion patients have received a repeat dose of misoprostol (e.g. for retained products of conception) after using a MIFEPRISTONE/MISOPROSTOL regimen? (Please estimate percentage)

__________________________________
(%)

What proportion of your FIRST trimester medical abortion patients have received a repeat dose of misoprostol (e.g. for retained products of conception) after using a METHOTREXATE/MISOPROSTOL regimen? (Please estimate percentage)

__________________________________ (%)

What proportion of your FIRST trimester medical abortion patients have received a repeat dose of misoprostol (e.g. for retained products of conception) after using a MISOPROSTOL-ONLY regimen? (Please estimate percentage)

__________________________________ (%)

When do you provide a repeat course of a MIFEPRISTONE/MISOPROSTOL regimen for FIRST trimester medical abortion? (Check all that apply)

- ☐ Never
- ☐ If no bleeding occurs in 24 - 48 hours after the first dose of misoprostol
- ☐ For heavy bleeding
- ☐ For prolonged bleeding up to 30 days
- ☐ For SYMPTOMATIC retained nonviable sac, decidua or blood on ultrasound
- ☐ For ASYMPTOMATIC prolonged retention of a non-viable gestational sac on ultrasound
- ☐ For ASYMPTOMATIC thickened endometrial lining without gestational sac
- ☐ For ongoing viable pregnancy
- ☐ Routinely offered
- ☐ Other

Please specify, other:

__________________________________

When do you provide a repeat course of a METHOTREXATE/MISOPROSTOL regimen for FIRST trimester medical abortion? (Check all that apply)

- ☐ Never
- ☐ If no bleeding occurs in 24 - 48 hours after the first dose of misoprostol
- ☐ For heavy bleeding
- ☐ For prolonged bleeding up to 30 days
- ☐ For SYMPTOMATIC retained nonviable sac, decidua or blood on ultrasound
- ☐ For ASYMPTOMATIC prolonged retention of a non-viable gestational sac on ultrasound
- ☐ For ASYMPTOMATIC thickened endometrial lining without gestational sac
- ☐ For ongoing viable pregnancy
- ☐ Routinely offered
- ☐ Other

Please specify, other:

__________________________________

What proportion of your FIRST trimester medical abortion patients have received a repeat course of a MIFEPRISTONE/MISOPROSTOL regimen? (Please estimate percentage)

__________________________________ (%)

What proportion of your FIRST trimester medical abortion patients have received a repeat course of a METHOTREXATE/MISOPROSTOL regimen? (Please estimate percentage)

__________________________________ (%)

When do you provide/refer for a SURGICAL PROCEDURE after a FIRST trimester abortion with a MIFEPRISTONE/MISOPROSTOL regimen? (Check all that apply)

- ☐ Never
- ☐ If no bleeding occurs in 24 - 48 hours after the first dose of misoprostol
- ☐ For heavy bleeding
- ☐ For prolonged bleeding up to 30 days
- ☐ For SYMPTOMATIC retained nonviable sac, decidua or blood on ultrasound
- ☐ For ASYMPTOMATIC prolonged retention of a non-viable gestational sac on ultrasound
- ☐ For ASYMPTOMATIC thickened endometrial lining without gestational sac
- ☐ For ongoing viable pregnancy
- ☐ Other

Please specify, other: ______________________________

When do you provide/refer for a SURGICAL PROCEDURE after a FIRST trimester abortion with a METHOTREXATE/MISOPROSTOL regimen? (Check all that apply)

- ☐ Never
- ☐ If no bleeding occurs in 24 - 48 hours after the first dose of misoprostol
- ☐ For heavy bleeding
- ☐ For prolonged bleeding up to 30 days
- ☐ For SYMPTOMATIC retained nonviable sac, decidua or blood on ultrasound
- ☐ For ASYMPTOMATIC prolonged retention of a non-viable gestational sac on ultrasound
- ☐ For ASYMPTOMATIC thickened endometrial lining without gestational sac
- ☐ For ongoing viable pregnancy
- ☐ Other

Please specify, other: ______________________________

When do you provide/refer for a SURGICAL PROCEDURE after a FIRST trimester abortion with a MISOPROSTOL-ONLY regimen? (Check all that apply)

- ☐ Never
- ☐ If no bleeding occurs in 24 - 48 hours after the first dose of misoprostol
- ☐ For heavy bleeding
- ☐ For prolonged bleeding up to 30 days
- ☐ For SYMPTOMATIC retained nonviable sac, decidua or blood on ultrasound
- ☐ For ASYMPTOMATIC prolonged retention of a non-viable gestational sac on ultrasound
- ☐ For ASYMPTOMATIC thickened endometrial lining without gestational sac
- ☐ For ongoing viable pregnancy
- ☐ Other

Please specify, other: ______________________________

What proportion of your FIRST trimester medical abortion patients had a subsequent SURGICAL PROCEDURE for the same pregnancy after using a MIFEPRISTONE/MISOPROSTOL regimen? (Please estimate percentage)

______________________________ (%)

| | |
|---|---|
| What proportion of your FIRST trimester medical abortion patients had a subsequent SURGICAL PROCEDURE for the same pregnancy after using a METHOTREXATE/MISOPROSTOL regimen? (Please estimate percentage) | ______________ (%) |
| What proportion of your FIRST trimester medical abortion patients had a subsequent SURGICAL PROCEDURE for the same pregnancy after using a MISOPROSTOL-ONLY regimen? (Please estimate percentage) | ______________ (%) |
| In what percentage of patients do you assess for preferred post-abortion contraception method after FIRST trimester medical abortion? | ______________ (%) |

What percentage of your patients initiates a long acting reversible contraceptive (LARC)/leaves with a prescription for a short acting reversible contraceptive (SARC)/plans to use another method after a FIRST trimester medical abortion? (Please estimate percentage for each option. Please be sure that the percentages sum to 100%.)

| | |
|---|---|
| a) Long acting reversible contraceptive (LARC): intrauterine system (IUS) / intrauterine device (IUD) | ______________ (%) |
| b) Short acting reversible contraceptive (SARC): oral contraceptive pill, ring, patch, injectable contraception (e.g. depo-medroxyprogesterone acetate (DMPA)) | ______________ (%) |
| c) Barrier methods only or other methods | ______________ (%) |
| d) None | ______________ (%) |
| Total percentage of contraceptive methods: | ______________ (%) |

Your total does not sum to 100%.

**How early do you recommend to initiate the following contraceptive options after FIRST trimester medical abortion? (Check one for each option)**

| | Same day as mifepristone | The day after misoprostol | Once complete abortion has been confirmed | Other | Do not offer this method |
|---|---|---|---|---|---|
| a) Injectable contraception (e.g. DMPA) | ○ | ○ | ○ | ○ | ○ |
| b) Oral contraceptive pill (OCP) | ○ | ○ | ○ | ○ | ○ |
| c) Patch | ○ | ○ | ○ | ○ | ○ |
| d) Ring | ○ | ○ | ○ | ○ | ○ |
| e) Hormonal IUS | ○ | ○ | ○ | ○ | ○ |
| f) Copper IUD | ○ | ○ | ○ | ○ | ○ |

Please specify, other time for injectable contraception initiation: ______________________

Please specify, other time for oral contraceptive pill initiation: ______________________

Please specify, other time for patch initiation: ______________________

Please specify, other time for ring initiation: ______________________

Please specify, other time for hormonal IUS initiation: ______________________

Please specify, other time for copper IUD initiation: ______________________

**How often do you or someone else in the care team assess for the following history in patients presenting for FIRST trimester medical abortion? (Check one for each option)**

| | We/I always provide this type of assessment | We/I sometimes provide this type of assessment, based on the individual patient | We/I rarely provide this type of assessment | We/I never provide this type of assessment | Prefer not to answer |
|---|---|---|---|---|---|
| a. Intimate partner violence | ○ | ○ | ○ | ○ | ○ |
| b. Familial abuse | ○ | ○ | ○ | ○ | ○ |
| c. Mental health concerns | ○ | ○ | ○ | ○ | ○ |

What is your average wait time between the patient's first contact (phone call or referral) with your clinical location and them receiving a prescription or the medication for a FIRST trimester medical abortion? (Please estimate)

________________________________
(number of days)

**Please specify the purpose of the individual visits/phone calls (for telemedicine services) a patient experiences in order to complete a FIRST trimester medical abortion. (Check all that apply for each visit/call)**

| | 1st visit/call | 2nd visit/call | 3rd visit/call | 4th visit/call | 5th visit/call | 6th visit/call | 7th visit/call | Other visits/ calls |
|---|---|---|---|---|---|---|---|---|
| a. Counselling/consenting | ☐ | ☐ | ☐ | ☐ | ☐ | ☐ | ☐ | ☐ |
| b. Ultrasound | ☐ | ☐ | ☐ | ☐ | ☐ | ☐ | ☐ | ☐ |
| c. Blood work | ☐ | ☐ | ☐ | ☐ | ☐ | ☐ | ☐ | ☐ |
| d. Physical exam | ☐ | ☐ | ☐ | ☐ | ☐ | ☐ | ☐ | ☐ |
| e. Prescribing medication | ☐ | ☐ | ☐ | ☐ | ☐ | ☐ | ☐ | ☐ |
| f. Follow-up visit/call | ☐ | ☐ | ☐ | ☐ | ☐ | ☐ | ☐ | ☐ |
| g. Not applicable | ☐ | ☐ | ☐ | ☐ | ☐ | ☐ | ☐ | ☐ |
| h. Other purpose | ☐ | ☐ | ☐ | ☐ | ☐ | ☐ | ☐ | ☐ |

Please specify other purpose of every visit/call indicated above:

__________________________________

Please specify number and purpose of every other visit/call indicated above:

__________________________________

How do you usually provide after-hours coverage for patients undergoing FIRST trimester medical abortion? (Check all that apply)

☐ I hand out my personal phone number
☐ I am part of an abortion specific call group
☐ I am part of a general call group and ask my patients to call our on-call person
☐ I ask my patients to present to our emergency department
☐ My patients can call a central or provincial toll-free number to speak with a nurse advisor
☐ Other

Please specify, other:

__________________________

For which indication(s) do you use a MIFEPRISTONE/MISOPROSTOL regimen in FIRST trimester MISCARRIAGE management? (Check all that apply)

☐ Incomplete abortion
☐ Missed abortion (empty gestational sac or embryonic/fetal demise on ultrasound)
☐ Never
☐ Other

Please specify, other:

__________________________

In approximately what percentage of medically managed FIRST trimester MISSED ABORTIONS do you use the following regimens? (Please estimate percentage for each option. Please be sure that the percentages sum up to 100%.)

a. Misoprostol-only:

__________________________
(%)

b. Mifepristone/misoprostol:

________________________________
(%)

c. Other:

________________________________
(%)

Please specify, other regimen:

________________________________

Total percentage of regimens used for FIRST trimester missed abortions:

________________________________
(%)

Your total does not sum to 100%.

Do you provide any other reproductive health care or general health care during abortion related visits?

◯ Yes
◯ No

Please specify, yes:

________________________________

Please specify, no:

________________________________

Do you provide FIRST trimester medical abortion by telemedicine?

◯ Yes
◯ No

Which component(s) of FIRST trimester medical abortion do you provide via telemedicine? (Check all that apply)

☐ An initial consultation to decide which tests are needed
☐ Consultation to review results and to decide to prescribe mifepristone
☐ Follow-up consultation days or weeks after prescribing mifepristone
☐ Other

Please specify, other:

________________________________

When providing FIRST trimester medical abortion via telemedicine, how often do you obtain or request the following tests on initial assessment? (Please estimate percentages)

a. Ultrasound

________________________________
(%)

b. Urine hCG

________________________________
(%)

c. Quantitative serum βhCG

________________________________
(%)

d. Medical history

______________________________
(%)

e. Rh status

______________________________
(%)

f. Hemoglobin or hematocrit

______________________________
(%)

g. Sexually transmitted infection (STI) testing

______________________________
(%)

h. Other (specify below)

______________________________
(%)

Please specify, other test(s):

______________________________

When providing FIRST trimester medical abortion via telemedicine, how do you usually follow-up to assess if it is complete? (Check all that apply)

- ☐ Ultrasonography
- ☐ Serial serum quantitative βhCG testing
- ☐ Low sensitivity/Semi-quantitative urine hCG testing
- ☐ Standardized evaluation by telephone
- ☐ Standardized evaluation by electronic communication
- ☐ Other

Please specify, other:

______________________________

Which barrier(s) do you perceive to providing telemedicine for FIRST trimester medical abortion in your clinical location? (Check all that apply)

- ☐ Lack of equipment to provide telemedicine
- ☐ Lack of provincial fee code for medication abortion using telemedicine
- ☐ Lack of ability to provide mifepristone/misoprostol
- ☐ Lack of ability to confirm gestational age via ultrasound where patient is located
- ☐ Lack of ability to order serial quantitative serum βhCG
- ☐ Lack of access to urgent D&C and/or blood products close to patient's location
- ☐ Clinic/facility regulations
- ☐ Provincial/territorial regulations
- ☐ None
- ☐ Other

Please specify, other:

______________________________

Have you ever in the past provided surgical abortion? (Check all that apply)

- ☐ First trimester surgical abortion (≤ 13+6 weeks of gestation)
- ☐ Second trimester surgical abortion (≥ 14+0 weeks of gestation)
- ☐ No

| | |
|---|---|
| Are you a registered member of The Canadian Abortion Provider Support/Communauté de pratique canadienne sur l'avortement (CAPS-CPCA)? (Check one) | ◯ Yes<br>◯ No<br>◯ I don't know |
| Do you plan to participate in the CAPS-CPCA forum (online platform)? (Check one)<br><br>CAPS-CPCA website link:<br>https://www.caps-cpca.ubc.ca/ | ◯ Yes<br>◯ No<br>◯ Undecided |
| How many times in the past 6 months have you accessed the CAPS-CPCA website? | ____________________<br>(number of times) |

| | Strongly Disagree | Disagree | Neutral | Agree | Strongly agree |
|---|---|---|---|---|---|
| The CAPS-CPCA website is helpful. (Check one) | ◯ | ◯ | ◯ | ◯ | ◯ |

| | |
|---|---|
| Please specify, strongly disagree: | ____________________ |
| Please specify, disagree: | ____________________ |
| Please specify, neutral: | ____________________ |
| Please specify, agree: | ____________________ |
| Please specify, strongly agree: | ____________________ |
| Is there anything else you would like to share with us in regards to your FIRST trimester medical abortion care, specifically as a nurse practitioner? | ____________________ |
| Is there anything else you would like to share with us in regards to your FIRST trimester medical abortion care? | ____________________ |

# First Trimester Surgical Abortion (FTSA)

Please answer this section of the survey in regards to your practice of induced FIRST trimester surgical abortion care EXCLUDING management of miscarriage.

This section will take you approximately 15 minutes to complete.

Thank you very much for participating in this survey.

---

Time survey opened

______________________________

---

Survey progress (%)

---

You are able to stop the survey at any time by clicking "Save & Return Later" at the bottom of each page. You will be provided with a pop-up of a section-specific randomly generated 8-character return code. To return to the survey, you will need both the return code for the specific section and the survey return link.

We suggest that you retrieve your return code and link now and at the beginning of each subsequent section in case of any technical issues which may result in disconnection from the survey prior to completion.

Click here to download "Save & Return Later" instructions

---

What is the total number of FIRST trimester surgical abortions (defined as surgical abortions ≤ 13+6 weeks of gestational age) you performed in the year 2019? (Please estimate)

______________________________
(number of FIRST trimester surgical abortions in 2019)

---

In how many clinical locations do you provide FIRST trimester surgical abortion care (e.g. 1 hospital and 2 clinics = 3 locations)?

______________________________
(number of clinical locations)

---

Please specify the clinical location of your PRIMARY practice providing FIRST trimester surgical abortion care? (Check one)

- ○ Outside of hospital
- ○ Community hospital
- ○ Academic hospital
- ○ Other

---

Please specify, other:

______________________________

---

Does this PRIMARY clinical location outside of the hospital provide any services other than abortion care? (Check all that apply)

- ☐ Yes, other reproductive health care
- ☐ Yes, general health care other than reproductive health care
- ☐ No, only abortion services

---

What best describes how your PRIMARY hospital-based clinical location provides abortion care? (Check one)

- ○ Provides organized abortion only services/dedicated abortion clinic
- ○ Provides abortion care mixed in with other medical care

---

Does this PRIMARY clinical location provide any services other than abortion care? (Check all that apply)

- ☐ Yes, other reproductive health care
- ☐ Yes, general health care other than reproductive health care
- ☐ No, only abortion services

---

Please specify the clinical location(s) of your ADDITIONAL practice(s) providing FIRST trimester surgical abortion care? (Check all that apply)

- ☐ Outside of hospital
- ☐ Community hospital
- ☐ Academic hospital
- ☐ Other

| | |
|---|---|
| Please specify, other: | __________________________ |
| Does this (do these) ADDITIONAL clinical location(s) outside of the hospital provide any services other than abortion care? (Check all that apply) | ☐ Yes, other reproductive health care<br>☐ Yes, general health care other than reproductive health care<br>☐ No, only abortion services |
| What best describes how your ADDITIONAL hospital-based clinical location(s) provides abortion care? (Check all that apply) | ☐ Provides organized abortion only services/dedicated abortion clinic<br>☐ Provides abortion care mixed in with other medical care |
| Does this (do these) ADDITIONAL other clinical location(s) provide any services other than abortion care? (Check all that apply) | ☐ Yes, other reproductive health care<br>☐ Yes, general health care other than reproductive health care<br>☐ No, only abortion services |
| How many years of experience do you have providing FIRST trimester surgical abortion care after final post-graduate training (e.g. number of years after residency or fellowship)? (Check one) | ◯ < 5 years<br>◯ 5 to 10 years<br>◯ 11 to 15 years<br>◯ 16 to 20 years<br>◯ > 20 years |

**If you provide services in more than one clinical location, please answer the subsequent questions in regards to the clinical location you provide most of your abortion care at. Please answer in regards to the clinical care you provide rather than your clinical location's care unless specified otherwise in the question.**

For which indication(s) do you usually provide FIRST trimester surgical abortion in a live embryo/fetus? (Check all that apply)

- ☐ Patient request without other factors
- ☐ Genetic anomaly not compatible with life
- ☐ Any genetic anomaly
- ☐ Congenital anomaly/malformation not compatible with life
- ☐ Any congenital anomaly
- ☐ Maternal medical indication
- ☐ All of the above
- ☐ Other

Please specify, other: __________________________________

In which case(s) do you usually require a pre-procedure ultrasound before FIRST trimester surgical abortion? (Check all that apply)

- ☐ In all patients
- ☐ In case of unsure last menstrual period (LMP)
- ☐ In case of discrepancy between physical exam and LMP
- ☐ In case of a risk factor for OR a clinical symptom of an ectopic pregnancy
- ☐ In case the pregnancy by LMP or exam is believed to be ≤ 7+0 weeks of gestation
- ☐ In case the pregnancy by LMP or exam is believed to be ≥ 12+0 weeks of gestation
- ☐ Never
- ☐ Other

Please specify, other: __________________________________

Where do you usually access ultrasound for your FIRST trimester surgical abortion patients? (Check all that apply)

- ☐ Through an ultrasound in my clinic
- ☐ Through diagnostic imaging in my health region or hospital/radiology
- ☐ Through maternal-fetal medicine/perinatology in my health region or hospital
- ☐ Other

Please specify, other: __________________________________

Do you experience barriers to obtain an ultrasound for your FIRST trimester surgical abortion?

- ◯ No
- ◯ Yes

Please specify, yes: __________________________________

| | |
|---|---|
| Who usually performs your pre-procedure ultrasound for FIRST trimester surgical abortion? (Check all that apply) | ☐ Trained non-licensed staff<br>☐ Licensed sonogram technician/radiology technician<br>☐ Licensed practical nurse (LPN)<br>☐ Registered nurse (RN)<br>☐ Nurse practitioner (NP)<br>☐ Physician assistant (PA) [not applicable to Quebec]<br>☐ Midwife<br>☐ Physician (non-Radiologist)<br>☐ Physician (Radiologist)<br>☐ Me<br>☐ I don't know<br>☐ Other |
| Please specify, other: | __________________ |
| What is the minimum gestational age/criteria at which you provide FIRST trimester surgical abortion? (Check one) | ◯ As soon as the pregnancy test is positive<br>◯ Starting at a certain gestational age (specify below)<br>◯ If ultrasound is performed, as soon as the intrauterine gestational sac and yolk sac are seen<br>◯ Other |
| Please specify, minimum gestational age (weeks + days) you provide FIRST trimester surgical abortion: | |
| Weeks: | __________________ |
| Days: | __________________ |
| Calculation of: "MINIMUM gestational age/criteria do you offer FIRST trimester surgical abortion" | __________________ |
| Please specify, other: | __________________ |
| What is/are the criteria for which you provide FIRST trimester surgical abortion in the setting of a pregnancy of unknown location (absence of yolk sac or embryo within an intrauterine gestational sac on ultrasound)<br>AND<br>in the absence of a risk factor for or a clinical symptom of an ectopic pregnancy? (Check all that apply) | ☐ No intrauterine gestational sac is seen on ultrasound and the serum βhCG level is $> 2000$ IU/L<br>☐ No intrauterine gestational sac is seen on ultrasound and the serum βhCG level is $\leq 2000$ IU/L<br>☐ A likely intrauterine gestational sac is seen on ultrasound without a yolk sac or fetal pole<br>☐ I do not provide FIRST trimester surgical abortion in the case of pregnancy of unknown location<br>☐ Other |
| Please specify, other: | __________________ |
| Up to what maximum gestational age (weeks + days) do you provide FIRST trimester surgical abortion (defined as surgical abortions $\leq$ 13+6 weeks of gestational age)? | |
| Weeks: | __________________ |

| | |
|---|---|
| Days: | __________________ |
| Calculation of: "MAXIMUM gestational age do you offer FIRST trimester surgical abortion" | __________________ |
| Which factor(s) determines your upper gestational age limit for FIRST trimester surgical abortion? (Check all that apply) | ☐ Definition of the end of the first trimester (≤ 13+6 weeks of gestational age)<br>☐ Personal preference<br>☐ Personal competency<br>☐ Clinic/Facility regulations<br>☐ Provincial/Territorial regulations<br>☐ Other |
| Please specify, other: | __________________ |
| Who usually provides pre-abortion patient education/counselling for FIRST trimester surgical abortion? (Check all that apply) | ☐ Unlicensed trained counsellor/educator/healthcare worker<br>☐ Licensed counsellor/social worker or similarly credentialed person<br>☐ Licensed practical nurse (LPN)<br>☐ Registered nurse (RN)<br>☐ Nurse practitioner (NP)<br>☐ Physician assistant (PA) [not applicable to Quebec]<br>☐ Midwife<br>☐ Physician (attending, resident, or fellow)<br>☐ Me<br>☐ Educational video<br>☐ Other |
| Please specify, other: | __________________ |
| Who primarily discusses pre-abortion informed consent for FIRST trimester surgical abortion? (Check all that apply) | ☐ Unlicensed trained counsellor/educator/healthcare worker<br>☐ Licensed counsellor/social worker or similarly credentialed person<br>☐ Licensed practical nurse (LPN)<br>☐ Registered nurse (RN)<br>☐ Nurse practitioner (NP)<br>☐ Physician assistant (PA) [not applicable to Quebec]<br>☐ Midwife<br>☐ Physician (attending, resident, or fellow)<br>☐ Me<br>☐ Other |
| Please specify, other: | __________________ |
| Do you assess Rh status in the following patients? (Check one) | ◯ In all patients<br>◯ Only if gestational age is ≥ 7+0 weeks<br>◯ Only if gestational age is ≥ 8+0 weeks<br>◯ Never<br>◯ Other |
| Please specify, other: | __________________ |

If a patient is Rh (-), do you offer Rh immune globulin administration? (Check one)

- ◯ To all patients
- ◯ Only if gestational age is ≥ 7+0 weeks
- ◯ Only if gestational age is ≥ 8+0 weeks
- ◯ Never
- ◯ Other

Please specify, other: ______________________________

Which factor(s) do you consider when deciding if and how to prepare the cervix prior to FIRST trimester surgical abortion? (Check all that apply)

- ☐ Gestational age
- ☐ Parity
- ☐ Prior caesarean section
- ☐ Prior vaginal delivery
- ☐ Patient age
- ☐ Prior cervical procedure
- ☐ Fibroid uterus
- ☐ All of the above
- ☐ I do not prepare the cervix
- ☐ Other

Please specify, other: ______________________________

At which gestational age (weeks + days) do you start cervical preparation in patients for FIRST trimester surgical abortion?

Weeks: ______________________________

Days: ______________________________

Calculation for gestational "At which gestational age do you start cervical preparation in patients for FIRST trimester surgical abortion?" ______________________________

**How do you usually prepare the cervix at the following gestational ages? (Check all that apply for each gestational age)**

| | I do not prepare the cervix | Misoprostol alone | Osmotic dilators alone | Both osmotic dilators and misoprostol (together) | Mifepristone | I do not provide FIRST trimester surgical abortion at this gestational age |
|---|---|---|---|---|---|---|
| ≤ 7+6 weeks | ☐ | ☐ | ☐ | ☐ | ☐ | ☐ |
| ≥ 8+0 - ≤ 9+6 weeks | ☐ | ☐ | ☐ | ☐ | ☐ | ☐ |
| ≥ 10+0 - ≤ 11+6 weeks | ☐ | ☐ | ☐ | ☐ | ☐ | ☐ |
| ≥ 12+0 - ≤ 13+6 weeks | ☐ | ☐ | ☐ | ☐ | ☐ | ☐ |

At which gestational age do you usually start cervical preparation in NULLIPAROUS patients for FIRST trimester surgical abortion?

◯ I do not prepare the cervix
◯ Starting at a certain gestational age (specify below)

Please specify, gestational age (weeks + days):

Weeks: ________________________________

Days: ________________________________

Calculation for gestational "which gestational age do you start cervical preparation in NULLIPAROUS patients for FIRST trimester surgical abortion? ________________________________

**How do you usually prepare the cervix in NULLIPAROUS patients at the following gestational ages? (Check all that apply for each gestational age)**

| | I do not prepare the cervix | Misoprostol alone | Osmotic dilators alone | Both osmotic dilators and misoprostol (together) | Mifepristone | I do not provide FIRST trimester surgical abortion at this gestational age |
|---|---|---|---|---|---|---|
| ≤ 7+6 weeks | ☐ | ☐ | ☐ | ☐ | ☐ | ☐ |
| ≥ 8+0 - ≤ 9+6 weeks | ☐ | ☐ | ☐ | ☐ | ☐ | ☐ |
| ≥ 10+0 - ≤ 11+6 weeks | ☐ | ☐ | ☐ | ☐ | ☐ | ☐ |
| ≥ 12+0 - ≤ 13+6 weeks | ☐ | ☐ | ☐ | ☐ | ☐ | ☐ |

At which gestational age do you usually start cervical preparation in MULTIPAROUS patients for FIRST trimester surgical abortion?

○ I do not prepare the cervix
○ Starting at a certain gestational age (specify below)

Please specify, gestational age (weeks + days):

Weeks: __________________________________

Days: __________________________________

Calculation for gestational "which gestational age do you start cervical preparation in MULTIPAROUS patients for FIRST trimester surgical abortion? __________________________________

**How do you usually prepare the cervix in MULTIPAROUS patients at the following gestational ages?**
**(Check all that apply for each gestational age)**

| | I do not prepare the cervix | Misoprostol alone | Osmotic dilators alone | Both osmotic dilators and misoprostol (together) | Mifepristone | I do not provide FIRST trimester surgical abortion at this gestational age |
|---|---|---|---|---|---|---|
| ≤ 7+6 weeks | ☐ | ☐ | ☐ | ☐ | ☐ | ☐ |
| ≥ 8+0 - ≤ 9+6 weeks | ☐ | ☐ | ☐ | ☐ | ☐ | ☐ |
| ≥ 10+0 - ≤ 11+6 weeks | ☐ | ☐ | ☐ | ☐ | ☐ | ☐ |
| ≥ 12+0 - ≤ 13+6 weeks | ☐ | ☐ | ☐ | ☐ | ☐ | ☐ |

Which osmotic dilators do you usually use in your practice for FIRST trimester surgical abortion? (Check one)

◯ Laminaria® only
◯ Dilapan® only
◯ Both types of osmotic dilators

Do you think it is safe to use misoprostol for cervical ripening in patients with a prior uterine scar undergoing a FIRST trimester surgical abortion? (Check one)

◯ Yes
◯ No
◯ Other

Please specify, other:

__________________________________

What initial dose of misoprostol do you usually use for cervical ripening before a FIRST trimester surgical abortion? (Check one)

◯ 200 mcg
◯ 400 mcg
◯ Other

Please specify, other:

__________________________________

What time interval do you usually recommend between the administration of misoprostol for cervical ripening and FIRST trimester surgical abortion?

__________________________________
(hours)

## PROCEDURAL ANALGESIA

Survey progress (%)

Do you use analgesia before or during placement of osmotic dilators for FIRST trimester surgical abortion? (Check one)

- ◯ Yes
- ◯ No
- ◯ Sometimes

Please specify, sometimes:

______________________________

Which of the following analgesic methods do you usually use before or during placement of osmotic dilators before FIRST trimester surgical abortion? (Check all that apply)

- ☐ Non-pharmacological methods (visualization technique, focused breathing, playing music etc.)
- ☐ Local cervical anaesthesia (intracervical block, paracervical block, topical local anaesthesia)
- ☐ Oral nonsteroidal antiflammatory drug (NSAIDs; e.g. Ibuprofen®)
- ☐ Oral opioids
- ☐ Intramuscular analgesia
- ☐ Light sedation (oral anxiolytics)
- ☐ Intravenous moderate (conscious) sedation
- ☐ Deep sedation (purposeful response following repeated or painful stimulation, and protective airway reflexes intact)
- ☐ General anaesthesia (no purposeful response following repeated or painful stimulation with complete loss of protective airway reflexes)
- ☐ Other method(s)

Please specify, other method(s):

______________________________

Which of the following analgesic methods do you usually use for FIRST trimester surgical abortion? (Check all that apply)

- ☐ Non-pharmacological methods (visualization technique, focused breathing, playing music etc.)
- ☐ Local cervical anaesthesia (intracervical block, paracervical block, topical local anaesthesia)
- ☐ Oral nonsteroidal anti-inflammatory drugs (NSAIDs; e.g. Ibuprofen®)
- ☐ Oral opioids
- ☐ Intramuscular analgesia
- ☐ Light sedation (oral anxiolytics)
- ☐ Intravenous moderate (conscious) sedation
- ☐ Deep sedation (purposeful response following repeated painful stimulation, and protective airway reflexes intact)
- ☐ General anaesthesia (no purposeful response following repeated or painful stimulation with complete loss of protective airway reflexes
- ☐ None
- ☐ Other method(s)

Please specify, other method(s):

______________________________

Approximately what percentage of your FIRST trimester surgical abortions is done using the following methods of pain management? (Please estimate percentage for each option. Please be sure that the percentages sum up to 100%)

a. Local cervical anaesthesia only

______________________________
(%)

b. Local cervical anaesthesia plus any oral meds (including NSAIDs) ________________ (%)

c. Local cervical anaesthesia plus any intramuscular meds (± oral meds) ________________ (%)

d. Intravenous moderate (conscious) sedation ± local cervical anaesthesia ________________ (%)

e. Deep sedation (purposeful response following repeated or painful stimulation and protective airway reflexes intact) ________________ (%)

f. General anaesthesia (no purposeful response following repeated or painful stimulation with complete loss of protective airway reflexes) ________________ (%)

Total percentage of pain management methods: ________________ (%)

Your total does not sum up to 100%.

Which medication do you usually use for local cervical anaesthesia? (Check one)
- ◯ Lidocaine/Xylocaine® (± epinephrine)
- ◯ Mepivacaine/Carbocaine®
- ◯ Chloroprocaine
- ◯ Bupivacaine/Marcaine®
- ◯ Bacteriostatic saline
- ◯ Other

Please specify, regarding the medication chosen above, what is the usual dose you give for cervical anaesthesia? (Indicate concentration and amount)

Concentration: ________________

Amount: ________________

Please specify, other local anaesthetic and dose you give for cervical anaesthesia? (Indicate medication (local anaesthetic), concentration and amount)

Medication (local anaesthetic): ________________

Concentration: ________________

Amount: ________________

Where do you inject the majority of the cervical anaesthetic? (Check one)
- ◯ Intracervical
- ◯ Paracervical
- ◯ Other

Please specify, other:

__________________________________

How long do you wait between cervical anaesthesia and cervical dilation? (Check one)

- ◯ I do not wait
- ◯ 1 minute
- ◯ 3 minutes
- ◯ Other

Please specify, other (minutes):

__________________________________
(minutes)

Do you add a buffer (e.g. bicarbonate) to the cervical anaesthetic solution?

- ◯ Yes
- ◯ No

Do you usually add vasopressin to the cervical anaesthetic solution for a FIRST trimester surgical abortion? (Check one)

- ◯ Yes
- ◯ Not vasopressin, but I add another medication (specify below)
- ◯ No

Please specify, other medication:

__________________________________

Who usually administers the medications for intravenous moderate (conscious) sedation in FIRST trimester surgical abortion? (Check one)

- ◯ Registered nurse (RN)
- ◯ Nurse practitioner (NP)
- ◯ Physician assistant (PA) [not applicable to Quebec]
- ◯ Anaesthesiologist (MD)
- ◯ Other non-Anaesthesiologist MD (physician not performing procedure)
- ◯ Physician performing procedure
- ◯ Other

Please specify, other:

__________________________________

What are the medications usually given in your intravenous moderate (conscious) sedation regimen in FIRST trimester surgical abortion? (Check all that apply)

- ☐ Fentanyl
- ☐ Midazolam/Versed®
- ☐ Ketamine
- ☐ Diazepam/Valium®
- ☐ Meperidine/Demerol®
- ☐ Propofol/Diprivan®
- ☐ Nitrous oxide
- ☐ Anti-emetic
- ☐ I don't know
- ☐ Other

Please specify, other:

__________________________________

| | |
|---|---|
| What is your clinic's/facility's nothing by mouth (i.e. NPO) policy for patients receiving intravenous MODERATE (CONSCIOUS) SEDATION in FIRST trimester surgical abortion? (Check all that apply) | ☐ We do not require patients to be NPO<br>☐ No clear liquids for about 2 hours before the procedure<br>☐ No solid food for about 6 to 8 hours before the procedure<br>☐ Nothing to eat or drink after midnight (except sips of water with essential medications)<br>☐ Varies according to clinician preference or other factors<br>☐ I don't know<br>☐ Other |
| Please specify, other: | __________________________________ |
| Do you refer patients who prefer or require either DEEP SEDATION (purposeful response following repeated or painful stimulation, and protective airway reflexes intact) or GENERAL ANAESTHESIA (no purposeful response following repeated or painful stimulation with complete loss of protective airway reflexes) for FIRST trimester surgical abortion to another site? (Check one) | ○ Yes<br>○ No<br>○ I don't know<br>○ Other |
| Please specify, other: | __________________________________ |
| What is your clinic's/facility's nothing by mouth (i.e. NPO) policy for patients receiving DEEP SEDATION or GENERAL ANAESTHESIA in FIRST trimester surgical abortion? (Check all that apply) | ☐ We do not require patients to be NPO<br>☐ No clear liquids for about 2 hours before the procedure<br>☐ No solid food for about 6 to 8 hours before the procedure<br>☐ Nothing to eat or drink after midnight (except sips of water with essential medications)<br>☐ Varies according to clinician preference or other factors<br>☐ I don't know<br>☐ Other |
| Please specify, other: | __________________________________ |
| How are patients receiving GENERAL ANAESTHESIA usually ventilated in FIRST trimester surgical abortion? (Check one) | ○ Bag mask<br>○ Laryngeal mask<br>○ Endotracheal intubation<br>○ I don't know<br>○ Other |
| Please specify, other: | __________________________________ |

| | |
|---|---|
| Do you or your staff usually use any of the following non-pharmacological pain-relieving techniques during a FIRST trimester surgical abortion? (Check all that apply) | ☐ None<br>☐ Visualization technique<br>☐ Focused breathing<br>☐ Massage<br>☐ Biofeedback<br>☐ Self-hypnosis<br>☐ Playing music<br>☐ Aromatherapy<br>☐ Guided Imagery<br>☐ Other |
| Please specify, other: | ______________________________ |

## PROCEDURAL PRACTICES

Do you ever use a manual vacuum aspirator (e.g. Karman®, Ipas®)?

◯ Yes, specify up to which gestational age below (weeks and days of gestation)
◯ No

Please specify, up to which gestational age (weeks + days) you use a manual vacuum aspirator:

Weeks: ______________________

Days: ______________________

Calculation for: "up to which gestational age manual vacuum aspirator is used" ______________________

Do you use a sharp curette (in addition to suction) in your technique of FIRST trimester surgical abortion? (Check one)

◯ Never
◯ Rarely
◯ Sometimes
◯ Often
◯ Always

**When performing a FIRST trimester surgical abortion do you use ultrasound? (Check one for each option)**

| | Routinely | As clinically indicated | Never |
|---|---|---|---|
| Intraoperatively | ◯ | ◯ | ◯ |
| Postoperatively | ◯ | ◯ | ◯ |

When do you perform an immediate postoperative tissue exam for FIRST trimester surgical abortion? (Check one)

◯ Routinely
◯ As clinically indicated
◯ Never

When do you refer a tissue specimen to a pathology lab for FIRST trimester surgical abortion? (Check all that apply)

☐ Routinely as is my preference
☐ Routinely as mandated in all surgical abortion cases (by facility policy or by local/regional/provincial regulation)
☐ As clinically indicated
☐ Other

Please specify, other:

__________________________________

**What is (are) your usual FIRST step(s) to assess if a FIRST trimester surgical abortion is complete? (Check all that apply for each option)**

| | Postoperative ultrasound | Immediate tissue exam | Routinely sending products of conception to pathology | Serial serum quantitative βhCG testing | Other | I do not provide FIRST trimester surgical abortion in this situation |
|---|---|---|---|---|---|---|
| a) Pregnancy of unknown location | ☐ | ☐ | ☐ | ☐ | ☐ | ☐ |
| b) Confirmed intrauterine pregnancy ≤ 7+0 weeks of gestation | ☐ | ☐ | ☐ | ☐ | ☐ | ☐ |
| c) Confirmed intrauterine pregnancy > 7+0 weeks of gestation | ☐ | ☐ | ☐ | ☐ | ☐ | ☐ |

Please specify, other FIRST steps with a pregnancy of unknown location: __________________________________

Please specify, other FIRST steps with a confirmed intrauterine pregnancy ≤ 7+0 weeks of gestation: __________________________________

Please specify, other FIRST steps with a confirmed intrauterine pregnancy > 7+0 weeks of gestation: __________________________________

When do you provide an antibiotic prophylaxis in FIRST trimester surgical abortion? (Check one)

- ◯ Always
- ◯ Only based on risk factors
- ◯ Never
- ◯ Other

Please specify, other: __________________________________

When do you begin the antibiotic prophylaxis in FIRST trimester surgical abortion? (Check one)

a. When osmotic dilators are used

- ◯ I do not use osmotic dilators
- ◯ At the time of cervical osmotic dilator placement
- ◯ Immediately preoperatively (in clinic or hospital on the day of the abortion)
- ◯ Immediately postoperatively (in clinic or hospital on the day of the abortion)
- ◯ The evening before the procedure
- ◯ Other

Please specify, other: __________________________________

When do you begin the antibiotic prophylaxis in FIRST trimester surgical abortion? (Check one)

b. When no osmotic dilators are used

- ◯ Immediately preoperatively (in clinic or hospital on the day of the abortion)
- ◯ Immediately postoperatively (in clinic or hospital on the day of the abortion)
- ◯ The evening before the procedure
- ◯ I always use osmotic dilators
- ◯ Other

Please specify, other: __________________________________

| | |
|---|---|
| Which antibiotic regimen do you usually give for prophylaxis in FIRST trimester surgical abortion? (Check all that apply)<br><br>a. When osmotic dilators are used | ☐ I do not use osmotic dilators<br>☐ Doxycycline single dose<br>☐ Doxycycline multiple doses<br>☐ Azithromycin<br>☐ Metronidazole single dose<br>☐ Metronidazole multiple doses<br>☐ Other |
| Please specify, other: | __________________________________ |
| Which antibiotic regimen do you usually give for prophylaxis in FIRST trimester surgical abortion? (Check all that apply)<br>b. When no osmotic dilators are used | ☐ Doxycycline single dose<br>☐ Doxycycline multiple doses<br>☐ Azithromycin<br>☐ Metronidazole single dose<br>☐ Metronidazole multiple doses<br>☐ I always use osmotic dilators<br>☐ Other |
| Please specify, other: | __________________________________ |
| Which of the following bleeding PROPHYLAXES do you usually provide for FIRST trimester surgical abortion? (Check all that apply) | ☐ Carboprost (Hemabate®)<br>☐ Methylergonovine<br>☐ Foley bulb<br>☐ Misoprostol<br>☐ Oxytocin<br>☐ Tranexamic acid<br>☐ Vasopressin<br>☐ None<br>☐ Other |
| Please specify, other: | __________________________________ |
| Which of the following treatments do you usually provide for excessive bleeding in FIRST trimester surgical abortion? (Check all that apply) | ☐ Carboprost (Hemabate®)<br>☐ Methylergonovine<br>☐ Foley bulb<br>☐ Misoprostol<br>☐ Oxytocin<br>☐ Tranexamic acid<br>☐ Vasopressin<br>☐ All of the above<br>☐ Other |
| Please specify, other: | __________________________________ |
| What route of misoprostol do you usually use for treatment of excessive bleeding in FIRST trimester surgical abortion? (Check one) | ○ Oral (swallowed)<br>○ Buccal (between teeth and cheek)<br>○ Vaginal<br>○ Sublingual (under the tongue)<br>○ Rectal |
| What total initial dose of misoprostol do you usually use for treatment of excessive bleeding in FIRST trimester surgical abortion (may include multiple initial routes of administration, e.g. oral ± rectal)? (Check one) | ○ 200 mcg<br>○ 400 mcg<br>○ 600 mcg<br>○ 800 mcg<br>○ 1000 mcg<br>○ Other |

Please specify, other: __________________________________

## POST PROCEDURE PRACTICES

Do you usually offer scheduling a routine post-abortion visit following FIRST trimester surgical abortion? (Check all that apply)

☐ No
☐ Yes, for all patients (at our facility or with their preferred care provider)
☐ Yes, for all patients having an early FIRST trimester surgical abortion (≤ 7+0 weeks of gestation)
☐ Yes, for all patients having a FIRST trimester surgical abortion in the setting of a pregnancy of unknown location
☐ Yes, for all patients having a late FIRST trimester surgical abortion (≥ 12+0 weeks of gestation)
☐ Yes, for all patients who had an intrauterine system (IUS)/intrauterine device (IUD) inserted at the time of their procedure (at our facility or with their preferred care provider)
☐ Yes, telephone follow-up through our facility
☐ Other

Please specify, other:

__________________________________

What percentage of your patients initiates a long acting reversible contraceptive (LARC)/leaves with a prescription for a short acting reversible contraceptive (SARC)/plans to use another method after a FIRST trimester surgical abortion? (Please estimate percentage for each option. Please be sure that the percentages sum to 100%)

a) LARC: intrauterine system (IUS)/intrauterine device (IUD):

__________________________________
(%)

b) SARC: oral contraceptive pill, ring, patch, injectable contraception (e.g. depo-medroxyprogesterone acetate (DMPA)):

__________________________________
(%)

c) Barrier methods only or other methods:

__________________________________
(%)

d) None:

__________________________________
(%)

Total percentage of contraceptive methods:

__________________________________

Your total does not sum to 100%.

Do you offer immediate (at the end of the surgical procedure) placement of an IUS/IUD? (Check one)

◯ Yes
◯ No
◯ Other

Please specify, other:

__________________________________

What is the average wait time between the patient's first contact (phone call or referral) with your clinical location and them having a FIRST trimester surgical abortion? (Please estimate)

__________________________________
(number of days)

| | |
|---|---|
| Do you travel to provide FIRST trimester surgical abortion (i.e. outside the community where you primarily practice)? | ◯ Yes<br>◯ No |
| How far is the furthest community where you provide FIRST trimester surgical abortion from your home (one way trip)? | __________________<br>(km) |
| When did you receive training for FIRST trimester surgical abortion? (Check one) | ◯ Residency<br>◯ Post-residency |
| Please specify, specialty and name/location of residency program: | __________________<br>(specify residency program) |
| Please specify, name of hospital or university: | __________________<br>(specify hospital or university) |
| Is there anything else you would like to share with us in regards to your FIRST trimester surgical abortion care? | ______________________ |

# Second Trimester Surgical Abortion (STSA)

Please answer this section of the survey on SECOND TRIMESTER surgical abortion in regards to your practice of induced abortion care EXCLUDING management of intrauterine fetal demise (IUFD).

This section will take you approximately 15 minutes to complete.

Thank you very much for participating in this survey.

---

Time survey opened

__________________________________

---

Survey progress (%)

---

You are able to stop the survey at any time by clicking "Save & Return Later" at the bottom of each page. You will be provided with a pop-up of a section-specific randomly generated 8-character return code. To return to the survey, you will need both the return code for the specific section and the survey return link.

We suggest that you retrieve your return code and link now and at the beginning of each subsequent section in case of any technical issues which may result in disconnection from the survey prior to completion.

Click here to download "Save & Return Later" instructions

---

What is the total number of SECOND trimester surgical abortions (defined as surgical abortions ≥ 14+0 weeks of gestational age) you performed in the year 2019? (Please estimate)

__________________________________
(number of SECOND trimester surgical abortions in 2019)

---

In how many clinical locations do you provide SECOND trimester surgical abortion care (e.g. 1 hospital and 2 clinics = 3 locations)?

__________________________________
(number of clinical locations)

---

Please specify the clinical location of your PRIMARY practice providing SECOND trimester surgical abortion care? (Check one)

- ◯ Outside of hospital
- ◯ Community hospital
- ◯ Academic hospital
- ◯ Other

---

Please specify, other:

__________________________________

---

Does this PRIMARY clinical location outside of the hospital provide any services other than abortion care? (Check all that apply)

- ☐ Yes, other reproductive health care
- ☐ Yes, general health care other than reproductive health care
- ☐ No, only abortion services

---

What best describes how your PRIMARY hospital-based clinical location provides abortion care? (Check one)

- ◯ Provides organized abortion only service/dedicated abortion clinic
- ◯ Provides abortion care mixed in with other medical care

---

Does this PRIMARY clinical location provide any services other than abortion care? (Check all that apply)

- ☐ Yes, other reproductive health care
- ☐ Yes, general health care other than reproductive health care
- ☐ No, only abortion services

---

Please specify the clinical location(s) of you ADDITIONAL practice(s) providing SECOND trimester surgical abortion care? (Check all that apply)

- ☐ Outside of hospital
- ☐ Community hospital
- ☐ Academic hospital
- ☐ Other

Please specify, other: ______________________

Does this (do these) ADDITIONAL clinical location(s) outside of the hospital provide any services other than abortion care? (Check all that apply)

- ☐ Yes, other reproductive health care
- ☐ Yes, general health care other than reproductive health care
- ☐ No, only abortion services

What best describes how your ADDITIONAL hospital-based clinical location(s) provides abortion care? (Check all that apply)

- ☐ Provides organized abortion only service/dedicated abortion clinic
- ☐ Provides abortion care mixed in with other medical care

Does this (do these) ADDITIONAL other clinical location(s) provide any services other than abortion care? (Check all that apply)

- ☐ Yes, other reproductive health care
- ☐ Yes, general health care other than reproductive health care
- ☐ No, only abortion services

How many years of experience do you have providing SECOND trimester surgical abortion care after final post-graduate training (e.g. number of years after residency or fellowship)? (Check one)

- ◯ < 5 years
- ◯ 5 to 10 years
- ◯ 11 to 15 years
- ◯ 16 to 20 years
- ◯ > 20 years

Up to what maximum gestational age do you provide surgical abortion at your PRIMARY clinical location? (Please indicate for each option)

a) For patient request without other factors:

Weeks: ______________________

Days: ______________________

Calculation of "maximum gestational age at which you offer SECOND trimester surgical abortion" for patient request: ______________________

b) For maternal medical indications:

Weeks: ______________________

Days: ______________________

Calculation of "maximum gestational age at which you offer SECOND trimester surgical abortion" for maternal medical indications: ______________________

c) For fetal indications:

Weeks: ______________________

Days: __________________________________

Calculation of "maximum gestational age at which you offer SECOND trimester surgical abortion" for fetal indications: __________________________________

Up to what maximum gestational age do you provide surgical abortion at your ADDITIONAL clinical location with the highest gestational age limit? (Please indicate for each option)

a) For patient request without other factors:

Weeks: __________________________________

Days: __________________________________

Calculation of "maximum gestational age at which you offer SECOND trimester surgical abortion" for patient request: __________________________________

b) For maternal medical indications:

Weeks: __________________________________

Days: __________________________________

Calculation of "maximum gestational age at which you offer SECOND trimester surgical abortion" for maternal medical indications: __________________________________

c) For fetal indications:

Weeks: __________________________________

Days: __________________________________

Calculation of "maximum gestational age at which you offer SECOND trimester surgical abortion" for fetal indications: __________________________________

**Which factor(s) determines your upper gestational age limit for SECOND trimester surgical abortion? (Check all that apply for each option)**

| | Personal preference | Personal competency | Clinic/facility regulations | Provincial/ territorial regulations | Other |
|---|---|---|---|---|---|
| At your PRIMARY clinical location | ☐ | ☐ | ☐ | ☐ | ☐ |
| At your ADDITIONAL clinical location with the highest gestational age limit | ☐ | ☐ | ☐ | ☐ | ☐ |

Please specify, other factor(s) at your PRIMARY clinical location: ________________________________

Please specify, other factor(s) at your ADDITIONAL clinical location with the highest gestational age limit: ________________________________

For which indication(s) do you or your PRIMARY clinic/facility usually provide SECOND trimester surgical abortion in a live fetus? (Check all that apply)

☐ Patient request without other factors
☐ Genetic anomaly not compatible with life
☐ Any genetic anomaly
☐ Congenital anomaly/malformation not compatible with life
☐ Any congenital anomaly/malformation
☐ Preterm premature rupture of membranes
☐ Maternal medical indication
☐ All of the above
☐ Other

Please specify, other: ________________________________

For which indication(s) do you or your ADDITIONAL clinic/facility usually provide SECOND trimester surgical abortion in a live fetus? (If you provide at multiple additional clinical locations answer for the one with the highest gestational age limit) (Check all that apply)

☐ Patient request without other factors
☐ Genetic anomaly not compatible with life
☐ Any genetic anomaly
☐ Congenital anomaly/malformation not compatible with life
☐ Any congenital anomaly/malformation
☐ Preterm premature rupture of membranes
☐ Maternal medical indication
☐ All of the above
☐ Other

Please specify, other: ________________________________

**If you provide services in more than one clinical location, please answer the subsequent questions in regards to the clinical location you provide most of your abortion care at. Please answer in regards to the clinical care you provide rather than your clinical location's care unless specified otherwise in the question.**

| | |
|---|---|
| In which case(s) do you usually require a pre-procedure ultrasound before SECOND trimester surgical abortion? (Check all that apply) | ☐ In all patients<br>☐ In case of unsure last menstrual period (LMP)<br>☐ In case of discrepancy between physical exam and LMP<br>☐ Never<br>☐ Other |
| Please specify, other: | ______________________ |
| In which case(s) do you assess placental location with ultrasound before SECOND trimester surgical abortion? (Check all that apply) | ☐ In all patients<br>☐ Starting at a certain gestational age (specify below)<br>☐ Placenta praevia is suspected<br>☐ History of uterine scar<br>☐ Never<br>☐ Other |
| Please specify, gestational age (weeks + days): | |
| Weeks: | ______________________ |
| Days: | ______________________ |
| Calculation of: "gestational age at which you start assessing placental location with ultrasound before SECOND trimester surgical abortion" | ______________________ |
| Please specify, other: | ______________________ |
| When are you concerned about invasive placentation (e.g. placenta accreta) before SECOND trimester surgical abortion? (Check all that apply) | ☐ Ultrasound showing placenta praevia and history of uterine scar<br>☐ Ultrasound showing low lying anterior placenta and history of uterine scar<br>☐ Never<br>☐ Other |
| Please specify, other: | ______________________ |
| Who do you usually consult if invasive placentation (e.g. placenta accreta) is suspected before SECOND trimester surgical abortion? (Check all that apply) | ☐ No one<br>☐ Radiologist<br>☐ Maternal fetal medicine subspecialist/Perinatologist<br>☐ Other |
| Please specify, other: | ______________________ |

Where do you usually access ultrasound for your SECOND trimester surgical abortion patients? (Check all that apply)

- ☐ Through an ultrasound in my clinic
- ☐ Through diagnostic imaging in health region or hospital/radiology
- ☐ Through maternal-fetal medicine/perinatology in my health region or hospital
- ☐ Other

Please specify, other: __________________________________

Do you experience barriers to obtain an ultrasound for your SECOND trimester surgical abortion patients?

- ◯ No
- ◯ Yes

Please specify, yes: __________________________________

Who usually performs your pre-procedure ultrasound for SECOND trimester surgical abortion? (Check all that apply)

- ☐ Trained non-licensed staff
- ☐ Licensed sonogram technician/radiology technician
- ☐ Licensed practical nurse (LPN)
- ☐ Registered nurse (RN)
- ☐ Nurse practitioner (NP)
- ☐ Physician assistant (PA) [not applicable to Quebec]
- ☐ Midwife
- ☐ Physician (non-Radiologist)
- ☐ Physician (Radiologist)
- ☐ Me
- ☐ I don't know
- ☐ Other

Please specify, other: __________________________________

Who usually provides pre-abortion patient education/counselling for SECOND trimester surgical abortion? (Check all that apply)

- ☐ Unlicensed trained counsellor/educator/healthcare worker
- ☐ Licensed counsellor/social worker or similarly credentialed person
- ☐ Licensed practical nurse (LPN)
- ☐ Registered nurse (RN)
- ☐ Nurse practitioner (NP)
- ☐ Physician assistant (PA) [not applicable in Quebec]
- ☐ Midwife
- ☐ Physician (attending, resident, or fellow)
- ☐ Genetic counsellor
- ☐ Me
- ☐ Educational video
- ☐ Other

Please specify, other: __________________________________

| | |
|---|---|
| Who primarily discusses pre-abortion informed consent for SECOND trimester surgical abortion? (Check all that apply) | ☐ Unlicensed trained counsellor/educator/healthcare worker<br>☐ Licensed counsellor/social worker or similarly credentialed person<br>☐ Licensed practical nurse (LPN)<br>☐ Registered nurse (RN)<br>☐ Nurse practitioner (NP)<br>☐ Physician assistant (PA) [not applicable in Quebec]<br>☐ Midwife<br>☐ Physician (attending, resident, or fellow)<br>☐ Me<br>☐ Other |
| Please specify, other: | __________________________ |
| Starting at which gestational age do you or your clinic/facility usually perform a pre-abortion injection to induce fetal demise (e.g. digoxin or KCl) prior to SECOND trimester surgical abortion? (Check one) | ◯ Never performed<br>◯ Starting at a certain gestational age only (specify below) |
| Please specify, gestational age (weeks + days): | |
| Weeks: | __________________________ |
| Days: | __________________________ |
| Calculation of: "gestational age at which you usually perform a pre-abortion injection to induce fetal demise" | __________________________ |

**What is your or your clinic's/facility's recommendation to the patient regarding performing a pre-abortion injection to induce fetal demise prior to SECOND trimester surgical abortion? (Check one for each option)**

| | Always recommended | Optional per patient preference | Never recommended |
|---|---|---|---|
| a. Below the gestational age you indicated in the previous question | ○ | ○ | ○ |
| b. At or beyond the gestational age you indicated in the previous question | ○ | ○ | ○ |

Who usually performs the pre-abortion injection to induce fetal demise prior to SECOND trimester surgical abortion? (Check all that apply)

- ☐ Maternal-fetal medicine subspecialist/Perinatologist
- ☐ Generalist OB/GYN
- ☐ Family physician/General practitioner
- ☐ Other

Please specify, other: __________________________________

**What is the drug most commonly used at your clinic/facility to induce fetal demise before SECOND trimester surgical abortion? (Check one for each option)**

| | Not applicable | Digoxin | Potassium chloride (KCl) | Lidocaine | I don't know | Other |
|---|---|---|---|---|---|---|
| Maternal-fetal medicine subspecialist/Perinatologist | ○ | ○ | ○ | ○ | ○ | ○ |
| Generalist OB/GYN | ○ | ○ | ○ | ○ | ○ | ○ |
| Family physician/General practitioner | ○ | ○ | ○ | ○ | ○ | ○ |
| Other (as specified in the previous question) | ○ | ○ | ○ | ○ | ○ | ○ |

Please specify, other drug commonly used by maternal-fetal medicine subspecialist/Perinatologist: ______________________

Please specify, other drug commonly used by generalist OB/GYN: ______________________

Please specify, other drug commonly used by family physician/general practitioner: ______________________

Please specify, other drug commonly used by other provider: ______________________

Which factor(s) do you consider when deciding if and how to prepare the cervix before SECOND trimester surgical abortion? (Check all that apply)

- ☐ Gestational age
- ☐ Parity
- ☐ Prior caesarean section
- ☐ Prior vaginal delivery
- ☐ Patient age
- ☐ Prior cervical procedure
- ☐ Fibroid uterus
- ☐ All of the above
- ☐ I do not prepare the cervix
- ☐ Other

Please specify, other: ______________________

**How do you usually prepare the cervix at the following gestational ages? (Check all that apply for each gestational age)**

| | I do not prepare the cervix | Misoprostol alone | Osmotic dilators alone | Both osmotic dilators and misoprostol (together) | Mifepristone | I do not provide SECOND trimester surgical abortion at this gestational age |
|---|---|---|---|---|---|---|
| a. ≥ 14+0 - ≤ 15+6 weeks | ☐ | ☐ | ☐ | ☐ | ☐ | ☐ |
| b. ≥ 16+0 - ≤ 17+6 weeks | ☐ | ☐ | ☐ | ☐ | ☐ | ☐ |
| c. ≥ 18+0 - ≤ 19+6 weeks | ☐ | ☐ | ☐ | ☐ | ☐ | ☐ |
| d. ≥ 20+0 weeks | ☐ | ☐ | ☐ | ☐ | ☐ | ☐ |

**How do you usually prepare the cervix in NULLIPAROUS patients at the following gestational ages? (Check all that apply for each gestational age)**

| | I do not prepare the cervix | Misoprostol alone | Osmotic dilators alone | Both osmotic dilators and misoprostol (together) | Mifepristone | I do not provide SECOND trimester surgical abortion at this gestational age |
|---|---|---|---|---|---|---|
| ≥ 14+0 - ≤ 15+6 weeks | ☐ | ☐ | ☐ | ☐ | ☐ | ☐ |
| ≥ 16+0 - ≤ 17+6 weeks | ☐ | ☐ | ☐ | ☐ | ☐ | ☐ |
| ≥ 18+0 - ≤ 19+6 weeks | ☐ | ☐ | ☐ | ☐ | ☐ | ☐ |
| ≥ 20+0 weeks | ☐ | ☐ | ☐ | ☐ | ☐ | ☐ |

**How do you usually prepare the cervix in MULTIPAROUS patients at the following gestational ages?**
**(Check all that apply for each gestational age)**

| | I do not prepare the cervix | Misoprostol alone | Osmotic dilators alone | Both osmotic dilators and misoprostol (together) | Mifepristone | I do not provide SECOND trimester surgical abortion at this gestational age |
|---|---|---|---|---|---|---|
| ≥ 14+0 - ≤ 15+6 weeks | ☐ | ☐ | ☐ | ☐ | ☐ | ☐ |
| ≥ 16+0 - ≤ 17+6 weeks | ☐ | ☐ | ☐ | ☐ | ☐ | ☐ |
| ≥ 18+0 - ≤ 19+6 weeks | ☐ | ☐ | ☐ | ☐ | ☐ | ☐ |
| ≥ 20+0 weeks | ☐ | ☐ | ☐ | ☐ | ☐ | ☐ |

Which osmotic dilators do you usually use in your practice for SECOND trimester surgical abortion? (Check one)

◯ Laminaria® only
◯ Dilapan® only
◯ Both types of osmotic dilators

Do you think it is safe to use misoprostol for cervical ripening in patients with a prior uterine scar undergoing a SECOND trimester surgical abortion? (Check one)

◯ Yes
◯ No
◯ Other

Please specify, other:

__________________________________

What initial dose of misoprostol do you usually use for cervical ripening before SECOND trimester surgical abortion? (Check one)

◯ 200 mcg
◯ 400 mcg
◯ Other

Please specify, other dose:

__________________________________
(mcg)

What time interval do you usually recommend between the administration of misoprostol for cervical ripening and SECOND trimester surgical abortion?

__________________________________
(hours)

## PROCEDURAL ANALGESIA

Survey progress (%)

Do you use analgesia before or during placement of osmotic dilators for SECOND trimester surgical abortion? (Check one)

- ◯ Yes
- ◯ No
- ◯ Sometimes

Please specify, sometimes:

__________________________________

Which of the following analgesic methods do you usually use before or during placement of osmotic dilators before SECOND trimester surgical abortion? (Check all that apply)

- ☐ Non-pharmacological methods (visualization technique, focused breathing, playing music etc.)
- ☐ Local cervical anaesthesia (intracervical block, paracervical block, topical local anaesthesia)
- ☐ Oral nonsteroidal antiflammatory drug (NSAIDs; e.g. Ibuprofen®)
- ☐ Oral opioids
- ☐ Intramuscular analgesia
- ☐ Light sedation (oral anxiolytics)
- ☐ Intravenous moderate (conscious) sedation
- ☐ Deep sedation (purposeful response following repeated or painful stimulation, and protective airway reflexes intact)
- ☐ General anaesthesia (no purposeful response following repeated or painful stimulation with complete loss of protective airway reflexes)
- ☐ Other method(s)

Please specify, other method(s):

__________________________________

Which of the following analgesic methods do you usually use for SECOND trimester surgical abortion? (Check all that apply)

- ☐ Non-pharmacological methods (visualization technique, focused breathing, playing music etc.)
- ☐ Local cervical anaesthesia (intracervical block, paracervical block, topical local anaesthesia)
- ☐ Oral nonsteroidal antiflammatory drugs (NSAIDs; e.g. Ibuprofen®)
- ☐ Oral opioids
- ☐ Intramuscular analgesia
- ☐ Light sedation (oral anxiolytics)
- ☐ Intravenous moderate (conscious) sedation
- ☐ Deep sedation (purposeful response following repeated painful stimulation, and protective airway reflexes intact)
- ☐ General anaesthesia (no purposeful response following repeated or painful stimulation with complete loss of protective airway reflexes)
- ☐ None
- ☐ Other method(s)

Please specify, other method(s):

__________________________________

Approximately what percentage of your SECOND trimester surgical abortions is done using the following methods of pain management? (Please estimate percentage for each gestational age range. Please be sure that the percentages sum up to 100%)

≥ 14+0 - ≤ 15+6 weeks
a. I do not provide surgical abortion at this gestational age (if applicable, enter N/A)

__________________________________

≥ 16+0 - ≤ 17+6 weeks
a. I do not provide surgical abortion at this gestational age (if applicable, enter N/A) ______________________

≥ 18+0 - ≤ 19+6 weeks
a. I do not provide surgical abortion at this gestational age (if applicable, enter N/A) ______________________

≥ 20 weeks
a. I do not provide surgical abortion at this gestational age (if applicable, enter N/A) ______________________

≥ 14+0 - ≤ 15+6 weeks
b. Local cervical anaesthesia only ______________________

≥ 16+0 - ≤ 17+6 weeks
b. Local cervical anaesthesia only ______________________

≥ 18+0 - ≤ 19+6 weeks
b. Local cervical anaesthesia only ______________________

≥ 20 weeks
b. Local cervical anaesthesia only ______________________

≥ 14+0 - ≤ 15+6 weeks
c. Local cervical anaesthesia plus any oral meds (including NSAIDs) ______________________

≥ 16+0 - ≤ 17+6 weeks
c. Local cervical anaesthesia plus any oral meds (including NSAIDs) ______________________

≥ 18+0 - ≤ 19+6 weeks
c. Local cervical anaesthesia plus any oral meds (including NSAIDs) ______________________

≥ 20+0 weeks
c. Local cervical anaesthesia plus any oral meds (including NSAIDs) ______________________

≥ 14+0 - ≤ 15+6 weeks
d. Local cervical anaesthesia plus any intramuscular meds (± oral meds) ______________________

≥ 16+0 - ≤ 17+6 weeks
d. Local cervical anaesthesia plus any intramuscular meds (± oral meds) ______________________

≥ 18+0 - ≤ 19+6 weeks
d. Local cervical anaesthesia plus any intramuscular meds (± oral meds) ______________________

≥ 20+0 weeks
d. Local cervical anaesthesia plus any intramuscular meds (± oral meds) ______________________

≥ 14+0 - ≤ 15+6 weeks
e. Intravenous moderate (conscious) sedation ± local cervical anaesthesia ______________________

≥ 16+0 - ≤ 17+6 weeks
e. Intravenous moderate (conscious) sedation ± local cervical anaesthesia ______________________

≥ 18+0 - ≤ 19+6 weeks
e. Intravenous moderate (conscious) sedation ± local cervical anaesthesia ______________________

≥ 20+0 weeks
e. Intravenous moderate (conscious) sedation ± local cervical anaesthesia ______________________

≥ 14+0 - ≤ 15+6 weeks
f. Deep sedation (purposeful response following repeated or painful stimulation and protective airway reflexes intact) ______________________

≥ 16+0 - ≤ 17+6 weeks
f. Deep sedation (purposeful response following repeated or painful stimulation and protective airway reflexes intact) ______________________

≥ 18+0 - ≤ 19+6 weeks
f. Deep sedation (purposeful response following repeated or painful stimulation and protective airway reflexes intact) ______________________

≥ 20+0 weeks
f. Deep sedation (purposeful response following repeated or painful stimulation and protective airway reflexes intact) ______________________

≥ 14+0 - ≤ 15+6 weeks
g. General anaesthesia (no purposeful response following repeated or painful stimulation with complete loss of protective airways reflexes) ______________________

≥ 16+0 - ≤ 17+6 weeks
g. General anaesthesia (no purposeful response following repeated or painful stimulation with complete loss of protective airways reflexes) ______________________

≥ 18+0 - ≤ 19+6 weeks
g. General anaesthesia (no purposeful response following repeated or painful stimulation with complete loss of protective airways reflexes) ______________________

≥ 20+0 weeks
g. General anaesthesia (no purposeful response following repeated or painful stimulation with complete loss of protective airways reflexes) ______________________

Total percentage of SECOND trimester surgical abortion pain management methods for ≥ 14+0 - ≤ 15+6 weeks: ______________________
(%)

Total percentage of SECOND trimester surgical abortion pain management methods for ≥ 16+0 - ≤ 17+6 weeks: ________________ (%)

Total percentage of SECOND trimester surgical abortion pain management methods for ≥ 18+0 - ≤ 19+6 weeks: ________________ (%)

Total percentage of SECOND trimester surgical abortion pain management methods for ≥ 20 weeks: ________________ (%)

One of your totals does not sum to 100%.

Does your cervical anaesthesia technique differ from your FIRST and SECOND trimester surgical abortion?
- ◯ Yes
- ◯ No

Which medication do you usually use for local cervical anaesthesia? (Check one)
- ◯ Lidocaine/Xylocaine® (± epinephrine)
- ◯ Mepivacaine/Carbocaine®
- ◯ Chloroprocaine
- ◯ Bupivacaine/Marcaine®
- ◯ Bacteriostatic saline
- ◯ Other

Please specify, regarding the medication chosen above, what is the usual dose you give for cervical anaesthesia? (Indicate concentration and amount)

Concentration: ________________

Amount: ________________

Please specify, other local anaesthetic and usual dose you give for cervical anaesthesia? (Indicate medication (local anaesthetic), concentration and amount)

Medication (local anaesthetic): ________________

Concentration: ________________

Amount: ________________

Where do you inject the majority of cervical anaesthetic? (Check one)
- ◯ Intracervical
- ◯ Paracervical
- ◯ Other

Please specify, other: ________________

How long do you wait between cervical anaesthesia and cervical dilation? (Check one)
- ◯ I do not wait
- ◯ 1 minute
- ◯ 3 minutes
- ◯ Other

Please specify, other (minutes):

______________________ (minutes)

Do you add a buffer (e.g. bicarbonate) to the cervical anaesthetic solution?

- ◯ Yes
- ◯ No

Do you usually add vasopressin to the cervical anaesthetic solution in SECOND trimester surgical abortion? (Check one)

- ◯ Yes
- ◯ No
- ◯ Not vasopressin, but I add another medication

Please specify, other medication:

______________________

Who usually administers the medications for intravenous moderate (conscious) sedation in SECOND trimester surgical abortion? (Check one)

- ◯ Registered nurse (RN)
- ◯ Nurse practitioner (NP)
- ◯ Physician assistant (PA) [not applicable to Quebec]
- ◯ Anaesthesiologist (MD)
- ◯ Other non-Anaesthesiologist MD (not physician performing procedure)
- ◯ Physician performing procedure
- ◯ Other

Please specify, other:

______________________

What are the medications usually given in your intravenous moderate (conscious) sedation regimen in SECOND trimester surgical abortion? (Check all that apply)

- ☐ Fentanyl
- ☐ Midazolam/Versed®
- ☐ Ketamine
- ☐ Diazepam/Valium®
- ☐ Meperidine/Demerol®
- ☐ Propofol/Diprivan®
- ☐ Nitrous oxide
- ☐ Anti-emetic
- ☐ I don't know
- ☐ Other

Please specify, other:

______________________

What is your clinic's/facility's nothing by mouth (i.e. NPO) policy for patients receiving intravenous MODERATE (CONSCIOUS) SEDATION in SECOND trimester surgical abortion? (Check all that apply)

- ☐ We do not require patients to be NPO
- ☐ No clear liquids for about 2 hours before the procedure
- ☐ No solid food for about 6 to 8 hours before the procedure
- ☐ Nothing to eat or drink after midnight (except sips of water with essential medications)
- ☐ Varies according to clinician preference or other factors
- ☐ I don't know
- ☐ Other

Please specify, other:

______________________

Do you refer patients who prefer or require either DEEP SEDATION (purposeful response following repeated or painful stimulation, and protective airway reflexes intact) or GENERAL ANAESTHESIA (no purposeful response following repeated or painful stimulation with complete loss of protective airway reflexes) for SECOND trimester surgical abortion to another site? (Check one)

- ◯ Yes
- ◯ No
- ◯ I don't know
- ◯ Other

Please specify, other: ______________________________

What is your clinic's/facility's nothing by mouth (i.e. NPO) policy for patients receiving DEEP SEDATION or GENERAL ANAESTHESIA in SECOND trimester surgical abortion? (Check all that apply)

- ☐ We do not require patients to be NPO
- ☐ No clear liquids for about 2 hours before the procedure
- ☐ No solid food for about 6 to 8 hours before the procedure
- ☐ Nothing to eat or drink after midnight (except sips of water with essential medications)
- ☐ Varies according to clinician preference or other factors
- ☐ I don't know
- ☐ Other

Please specify, other: ______________________________

How are patients receiving GENERAL ANAESTHESIA usually ventilated in SECOND trimester surgical abortion? (Check one)

- ◯ Bag mask
- ◯ Laryngeal mask
- ◯ Endotracheal intubation
- ◯ I don't know
- ◯ Other

Please specify, other: ______________________________

Do you or your staff usually use any of the following non-pharmacological pain-relieving techniques during a SECOND trimester surgical abortion? (Check all that apply)

- ☐ None
- ☐ Visualization technique
- ☐ Focused breathing
- ☐ Massage
- ☐ Biofeedback
- ☐ Self-hypnosis
- ☐ Playing music
- ☐ Aromatherapy
- ☐ Guided imagery
- ☐ Other

Please specify, other: ______________________________

## PROCEDURAL PRACTICES

Do you use a sharp curette (in addition to suction) in your technique of SECOND trimester surgical abortion? (Check one)

- ◯ Never
- ◯ Rarely
- ◯ Sometimes
- ◯ Often
- ◯ Always

**When performing SECOND trimester surgical abortion do you use ultrasound? (Check one for each option)**

| | Routinely | As clinically indicated | Never |
|---|---|---|---|
| Intraoperatively | ◯ | ◯ | ◯ |
| Postoperatively | ◯ | ◯ | ◯ |

When do you perform an immediate postoperative tissue exam for SECOND trimester surgical abortion? (Check one)

- ◯ Routinely
- ◯ As clinically indicated
- ◯ Never

When do you provide an antibiotic prophylaxis in SECOND trimester surgical abortion? (Check one)

- ◯ Always
- ◯ Only based on risk factors
- ◯ Never
- ◯ Other

Please specify, other:

__________________________________

When do you begin the antibiotic prophylaxis in SECOND trimester surgical abortion? (Check one)

a. When osmotic dilators are used

- ◯ I do not use osmotic dilators
- ◯ With cervical osmotic dilator placement
- ◯ Immediately preoperatively (in clinic or hospital on the day of the abortion)
- ◯ Immediately postoperatively (in clinic or hospital on the day of the abortion)
- ◯ The evening before the procedure
- ◯ Other

Please specify, other:

__________________________________

When do you begin the antibiotic prophylaxis in SECOND trimester surgical abortion? (Check one)

b. When no osmotic dilators are used

- ◯ Immediately preoperatively (in clinic or hospital on the day of the abortion)
- ◯ Immediately postoperatively (in clinic or hospital on the day of the abortion)
- ◯ The evening before the procedure
- ◯ I always use osmotic dilators
- ◯ Other

Please specify, other:

__________________________________

Which antibiotic regimen do you usually give for prophylaxis in SECOND trimester surgical abortion? (Check all that apply)

a. When osmotic dilators are used

- ☐ I do not use osmotic dilators
- ☐ Doxycycline single dose
- ☐ Doxycycline multiple doses
- ☐ Azithromycin
- ☐ Metronidazole single dose
- ☐ Metronidazole multiple doses
- ☐ Other

If other, please specify:

__________________________________

| | |
|---|---|
| Which antibiotic regimen do you usually give for prophylaxis in SECOND trimester surgical abortion? (Check all that apply)<br><br>b. When no osmotic dilators are used | ☐ Doxycycline single dose<br>☐ Doxycycline multiple doses<br>☐ Azithromycin<br>☐ Metronidazole single dose<br>☐ Metronidazole multiple doses<br>☐ I always use osmotic dilators<br>☐ Other |
| Please specify, other: | __________________________ |
| Which of the following bleeding PROPHYLAXES do you usually provide for SECOND trimester surgical abortions? (Check all that apply) | ☐ Carboprost (Hemabate®)<br>☐ Methylergonovine<br>☐ Foley bulb<br>☐ Misoprostol<br>☐ Oxytocin<br>☐ Tranexamic acid<br>☐ Vasopressin<br>☐ None<br>☐ Other |
| Please specify, other: | __________________________ |
| Which of the following treatments do you usually provide for excessive bleeding in SECOND trimester surgical abortions? (Check all that apply) | ☐ Carboprost (Hemabate®)<br>☐ Methylergonovine<br>☐ Foley bulb<br>☐ Misoprostol<br>☐ Oxytocin<br>☐ Tranexamic acid<br>☐ Vasopressin<br>☐ All of the above<br>☐ Other |
| Please specify, other: | __________________________ |
| What route of misoprostol do you usually use for treatment of excessive bleeding in SECOND trimester surgical abortion? (Check one) | ◯ Oral (swallowed)<br>◯ Buccal (between teeth and cheek)<br>◯ Vaginal<br>◯ Sublingual (under the tongue)<br>◯ Rectal |

**What total initial dose of misoprostol do you usually use for treatment of excessive bleeding in SECOND trimester surgical abortion (may include multiple initial routes of administration, e.g. oral ± rectal)? (Check best response for each gestational age range)**

| | I do not provide SECOND trimester surgical abortion at this gestational age | 200 mcg | 400 mcg | 600 mcg | 800 mcg | 1000 mcg | Other |
|---|---|---|---|---|---|---|---|
| ≥ 14+0 - ≤ 15+6 weeks | ○ | ○ | ○ | ○ | ○ | ○ | ○ |
| ≥ 16+0 - ≤ 17+6 weeks | ○ | ○ | ○ | ○ | ○ | ○ | ○ |
| ≥ 18+0 - ≤ 19+6 weeks | ○ | ○ | ○ | ○ | ○ | ○ | ○ |
| ≥ 20+0 weeks | ○ | ○ | ○ | ○ | ○ | ○ | ○ |

Please specify, other dose for ≥ 14+0 - ≤ 15+6 weeks: ____________________ (mcg of misoprostol)

Please specify, other dose for ≥ 16+0 - ≤ 17+6 weeks: ____________________ (mcg of misoprostol)

Please specify, other dose for ≥ 18+0 - ≤ 19+6 weeks: ____________________ (mcg of misoprostol)

Please specify, other dose for ≥ 20+0 weeks: ____________________ (mcg of misoprostol)

## POST PROCEDURE PRACTICES

Do you usually offer scheduling a routine post-abortion visit following SECOND trimester surgical abortion? (Check all that apply)

- ☐ No
- ☐ Yes, in person for all patients (at our facility or with their preferred care provider)
- ☐ Yes, in person for all patients who had an intrauterine system (IUS)/intrauterine device (IUD) inserted at the time of their procedure (at our facility or with their preferred care provider)
- ☐ Yes, telephone follow-up through our facility
- ☐ Other

Please specify, other: ______________________

What percentage of your patients initiates a long acting reversible contraceptive (LARC)/leaves with a prescription for a short acting reversible contraceptive (SARC)/plans to use another method after a SECOND trimester surgical abortion? (Please estimate percentage for each option. Please be sure that the percentages sum to 100%.)

a) LARC: intrauterine system (IUS)/intrauterine device (IUD): ______________________ (%)

b) SARC: oral contraceptive pill, ring, patch, injectable contraception (e.g. depo-medroxyprogesterone acetate (DMPA)): ______________________ (%)

c) Barrier methods only or other methods: ______________________ (%)

d)  None: ______________________ (%)

Total percentage of contraceptive methods: ______________________

Your total does not sum to 100%.

Do you offer immediate (at the end of the SECOND trimester surgical procedure) placement of an IUS/IUD? (Check one)

- ◯ Yes
- ◯ No
- ◯ Other

Please specify, other: ______________________

What is the average wait time between the patient's first contact (phone call or referral) with your clinic/facility and them having a SECOND trimester surgical abortion? (Please estimate) ______________________ (number of days)

Do you travel to provide SECOND trimester surgical abortion (i.e. outside the community where you primarily practice)?

- ◯ Yes
- ◯ No

How far is the furthest community where you provide SECOND trimester surgical abortion from your home (one way trip)? ______________________ (km)

| | |
|---|---|
| When did you receive training for SECOND trimester surgical abortion? (Check one) | ◯ Residency<br>◯ Post-residency |
| Please specify, specialty and name/location of residency program: | ______________________________<br>(residency program) |
| Please specify, name of hospital or university: | ______________________________<br>(hospital or university ) |
| Does your site provide SECOND/THIRD trimester medical abortion? (Check one) | ◯ Yes<br>◯ No, we refer patients to another site<br>◯ Not provided and patient not referred to another site<br>◯ Other |
| Please specify, other: | ______________________________ |
| Is there anything else you would like to share with us in regards to your SECOND trimester surgical abortion care? | ______________________________________ |

# Second Trimester And Third Trimester Medical Abortion (Labour Induction) (STMA/TTMA)

Please answer the remainder of this survey in regards to your practice of induced SECOND/THIRD trimester medical abortion (i.e. labour induction) care excluding management of intrauterine fetal death.

This section will take you approximately 10 minutes to complete.

Thank you very much for participating in this survey.

---

Time survey opened

__________________________________

---

Survey progress (%)

---

You are able to stop the survey at any time by clicking "Save & Return Later" at the bottom of each page. You will be provided with a pop-up of a section-specific randomly generated 8-character return code. To return to the survey, you will need both the return code for the specific section and the survey return link.

We suggest that you retrieve your return code and link now and at the beginning of each subsequent section in case of any technical issues which may result in disconnection from the survey prior to completion.

Click here to download "Save & Return Later" instructions

---

What is the total number of SECOND (≥ 14+0 - ≤ 23+6 weeks of gestation) trimester medical abortions (i.e. labour induction) you provided the following components for in the calendar year 2019? (Please estimate for each option)

---

a. Performed a pre-abortion injection to induce fetal demise (e.g. digoxin or KCl)

__________________________________
(number of SECOND trimester medical abortions in 2019)

---

b. Managed labour, but did not deliver the fetus

__________________________________
(number of SECOND trimester medical abortions in 2019)

---

c. Delivered the fetus

__________________________________
(number of SECOND trimester medical abortions in 2019)

---

What is the total number of THIRD (≥ 24+0 weeks of gestation) trimester medical abortions (i.e. labour induction) you provided the following components for in the calendar year 2019? (Please estimate for each option)

---

a. Performed a pre-abortion injection to induce fetal demise (e.g. digoxin or KCl)

__________________________________
(number of THIRD trimester medical abortions in 2019)

---

b. Managed labour, but did not deliver the fetus

__________________________________
(number of THIRD trimester medical abortions in 2019)

c. Delivered the fetus

__________________________________
(number of THIRD trimester medical abortions in 2019)

In how many clinical locations do you provide SECOND/THIRD trimester medical abortion care (e.g. 2 hospitals)?

__________________________________
(number of clinical locations)

Please specify the clinical location of your PRIMARY practice providing SECOND/THIRD trimester medical abortion care? (Check one)

○ Community hospital
○ Academic hospital
○ Ambulatory site on hospital campus
○ Other

Please specify, other:

__________________________________

Please specify the clinical location(s) of your ADDITIONAL practice(s) providing SECOND/THIRD trimester medical abortion care? (Check all that apply)

☐ Community hospital
☐ Academic hospital
☐ Ambulatory site on hospital campus
☐ Other

Please specify, other:

__________________________________

How many years of experience do you have providing SECOND/THIRD trimester medical abortion care after final post-graduate training (e.g. number of years after residency or fellowship)? (Check one)

○ < 5 years
○ 5 to 10 years
○ 11 to 15 years
○ 16 to 20 years
○ > 20 years

**If you provide services in more than one clinical location, please answer the subsequent questions in regards to the clinical location you provide most of your care at. Please answer in regards to the clinical care you provide rather than your clinical location's care unless specified otherwise in the question.**

For which indication(s) do you or your clinic/facility usually provide SECOND trimester (≥ 14+0 - ≤ 23+6 weeks of gestation) medical abortion in a live fetus? (Check all that apply)

- ☐ Patient request without other factors
- ☐ Genetic anomaly not compatible with life
- ☐ Any genetic anomaly
- ☐ Congenital anomaly/malformation not compatible with life
- ☐ Any congenital anomaly/malformation
- ☐ Preterm premature rupture of membranes
- ☐ Maternal medical indication
- ☐ All of the above
- ☐ Other

Please specify, other: ______________________

For which indications do you or your clinic/facility usually provide THIRD trimester (≥ 24+0 weeks of gestation) medical abortion in a live fetus? (Check all that apply)

- ☐ Patient request without other factors
- ☐ Genetic anomaly not compatible with life
- ☐ Any genetic anomaly
- ☐ Congenital anomaly/malformation not compatible with life
- ☐ Any congenital anomaly/malformation
- ☐ Preterm premature rupture of membranes
- ☐ Maternal medical indication
- ☐ All of the above
- ☐ I do not provide abortion ≥ 24+0 weeks of gestation
- ☐ Other

Please specify, other: ______________________

Do you provide SECOND trimester (≥ 14+0 - ≤ 23+6 weeks of gestation) medical abortion in case of a placenta praevia?

- ◯ Yes
- ◯ No

Do you provide THIRD trimester (≥ 24+0 weeks of gestation) medical abortion in case of a placenta praevia? (Check one)

- ◯ Yes
- ◯ No
- ◯ I do not provide THIRD trimester medical abortion

Up to what maximum gestational age do you provide SECOND and/or THIRD trimester medical abortion in case of placenta praevia?

Weeks: ______________________

Days: ______________________

Calculation of: "maximum gestational age do you provide SECOND/THIRD trimester medical abortion in case of placenta praevia" ______________________

| | |
|---|---|
| Which additional service(s) do you usually include in the decision making for a SECOND/THIRD trimester medical abortion? (Check all that apply) | ☐ Ethics consult<br>☐ Social work<br>☐ Specialists involved in the specific care potentially required for the newborn<br>☐ Genetic counsellor<br>☐ Psychiatry<br>☐ Psychology<br>☐ Other<br>☐ None |
| Please specify, ethics consult: | __________________________ |
| Please specify, social work: | __________________________ |
| Please specify, specialists: | __________________________ |
| Please specify, genetic counsellor: | __________________________ |
| Please specify, psychiatry: | __________________________ |
| Please specify, psychology: | __________________________ |
| Please specify, other: | __________________________ |

**Up to what maximum gestational age do you provide SECOND/THIRD trimester medical abortion? (Please check one for each option)**

| | No gestational age limit | Up to a certain gestational age (specify below) | I do not provide abortion for this indication |
|---|---|---|---|
| For patient request without other factors | ○ | ○ | ○ |
| For maternal medical indications | ○ | ○ | ○ |
| For fetal indications | ○ | ○ | ○ |

Please specify, maximum gestational age (weeks + days) for patient request without other factors:

Weeks: ______________________

Days: ______________________

Calculation of: "MAXIMUM gestational age do you offer SECOND/THIRD trimester medical abortion" for patient request without other factors. ______________________

Please specify, maximum gestational age (weeks + days) for maternal medical indications:

Weeks: ______________________

Days: ______________________

Calculation of: "MAXIMUM gestational age do you offer SECOND/THIRD trimester medical abortion" for maternal medical indications. ______________________

Please specify, maximum gestational age (weeks + days) for fetal indications:

Weeks: ______________________

Days: ______________________

Calculation of: "MAXIMUM gestational age do you offer SECOND/THIRD trimester medical abortion" for fetal indications. ______________________

**Which factor(s) determines your upper gestational age limit for SECOND/THIRD trimester medical abortion? (Check all that apply for each option)**

| | Personal preference | Hospital/facility regulations | Provincial/territorial regulations | Other |
|---|---|---|---|---|
| For patient request without other factors | ☐ | ☐ | ☐ | ☐ |
| For maternal medical indications | ☐ | ☐ | ☐ | ☐ |
| For fetal indications | ☐ | ☐ | ☐ | ☐ |

Please specify, other factor(s) for patient request without other factors: __________________________________

Please specify, other factor(s) for maternal medical indications: __________________________________

Please specify, other factor(s) for fetal indications: __________________________________

Who usually delivers patients undergoing a SECOND/THIRD trimester medical abortion in your facility? (Check all that apply)

- ☐ Maternal-fetal medicine subspecialist/Perinatologist
- ☐ Generalist OB/GYN
- ☐ Family physician/General practitioner
- ☐ Other

Please specify, other: __________________________________

Who usually provides pre-abortion patient education/counselling for SECOND/THIRD trimester medical abortion? (Check all that apply)

- ☐ Unlicensed trained counsellor/educator/health care worker
- ☐ Licensed counsellor/social worker or similarly credentialed person
- ☐ Licensed practical nurse (LPN)
- ☐ Registered nurse (RN)
- ☐ Nurse practitioner (NP)
- ☐ Physician assistant (PA) [not applicable to Quebec]
- ☐ Midwife
- ☐ Physician (attending, resident or fellow)
- ☐ Genetic counsellor
- ☐ Me
- ☐ Educational video
- ☐ Other

Please specify, other: __________________________________

Where do you usually access ultrasound for your SECOND/THIRD trimester medical abortion patients? (Check all that apply)

- ☐ Through an ultrasound in my clinic
- ☐ Through diagnostic imaging in my health region or hospital/radiology
- ☐ Through maternal-fetal medicine/perinatology in my health region or hospital
- ☐ Other

Please specify, other: __________________________________

| | |
|---|---|
| Who usually performs ultrasound for your SECOND/THIRD trimester medical abortion patients? (Check all that apply) | ☐ Trained non-licensed staff<br>☐ Licensed sonogram technician/radiology technician<br>☐ Licensed practical nurse (LPN)<br>☐ Registered nurse (RN)<br>☐ Nurse practitioner (NP)<br>☐ Physician assistant (PA) [not applicable to Quebec]<br>☐ Midwife<br>☐ Physician (non-Radiologist)<br>☐ Physician (Radiologist)<br>☐ Me<br>☐ I don't know<br>☐ Other |
| Please specify, other: | __________________________ |
| Survey progress (%) | |
| Starting at which gestational age do you or your clinic/facility usually perform a pre-abortion injection to induce fetal demise (e.g. digoxin or KCl) prior to SECOND/THIRD trimester medical abortion? (Check one) | ◯ Never performed<br>◯ Starting at a certain gestational age (specify below) |
| Please specify, gestational age (weeks + days): | |
| Weeks: | __________________________ |
| Days: | __________________________ |
| Calculation of: "gestational age do you usually perform a pre-abortion injection to induce fetal demise" | __________________________ |

**What is your or your clinic's/facility's recommendation to the patient regarding performing a pre-abortion injection to induce fetal demise prior to SECOND/THIRD trimester medical abortion? (Check one for each option)**

| | Always recommended | Optional per patient preference | Never recommended |
|---|---|---|---|
| a. Below the gestational age you indicated in the previous question | ○ | ○ | ○ |
| b. At or beyond the gestational age you indicated in the previous question | ○ | ○ | ○ |

Who usually performs the pre-abortion injection to induce fetal demise prior to SECOND/THIRD trimester medical abortion? (Check all that apply)

- ☐ Maternal-fetal medicine subspecialist/Perinatologist
- ☐ Generalist OB/GYN
- ☐ Family physician/General practitioner
- ☐ Other

Please specify, other: ________________________________

**What is the drug most commonly used at your clinic/facility to induce fetal demise before SECOND/THIRD trimester medical abortion? (Check one for each option)**

| | Not applicable | Digoxin | Potassium chloride (KCl) | Lidocaine | I don't know | Other |
|---|---|---|---|---|---|---|
| Maternal-fetal medicine subspecialist/Perinatologist | ◯ | ◯ | ◯ | ◯ | ◯ | ◯ |
| Generalist OB/GYN | ◯ | ◯ | ◯ | ◯ | ◯ | ◯ |
| Family physician/General practitioner | ◯ | ◯ | ◯ | ◯ | ◯ | ◯ |
| Other (as specified in the previous question) | ◯ | ◯ | ◯ | ◯ | ◯ | ◯ |

Please specify, other drug commonly used by maternal-fetal medicine subspecialist/perinatologist: ______________________

Please specify, other drug commonly used by generalist OB/GYN: ______________________

Please specify, other drug commonly used by family physician/general practitioner: ______________________

Please specify, other drug commonly used by other provider: ______________________

Which of the following methods do you usually provide for SECOND (≥ 14+0 - ≤ 23+6 weeks of gestation) trimester medical abortion in order to achieve delivery in an unscarred uterus? (Check all that apply)

- ☐ Mifepristone/misoprostol
- ☐ Misoprostol
- ☐ Prostaglandin E-2 (e.g. Cervidil®, Prepidil®)
- ☐ Intravenous oxytocin drip
- ☐ Osmotic dilators
- ☐ Intracervical catheter
- ☐ Intra-amniotic saline or urea
- ☐ Artificial rupture of membranes
- ☐ Other

Please specify, other: ______________________

How often do you use any of the following methods for labour induction in SECOND/THIRD trimester medical abortion in a patient with an unscarred uterus at the following gestational age ranges? (Please estimate percentage for each gestational age range. The sum will likely exceed 100% as you combine options)

≥ 14+0 - ≤ 23+6 weeks
a. Mifepristone/misoprostol ______________________

≥ 24+0 - ≤ 27+6 weeks
a. Mifepristone/misoprostol ______________________

≥ 28+0 - ≤ 31+6 weeks
a. Mifepristone/misoprostol ______________________

≥ 32+0 weeks
a. Mifepristone/misoprostol ______________________

≥ 14+0 - ≤ 23+6 weeks
b. Misoprostol ______________________

≥ 24+0 - ≤ 27+6 weeks
b. Misoprostol ______________________

≥ 28+0 - ≤ 31+6 weeks
b. Misoprostol ______________________

≥ 32+0 weeks
b. Misoprostol ______________________

≥ 14+0 - ≤ 23+6 weeks
c. Prostaglandin E-2 (e.g., Cervidil®, Prepidil®) ______________________

≥ 24+0 - ≤ 27+6 weeks
c. Prostaglandin E-2 (e.g., Cervidil®, Prepidil®) ______________________

≥ 28+0 - ≤ 31+6 weeks
c. Prostaglandin E-2 (e.g., Cervidil®, Prepidil®) ______________________

≥ 32+0 weeks
c. Prostaglandin E-2 (e.g., Cervidil®, Prepidil®) ______________________

≥ 14+0 - ≤ 23+6 weeks
d. Intravenous oxytocin drip ______________________

≥ 24+0 - ≤ 27+6 weeks
d. Intravenous oxytocin drip ______________________

≥ 28+0 - ≤ 31+6 weeks
d. Intravenous oxytocin drip ______________________

≥ 32+0 weeks
d. Intravenous oxytocin drip ______________________

≥ 14+0 - ≤ 23+6 weeks
e. Osmotic dilators ______________________

≥ 24+0 - ≤ 27+6 weeks
e. Osmotic dilators ______________________

≥ 28+0 - ≤ 31+6 weeks
e. Osmotic dilators ______________________

≥ 32+0 weeks
e. Osmotic dilators ______________________

≥ 14+0 - ≤ 23+6 weeks
f. Intracervical catheter ______________________

≥ 24+0 - ≤ 27+6 weeks
f. Intracervical catheter ______________________

≥ 28+0 - ≤ 31+6 weeks
f. Intracervical catheter ______________________

≥ 32+0 weeks
f. Intracervical catheter ______________________

≥ 14+0 - ≤ 23+6 weeks
g. Intra-amniotic saline or urea ______________________

≥ 24+0 - ≤ 27+6 weeks
g. Intra-amniotic saline or urea ______________________

≥ 28+0 - ≤ 31+6 weeks
g. Intra-amniotic saline or urea ______________________

≥ 32+0 weeks
g. Intra-amniotic saline or urea ______________________

≥ 14+0 - ≤ 23+6 weeks
h. Artificial rupture of membranes ______________________

≥ 24+0 - ≤ 27+6 weeks
h. Artificial rupture of membranes ______________________

≥ 28+0 - ≤ 31+6 weeks
h. Artificial rupture of membranes ______________________

≥ 32+0 weeks
h. Artificial rupture of membranes ______________________

≥ 14+0 - ≤ 23+6 weeks
i. Other ______________________

≥ 24+0 - ≤ 27+6 weeks
i. Other ______________________

≥ 28+0 - ≤ 31+6 weeks
i. Other ______________________

≥ 32+0 weeks
i. Other ______________________

Please specify, other regimen: ______________________

In which case(s) do you usually provide a MIFEPRISTONE/MISOPROSTOL regimen for SECOND/THIRD trimester abortion in an unscarred uterus? (Check all that apply)

- ☐ Any SECOND trimester medical abortion ≤ 27+6 weeks of gestation
- ☐ Any THIRD trimester medical abortion ≥ 28+0 weeks of gestation
- ☐ In a SECOND trimester intrauterine fetal demise
- ☐ In a THIRD trimester intrauterine fetal demise
- ☐ Other

Please specify, other: ______________________

Do you experience barriers in providing a MIFEPRISTONE/MISOPROSTOL regimen for SECOND/THIRD trimester medical abortion?

- ◯ Yes
- ◯ No

Please specify, yes: ______________________

| | |
|---|---|
| How long do you usually wait between mifepristone and misoprostol administration? (Check one) | ◯ 24 hours<br>◯ 48 hours<br>◯ Unknown<br>◯ Other |
| Please specify, other: | __________________________ |
| If misoprostol is required immediately to start labour induction for SECOND/THIRD trimester medical abortion in an unscarred uterus, do you give mifepristone at the same time as the first misoprostol dose? | ◯ No, I do not give mifepristone in this case<br>◯ Yes |
| What route of misoprostol administration do you usually recommend for SECOND/THIRD trimester medical abortion? (Check one) | ◯ Oral (swallowed)<br>◯ Buccal (between teeth and cheek)<br>◯ Vaginal<br>◯ Sublingual (under the tongue)<br>◯ Rectal |

**Do you usually use a misoprostol loading dose for SECOND/THIRD trimester medical abortion in an unscarred uterus? (Check one for each option)**

| | Yes, 400 mcg | Yes, 800 mcg | Yes, other dose (specify below) | No |
|---|---|---|---|---|
| In a MIFEPRISTONE/MISOPROSTOL regimen | ○ | ○ | ○ | ○ |
| In a MISOPROSTOL-ONLY regimen | ○ | ○ | ○ | ○ |

Please specify, yes, other dose (mcg) in a MIFEPRISTONE/MISOPROSTOL regimen:

__________________________________
(mcg)

Please specify, yes, other dose (mcg) in a MISOPROSTOL-ONLY regimen:

__________________________________
(mcg)

**Which misoprostol dose (individual dose) do you usually use for SECOND/THIRD trimester medical abortion? (Check one for each gestational age range)**

| | 400 mcg | 200 mcg | 100 mcg | Other | I do not use misoprostol in this case |
|---|---|---|---|---|---|
| Unscarred uterus ≤ 24+6 weeks | ◯ | ◯ | ◯ | ◯ | ◯ |
| Unscarred uterus ≥ 25+0 - ≤ 27+6 weeks | ◯ | ◯ | ◯ | ◯ | ◯ |
| Unscarred uterus ≥ 28+0 weeks | ◯ | ◯ | ◯ | ◯ | ◯ |
| Scarred uterus (one previous low transverse caesarean section) ≤ 24+6 weeks | ◯ | ◯ | ◯ | ◯ | ◯ |
| Scarred uterus (one previous low transverse caesarean section) ≥ 25+0 - ≤ 27+6 weeks | ◯ | ◯ | ◯ | ◯ | ◯ |
| Scarred uterus (one previous low transverse caesarean section) ≥ 28+0 weeks | ◯ | ◯ | ◯ | ◯ | ◯ |

Please specify, other dose for unscarred uterus ≤ 24+6 weeks: ________________ (mcg)

Please specify, other dose for unscarred uterus ≥ 25+0 - ≤ 27+6 weeks: ________________ (mcg)

Please specify, other dose for unscarred uterus ≥ 28+0 weeks: ________________ (mcg)

Please specify, other dose for scarred uterus ≤ 24+6 weeks: ________________ (mcg)

Please specify, other dose for scarred uterus ≥ 25+0 - ≤ 27+6 weeks: ________________ (mcg)

Please specify, other dose for scarred uterus ≥ 28+0 weeks: ________________ (mcg)

Do you differentiate misoprostol use in patients with one low transverse caesarean section versus patients with more than one caesarean section or with another type of uterine scar?

◯ Yes (specify below)
◯ No

Please specify, yes: ________________

**Which misoprostol frequency do you usually use for SECOND/THIRD trimester medical abortion? (Check one for each gestational age range)**

| | Every 3 hours | Every 4 hours | Every 6 hours | Other | I do not use misoprostol in this case |
|---|---|---|---|---|---|
| Unscarred uterus ≤ 24+6 weeks | ○ | ○ | ○ | ○ | ○ |
| Unscarred uterus ≥ 25+0 - ≤ 27+6 weeks | ○ | ○ | ○ | ○ | ○ |
| Unscarred uterus ≥ 28+0 weeks | ○ | ○ | ○ | ○ | ○ |
| Scarred uterus (one previous low transverse caesarean section) ≤ 24+6 weeks | ○ | ○ | ○ | ○ | ○ |
| Scarred uterus (one previous low transverse caesarean section) ≥ 25+0 - ≤ 27+6 weeks | ○ | ○ | ○ | ○ | ○ |
| Scarred uterus (one previous low transverse caesarean section) ≥ 28+0 weeks | ○ | ○ | ○ | ○ | ○ |

Please specify, other frequency for unscarred uterus ≤ 24+6 weeks: ______________________

Please specify, other frequency for unscarred uterus ≥ 25+0 - ≤ 27+6 weeks: ______________________

Please specify, other frequency for unscarred uterus ≥ 28+0 weeks: ______________________

Please specify, other frequency for scarred uterus ≤ 24+6 weeks: ______________________

Please specify, other frequency for scarred uterus ≥ 25+0 - ≤ 27+6 weeks: ______________________

Please specify, other frequency for scarred uterus ≥ 28+0 weeks: ______________________

What method(s) of analgesia or anaesthesia do you usually use during SECOND/THIRD trimester medical abortion? (Check all that apply)

- ☐ Acetaminophen
- ☐ Nonsteroidal anti-inflammatory drugs (NSAIDs; e.g. Ibuprofen®)
- ☐ Oral opioids
- ☐ Intramuscular opioids
- ☐ Intravenous opioids
- ☐ Patient controlled analgesia (PCA) with opioid
- ☐ Regional anaesthesia (Epidural or Spinal)
- ☐ None
- ☐ Other method(s)

Please specify, other method(s): ______________________

**Which uterotonic agent(s) do you usually prophylactically use for THIRD stage of labour management in SECOND/THIRD trimester medical abortion? (Check all that apply for each gestational age range)**

| | Oxytocin | Misoprostol | I do not give a prophylactic uterotonic agent | Other |
|---|---|---|---|---|
| ≥ 14+0 - ≤ 23+6 weeks | ☐ | ☐ | ☐ | ☐ |
| ≥ 24+0 - ≤ 27+6 weeks | ☐ | ☐ | ☐ | ☐ |
| ≥ 28+0 weeks | ☐ | ☐ | ☐ | ☐ |

Please specify, other uterotonic agent for ≥ 14+0 - ≤ 23+6 weeks:

__________________________________
(uterotonic agent)

Please specify, other uterotonic agent for ≥ 24+0 - ≤ 27+6 weeks:

__________________________________
(uterotonic agent)

Please specify, other uterotonic agent for ≥ 28+0 weeks:

__________________________________
(uterotonic agent)

If your patient has a retained placenta (defined as failure of the placenta to expel spontaneously or be extractable by cord traction) but remains asymptomatic, after how many minutes do you usually perform a manual removal and/or curettage? (Check one)

- ◯ 15 minutes
- ◯ 30 minutes
- ◯ 60 minutes
- ◯ 90 minutes
- ◯ 120 minutes
- ◯ 150 minutes
- ◯ 180 minutes
- ◯ No limit

Do you offer the placement of an intrauterine system (IUS)/intrauterine device (IUD) immediately after placental expulsion in the absence of complications? (Check one)

- ◯ Yes
- ◯ No
- ◯ Other

Please specify, other:

__________________________________

Have you ever in the past provided surgical abortion? (Check all that apply)

- ☐ FIRST trimester surgical abortion (≤ 13+6 weeks of gestation)
- ☐ SECOND trimester surgical abortion (≥ 14+0 weeks of gestation)
- ☐ No

**Does your site provide surgical abortion? (Check one for each option)**

| | Yes | No, but we refer patients to another site | Not provided and patient not referred to another site | Other |
|---|---|---|---|---|
| FIRST trimester surgical abortion (≤ 13+6 weeks of gestational age) | ◯ | ◯ | ◯ | ◯ |
| SECOND trimester surgical abortion (≥ 14+0 weeks of gestational age) | ◯ | ◯ | ◯ | ◯ |

Please specify, other for FIRST trimester: ______________________

Please specify, other for SECOND trimester: ______________________

Up to what maximum gestational age (weeks + days) does your site provide surgical abortion?

Weeks: ______________________

Days: ______________________

Calculation of: "MAXIMUM gestational age does your site offer surgical abortion" ______________________

Does your clinic/facility have a protocol for SECOND/THIRD trimester medical abortion care? (Check one)

◯ Yes
◯ No
◯ I don't know

Please specify, yes: ______________________

Would you like to have a protocol for SECOND/THIRD trimester medical abortion care?

◯ Yes
◯ No

Is there anything else you would like to share with us in regards to your SECOND/THIRD trimester medical abortion care? ______________________

# Administrators

Please answer the remainder of this survey in regards to your practice of induced abortion care EXCLUDING management of miscarriage/intrauterine fetal death.

This section will take you approximately 10 minutes to complete.

Thank you very much for participating in this survey.

---

Time survey opened

__________________________________

---

Survey progress (%)

---

You are able to stop the survey at any time by clicking "Save & Return Later" at the bottom of each page. You will be provided with a pop-up of a section-specific randomly generated 8-character return code. To return to the survey, you will need both the return code for the specific section and the survey return link.

We suggest that you retrieve your return code and link now and at the beginning of each subsequent section in case of any technical issues which may result in disconnection from the survey prior to completion.

Click here to download "Save & Return Later" instructions

---

 What type(s) of abortion has your service provided in 2019? (Check all that apply)

- ☐ FIRST trimester medical abortion (≤ 10+0 weeks of gestation or 70 days of gestation)
- ☐ FIRST trimester surgical abortion (≤ 13+6 weeks of gestation)
- ☐ SECOND trimester surgical abortion (≥ 14+0 weeks of gestation)
- ☐ SECOND trimester medical abortion/labour induction (≥ 14+0 - ≤ 23+6 weeks of gestation)
- ☐ THIRD trimester medical abortion/labour induction (≥ 24+0 weeks of gestation)
- ☐ None of the above

---

What best describes your clinical location? (Check one)

- ○ Outside of hospital
- ○ Community hospital
- ○ Academic hospital
- ○ Other

---

Please specify, other:

__________________________________

---

Does this clinical location outside of the hospital provide any services other than abortion care? (Check all that apply)

- ☐ Yes, other reproductive health care
- ☐ Yes, general health care other than reproductive health care
- ☐ No, only abortion services

---

What best describes how your hospital-based clinical location provides abortion care? (Check one)

- ○ Provides organized abortion only service/dedicated abortion clinic
- ○ Provides abortion care mixed in with other medical care

---

Does this location provide any services other than abortion care? (Check all that apply)

- ☐ Yes, other reproductive health care
- ☐ Yes, general health care other than reproductive health care
- ☐ No, only abortion services

| | |
|---|---|
| What is the total number of FIRST trimester medical abortions (defined as medical abortions ≤ 10+0 weeks or 70 days of gestational age) provided in your facility in the calendar year 2019? | ____________________ (number of FIRST trimester medical abortions in 2019) |
| How many of the following health care professionals provide FIRST trimester medical abortion at your facility? (Please give number for each option) | |
| a. Physicians independently prescribing or dispensing a medication for medical abortion as the most responsible provider (MRP): | ____________________ (number of physicians) |
| b. Nurse practitioners (NPs) independently prescribing or dispensing a medication for medical abortion as the most responsible provider (MRP): | ____________________ (number of NPs) |
| c. Nurse practitioners or registered nurses (RNs) providing medical abortion under a WRITTEN agreement (e.g., a directive or a collaborative practice agreement) developed and approved in advance by a group of health professionals identified as authorizers and implementers of the written agreement. This agreement enables implementers to decide to perform ordered procedure(s) under specific conditions, without the direct assessment by the authorizer at the time. | ____________________ (number of NPs and RNs) |
| d. Nurse practitioners or registered nurses providing medical abortion under a VERBAL collaborative/inter-professional care agreement that involves either a midwife, registered nurse or nurse practitioner working with another health care professional who has prescribing authority for medical abortion. | ____________________ (number of NPs and RNs) |
| e. Midwives providing abortion under WRITTEN agreement (e.g., a directive or a collaborative practice agreement) developed and approved in advance by a group of health professionals identified as authorizers and implementers of the written agreement. This agreement enables implementers to decide to perform ordered procedure(s) under specific conditions, without the direct assessment by the authorizer at the time. | ____________________ (number of midwives) |
| f. Midwives providing abortion under a VERBAL collaborative/inter-professional care agreement that involves either a midwife, registered nurse or nurse practitioner working with another health care professional who has prescribing authority for medical abortion. | ____________________ (number of midwives) |
| g. Other health care professional(s): | ____________________ (number of other health care professional(s)) |
| Please specify, the name and other details that describe your WRITTEN agreement with nurse practitioners or registered nurses: | ________________________________ |

| | |
|---|---|
| Please specify, the name and other details that describe your VERBAL agreement with nurse practitioners or registered nurses: | __________________ |
| Please specify, the name and other details that describe your WRITTEN agreement with midwives: | __________________ |
| Please specify, the name and other details that describe your VERBAL agreement with midwives: | __________________ |
| Please specify, other health care professional(s): | __________________ |
| What is the total number of FIRST trimester surgical abortions (defined as surgical abortions ≤ 13+6 weeks of gestational age) your facility performed in the year 2019? | __________________ (number of FIRST trimester surgical abortions in 2019) |
| What is the total number of SECOND trimester surgical abortions (defined as surgical abortions ≥ 14+0 weeks of gestational age) your facility performed in the year 2019? | __________________ (number of SECOND trimester surgical abortions in 2019) |
| What is the total number of SECOND trimester medical abortions/labour induction (defined as ≥ 14+0 - ≤ 23+6 weeks of gestational age) your facility provided in the calendar year 2019? | __________________ (number of SECOND trimester medical abortions in 2019) |
| What is the total number of THIRD trimester medical abortions/labour induction (defined as ≥ 24+0 weeks of gestational age) your facility provided in the calendar year 2019? | __________________ (number of THIRD trimester medical abortions in 2019 ) |
| Which of the following regimens does your facility provide for FIRST trimester medical abortion? (Check all that apply) | ☐ Mifepristone/misoprostol<br>☐ Methotrexate/misoprostol<br>☐ Misoprostol-only for medical abortion (excluding miscarriages)<br>☐ Other |
| Please specify, other: | __________________ |
| Do you assess Rh status in the following patients? (Check one) | ◯ In all patients<br>◯ Only if gestational age is ≥ 7+0 weeks<br>◯ Only if gestational age is ≥ 8+0 weeks<br>◯ Never<br>◯ Other |
| Please specify, other: | __________________ |
| If a patient is Rh (-), do you offer Rh immune globulin administration? (Check one) | ◯ To all patients<br>◯ Only if gestational age is ≥ 7+0 weeks<br>◯ Only if gestational age is ≥ 8+0 weeks<br>◯ Never<br>◯ Other |

Please specify, other: ______________________

Approximately what percentage of FIRST trimester surgical abortions is done using the following methods of pain management? (Please estimate percentage for each option. Please be sure that the percentages sum up to 100%)

a. Local cervical anaesthesia only ______________________ (%)

b. Local cervical anaesthesia plus any oral meds (including nonsteroidal anti-inflammatory drugs (NSAIDs), e.g. Ibuprofen®) ______________________ (%)

c. Local cervical anaesthesia plus any intramuscular meds (± oral meds) ______________________ (%)

d. Intravenous moderate (conscious) sedation ± local cervical anaesthesia ______________________ (%)

e. Deep sedation (purposeful response following repeated or painful stimulation and protective airway reflexes intact) ______________________ (%)

f. General anaesthesia (no purposeful response following repeated or painful stimulation with complete loss of protective airway reflexes) ______________________ (%)

Total percentage of FIRST trimester surgical abortion pain management methods: ______________________ (%)

Your total does not sum up to 100%.

Up to what maximum gestational age (weeks + days) does your facility provide surgical abortion?

Weeks: ______________________

Days: ______________________

Calculation of: "maximum gestational age at which you offer SECOND trimester surgical abortion" ______________________

For which indication(s) does your facility usually provide SECOND trimester surgical abortion in a live fetus? (Check all that apply)

- ☐ Patient request without other factors
- ☐ Genetic anomaly not compatible with life
- ☐ Any genetic anomaly
- ☐ Congenital anomaly/malformation not compatible with life
- ☐ Any congenital anomaly/malformation
- ☐ Preterm premature rupture of membranes
- ☐ Maternal medical indication
- ☐ Other

Please specify, other: __________________________________

Approximately what percentage of SECOND trimester surgical abortions is done using the following methods of pain management? (Please estimate percentages for each gestational age range. Please be sure that the percentages sum to 100%)

≥ 14+0 - ≤ 15+6 weeks
a. Our facility does not provide surgical abortion at this gestational age (in this case enter N/A) __________________________________

≥ 16+0 - ≤ 17+6 weeks
a. Our facility does not provide surgical abortion at this gestational age (in this case enter N/A) __________________________________

≥ 18+0 - ≤ 19+6 weeks
a. Our facility does not provide surgical abortion at this gestational age (in this case enter N/A) __________________________________

≥ 20+0 weeks
a. Our facility does not provide surgical abortion at this gestational age (in this case enter N/A) __________________________________

≥ 14+0 - ≤ 15+6 weeks
b. Local cervical anaesthesia only __________________________________

≥ 16+0 - ≤ 17+6 weeks
b. Local cervical anaesthesia only __________________________________

≥ 18+0 - ≤ 19+6 weeks
b. Local cervical anaesthesia only __________________________________

≥ 20+0 weeks
b. Local cervical anaesthesia only __________________________________

≥ 14+0 - ≤ 15+6 weeks
c. Local cervical anaesthesia plus any oral meds (including nonsteroidal anti-inflammatory drugs (NSAIDs), e.g. Ibuprofen®) __________________________________

≥ 16+0 - ≤ 17+6 weeks
c. Local cervical anaesthesia plus any oral meds (including nonsteroidal anti-inflammatory drugs (NSAIDs), e.g. Ibuprofen®) __________________________________

≥ 18+0 - ≤ 19+6 weeks
c. Local cervical anaesthesia plus any oral meds (including nonsteroidal anti-inflammatory drugs (NSAIDs), e.g. Ibuprofen®) __________________________________

≥ 20+0 weeks
c. Local cervical anaesthesia plus any oral meds (including nonsteroidal anti-inflammatory drugs (NSAIDs), e.g. Ibuprofen®) __________________________________

≥ 14+0 - ≤ 15+6 weeks
d. Local cervical anaesthesia plus any IM meds (± oral meds) __________________________________

| | |
|---|---|
| ≥ 16+0 - ≤ 17+6 weeks<br>d. Local cervical anaesthesia plus any IM meds (± oral meds) | ________________ |
| ≥ 18+0 - ≤ 19+6 weeks<br>d. Local cervical anaesthesia plus any IM meds (± oral meds) | ________________ |
| ≥ 20+0 weeks<br>d. Local cervical anaesthesia plus any IM meds (± oral meds) | ________________ |
| ≥ 14+0 - ≤ 15+6 weeks<br>e. Intravenous moderate (conscious ) sedation ± local cervical anaesthesia | ________________ |
| ≥ 16+0 - ≤ 17+6 weeks<br>e. Intravenous moderate (conscious ) sedation ± local cervical anaesthesia | ________________ |
| ≥ 18+0 - ≤ 19+6 weeks<br>e. Intravenous moderate (conscious ) sedation ± local cervical anaesthesia | ________________ |
| ≥ 20+0 weeks<br>e. Intravenous moderate (conscious ) sedation ± local cervical anaesthesia | ________________ |
| ≥ 14+0 - ≤ 15+6 weeks<br>f. Deep sedation (purposeful response following repeated or painful stimulation and protective airway reflexes intact) | ________________ |
| ≥ 16+0 - ≤ 17+6 weeks<br>f. Deep sedation (purposeful response following repeated or painful stimulation and protective airway reflexes intact) | ________________ |
| ≥ 18+0 - ≤ 19+6 weeks<br>f. Deep sedation (purposeful response following repeated or painful stimulation and protective airway reflexes intact) | ________________ |
| ≥ 20+0 weeks<br>f. Deep sedation (purposeful response following repeated or painful stimulation and protective airway reflexes intact) | ________________ |
| ≥ 14+0 - ≤ 15+6 weeks<br>g. General anaesthesia (no purposeful response following repeated or painful stimulation with complete loss of protective airway reflexes) | ________________ |
| ≥ 16+0 - ≤ 17+6 weeks<br>g. General anaesthesia (no purposeful response following repeated or painful stimulation with complete loss of protective airway reflexes) | ________________ |

≥ 18+0 - ≤ 19+6 weeks
g. General anaesthesia (no purposeful response following repeated or painful stimulation with complete loss of protective airway reflexes) __________________________________

≥ 20+0 weeks
g. General anaesthesia (no purposeful response following repeated or painful stimulation with complete loss of protective airway reflexes) __________________________________

Total percentage of SECOND trimester surgical abortion pain management methods for ≥ 14+0 - ≤ 15+6 weeks: __________________________________ (%)

Total percentage of SECOND trimester surgical abortion pain management methods for ≥ 16+0 - ≤ 17+6 weeks: __________________________________ (%)

Total percentage of SECOND trimester surgical abortion pain management methods for ≥ 18+0 - ≤ 19+6 weeks: __________________________________ (%)

Total percentage of SECOND trimester surgical abortion pain management methods for ≥ 20+0 weeks: __________________________________ (%)

One of your totals does not sum to 100%.

For which indication(s) does your facility usually provide SECOND trimester (≥ 14+0 - ≤ 23+6 weeks of gestation) medical abortion/labour induction in a live fetus? (Check all that apply)

- ☐ Patient request without other factors
- ☐ Genetic anomaly not compatible with life
- ☐ Any genetic anomaly
- ☐ Congenital anomaly/malformation not compatible with life
- ☐ Any congenital anomaly/malformation
- ☐ Preterm premature rupture of membranes
- ☐ Maternal medical indication
- ☐ Other

Please specify, other: __________________________________

For which indication(s) does your facility usually provide THIRD trimester (≥ 24+0 weeks of gestation) medical abortion/labour induction in a live fetus? (Check all that apply)

- ☐ Patient request without other factors
- ☐ Genetic anomaly not compatible with life
- ☐ Any genetic anomaly
- ☐ Congenital anomaly/malformation not compatible with life
- ☐ Any congenital anomaly/malformation
- ☐ Preterm premature rupture of membranes
- ☐ Maternal medical indication
- ☐ Other

Please specify, other: __________________________________

Up to what maximum gestational age does your facility provide SECOND/THIRD trimester medical abortion/labour induction? (Check one)

- ○ No gestational age limit
- ○ Up to a certain gestational age (specify below)

Please specify, maximum gestational age (weeks + days):

Weeks: __________________________________

Days: __________________________________

Calculation of: "maximum gestational age at which you offer SECOND/THIRD trimester medical abortion/labour induction" __________________________________

Who usually delivers patients undergoing a SECOND/THIRD trimester medical abortion/labour induction at your facility? (Check all that apply)

- ☐ Maternal-fetal medicine subspecialist/Perinatologist
- ☐ Generalist OB/GYN
- ☐ Family physician/General practitioner
- ☐ Other

Please specify, other: __________________________________

Which factor(s) determines the upper gestational age limit for abortion at your facility? (Check all that apply)

- ☐ Provider availability
- ☐ Clinic/facility regulations
- ☐ Provincial/territorial regulations
- ☐ Evidence-based guidelines
- ☐ Other

Please specify, other __________________________________

# Diverse Patient Populations

The following questions assess if you adjust your abortion care (e.g. to specific environment, specific personnel or specific training) to diverse patient populations (e.g. cultural/ethnic origins, gender/identity, etc.).

This section will take you approximately 5 minutes to complete.

Thank you very much for participating in this survey.

---

Time survey opened ______________________

---

Survey progress (%)

---

You are able to stop the survey at any time by clicking "Save & Return Later" at the bottom of each page. You will be provided with a pop-up of a section-specific randomly generated 8-character return code. To return to the survey, you will need both the return code for the specific section and the survey return link.

We suggest that you retrieve your return code and link now and at the beginning of each subsequent section in case of any technical issues which may result in disconnection from the survey prior to completion.

Click here to download "Save & Return Later" instructions

---

Do you provide abortion care to diverse patient populations (e.g. cultural/ethnic origins, gender/identity, etc.)? (Check one)

- ◯ Yes
- ◯ No
- ◯ Prefer not to answer

---

Do you adjust your abortion care to diverse patient populations (e.g. cultural/ethnic origins, gender/identity, etc.)? (Check one)

- ◯ All of the time
- ◯ Most of the time
- ◯ Some of the time
- ◯ Never
- ◯ Prefer not to answer

---

Please explain how you adjust your abortion care (e.g. to specific environment, specific personnel or specific training): ______________________

---

Have you ever had specific training for providing abortion care to diverse populations during your education/professional training?

- ◯ Yes
- ◯ No

---

What aspects of your training (abortion specific or general education/professional training) have helped or would help you to provide abortion care to diverse populations? ______________________

---

In the context of abortion provision, which of the following continuing medical education opportunities would be relevant in supporting your care to diverse populations? (Check all that apply)

- ☐ In-person training
- ☐ Web-based training
- ☐ I don't know
- ☐ Other

---

Please explain, other: ______________________

# Stigma and Resilience (Experiences as a Provider or Administrator)

The next set of questions is exploring your experience with stigma and resilience.

This section will take you approximately 5 minutes to complete.

Thank you very much for participating in this survey.

---

Time survey opened ________________________________

---

Survey progress (%)

---

You are able to stop the survey at any time by clicking "Save & Return Later" at the bottom of each page. You will be provided with a pop-up of a section-specific randomly generated 8-character return code. To return to the survey, you will need both the return code for the specific section and the survey return link.

We suggest that you retrieve your return code and link now and at the beginning of each subsequent section in case of any technical issues which may result in disconnection from the survey prior to completion.

Click here to download "Save & Return Later" instructions

**Who is aware of your work as a health care provider providing abortion care? (Check one for each option)**

| | Yes | No | Not applicable | I don't know | Prefer not to answer |
|---|---|---|---|---|---|
| My spouse or partner | ◯ | ◯ | ◯ | ◯ | ◯ |
| At least one of my children | ◯ | ◯ | ◯ | ◯ | ◯ |
| At least one parent | ◯ | ◯ | ◯ | ◯ | ◯ |
| At least one sibling | ◯ | ◯ | ◯ | ◯ | ◯ |
| At least one close friend | ◯ | ◯ | ◯ | ◯ | ◯ |
| At least one neighbour | ◯ | ◯ | ◯ | ◯ | ◯ |
| At least one medical co-worker | ◯ | ◯ | ◯ | ◯ | ◯ |
| Other | ◯ | ◯ | ◯ | ◯ | ◯ |

Please specify, other: ______________________

**Please read the following statements and check the response that most closely represents your experience.**

**Disclosure Management:**

| | All of the time | Often | Sometimes | Rarely | Never |
|---|---|---|---|---|---|
| People's reactions to my being an abortion worker make me keep to myself. | ◯ | ◯ | ◯ | ◯ | ◯ |
| I feel like if I tell people about my work they will only see me as an abortion worker. | ◯ | ◯ | ◯ | ◯ | ◯ |
| I worry about telling people that I work in abortion care. | ◯ | ◯ | ◯ | ◯ | ◯ |
| It bothers me if people in my neighbourhood know that I work in abortion care. | ◯ | ◯ | ◯ | ◯ | ◯ |
| I avoid telling people what I do for a living. | ◯ | ◯ | ◯ | ◯ | ◯ |
| I am afraid that if I tell people I work in abortion care I could put myself or my loved ones at risk for violence. | ◯ | ◯ | ◯ | ◯ | ◯ |
| I feel that disclosing my abortion work is not worth the potential hassle that could result. | ◯ | ◯ | ◯ | ◯ | ◯ |
| I am afraid of how people will react if they find out about my work in abortion care. | ◯ | ◯ | ◯ | ◯ | ◯ |
| I feel the need to hide my work in abortion care from my friends. | ◯ | ◯ | ◯ | ◯ | ◯ |
| I find it hard to tell people I work in abortion care. | ◯ | ◯ | ◯ | ◯ | ◯ |

**Please read the following statements and check the response that most closely represents your experience.**

**Internalized states:**

| | All of the time | Often | Sometimes | Rarely | Never |
|---|---|---|---|---|---|
| I am proud that I work in abortion care. | ○ | ○ | ○ | ○ | ○ |
| I feel connected to others who do this work. | ○ | ○ | ○ | ○ | ○ |
| I feel ashamed of the work that I do. | ○ | ○ | ○ | ○ | ○ |
| By providing abortions I am making a positive contribution to society. | ○ | ○ | ○ | ○ | ○ |
| I question whether or not providing abortion care is a good thing to do. | ○ | ○ | ○ | ○ | ○ |
| I find it important to share with people that I work in abortion care. | ○ | ○ | ○ | ○ | ○ |
| I feel guilty about the work that I do. | ○ | ○ | ○ | ○ | ○ |
| I feel that my work in abortion care is targeted by restrictive legislation more than other types of health care. | ○ | ○ | ○ | ○ | ○ |
| I feel that when I disclose my abortion work to strangers, they are supportive of me. | ○ | ○ | ○ | ○ | ○ |
| I feel good about my work in abortion care. | ○ | ○ | ○ | ○ | ○ |

**Please read the following statements and check the response that most closely represents your experience.**

**Judgement:**

| | All of the time | Often | Sometimes | Rarely | Never |
|---|---|---|---|---|---|
| I feel that other health workers look down on me because of my decision to work in abortion care. | ◯ | ◯ | ◯ | ◯ | ◯ |
| I feel that society (the general public) does not value me as an abortion worker. | ◯ | ◯ | ◯ | ◯ | ◯ |
| When I see or read something about abortion in the papers or television, it makes me feel bad about myself. | ◯ | ◯ | ◯ | ◯ | ◯ |
| I feel other health care workers question my professional skills when they learn that I work in abortion care. | ◯ | ◯ | ◯ | ◯ | ◯ |
| I feel that people question my morals when they learn I work in abortion care. | ◯ | ◯ | ◯ | ◯ | ◯ |
| I feel that friends and family who do not work in abortion care do not understand my work. | ◯ | ◯ | ◯ | ◯ | ◯ |
| I worry that my friends and family will think less of me if I talk about the upsetting or difficult parts of my work in abortion care. | ◯ | ◯ | ◯ | ◯ | ◯ |

**Please read the following statements and check the response that most closely represents your experience.**

**Social isolation:**

| | All of the time | Often | Sometimes | Rarely | Never |
|---|---|---|---|---|---|
| I feel that when I disclose my abortion work to family and friends they are supportive of me. | ○ | ○ | ○ | ○ | ○ |
| I talk openly with my friends about my work in abortion care. | ○ | ○ | ○ | ○ | ○ |
| I talk openly with my family about my work in abortion care. | ○ | ○ | ○ | ○ | ○ |
| I can talk to close friends or family about a hard day at work. | ○ | ○ | ○ | ○ | ○ |

**Please read the following statements and check the response that most closely represents your experience.**

**Discrimination:**

| | All of the time | Often | Sometimes | Rarely | Never |
|---|---|---|---|---|---|
| I have been verbally threatened or attacked as a result of working in abortion care. | ◯ | ◯ | ◯ | ◯ | ◯ |
| I have been physically threatened or attacked as a result of working in abortion care. | ◯ | ◯ | ◯ | ◯ | ◯ |
| My family has been harassed or discriminated against by others who find out about my work in abortion care. | ◯ | ◯ | ◯ | ◯ | ◯ |
| People treat my family members differently if they know about my work in abortion care. | ◯ | ◯ | ◯ | ◯ | ◯ |

As a health care provider providing abortion care, did you personally experience any of the following acts of harassment or violence in 2019? (Check all that apply)

- ☐ Vandalism or unlawful trespass onto your personal property (home, car, etc.)
- ☐ Stalking or videotaping of you or your family member
- ☐ Failed or successful attempts at hacking into your personal computer
- ☐ Picketing in your home neighbourhood
- ☐ Picketing or confrontations at your home, work, children's school or place of worship
- ☐ Been denied a position at/an appointment to a local hospital due to performing abortions
- ☐ Been denied membership in a professional society, religious or civic organization due to performing abortions
- ☐ None of these
- ☐ Prefer not to answer
- ☐ Other acts

Please specify, other acts:

____________________________________

Have you ever had any experience of stigma or harassment that has made you change the abortion care you provide? (Check one)

- ◯ Yes
- ◯ No, I have never experienced stigma or harassment
- ◯ No, I have never experienced this degree of stigma or harassment
- ◯ Prefer not to answer

Please explain, yes:

____________________________________

Have you ever had any experience of stigma or harassment that has made you seriously consider not to provide abortion care anymore? (Check one)

- ◯ Yes
- ◯ No, I have never experienced stigma or harassment
- ◯ No, I have never experienced this degree of stigma or harassment
- ◯ Prefer not to answer

Please explain, yes: ______________________________

**For the years 2018 and 2019, please rate the degree of difficulty/ease that you experienced as an administrator when recruiting for the following staff positions for abortion services? (Check one for each option)**

| | Very easy | Somewhat easy | Neither easy nor difficult | Somewhat difficult | Very difficult | Not applicable |
|---|---|---|---|---|---|---|
| Clinic manager | ○ | ○ | ○ | ○ | ○ | ○ |
| FIRST trimester medical abortion provider (≤ 10+0 weeks or 70 days of gestation) | ○ | ○ | ○ | ○ | ○ | ○ |
| FIRST trimester surgical abortion provider (≤ 13+6 weeks of gestation) | ○ | ○ | ○ | ○ | ○ | ○ |
| SECOND trimester surgical abortion provider (≥ 14+0 weeks of gestation) | ○ | ○ | ○ | ○ | ○ | ○ |
| SECOND trimester medical abortion provider (≥ 14+0 - ≤ 23+6 weeks of gestation) | ○ | ○ | ○ | ○ | ○ | ○ |
| THIRD trimester medical abortion provider (≥ 24+0 weeks of gestation) | ○ | ○ | ○ | ○ | ○ | ○ |
| Anaesthesiologist (MD) | ○ | ○ | ○ | ○ | ○ | ○ |
| Licensed practical nurse (LPN) | ○ | ○ | ○ | ○ | ○ | ○ |
| Registered nurse (RN) | ○ | ○ | ○ | ○ | ○ | ○ |
| Nurse practitioner (NP) | ○ | ○ | ○ | ○ | ○ | ○ |
| Midwife | ○ | ○ | ○ | ○ | ○ | ○ |
| Other office staff | ○ | ○ | ○ | ○ | ○ | ○ |

What are the reasons that it is "very difficult" or "somewhat difficult" to recruit staff members in any of the above categories?

__________________________________

Collectively, what acts of harassment and/or violence did your facility/network of facilities or staff experience in 2019? (Check all that apply)

- ☐ Picketing facility without blocking or contact
- ☐ Picketing facility with blocking or contact
- ☐ Picketing of staff homes without blocking or contact
- ☐ Picketing of staff homes with blocking or contact
- ☐ Vandalism
- ☐ Bomb threat
- ☐ Pictures of patients or staff members posted online
- ☐ None of the above
- ☐ Other acts

Please specify, other acts:

__________________________________

About how many acts of harassment and/or violence did your facility/network of facilities or staff experience in 2019? (Please estimate number of acts of harassment and/or violence)

__________________________________
(number of acts of harassment and/or violence in 2019)

In 2018 and 2019, did you receive resignations by members of the following staff categories for any reasons related to harassment, stigma and/or violence? (Check all that apply)

- ☐ Physicians
- ☐ Other licensed staff including anaesthesiologists (MD), midwives, licensed practical nurses (LPN), registered nurses (RN), nurse practitioners (NP), physician assistants (PA), etc.
- ☐ Non-licensed staff members
- ☐ None
- ☐ Other
- ☐ I don't know

Please specify, other: ______________________

Please specify, I don't know: ______________________

Which of the following reasons were given for these resignations? (Check all that apply)

- ☐ Fear of violence or harassment
- ☐ Actual incidence(s) of violence or harassment
- ☐ Feeling stigmatized/looked down upon
- ☐ Other

Please specify, other: ______________________

**If you provide services in more than one clinical location, please answer the subsequent questions in regards to the clinical location you provide most of your abortion care at.**

Are you providing abortion at a facility that has a "bubble zone"? (Check one)

A bubble zone is a designated area around a location which provides abortion services. Within the bubble zone, certain activities are prohibited, such as abortion-related protest and other activities that may intimidate or interfere with patients and abortion service providers.

◯ Yes
◯ No
◯ I don't know

Are you more likely to provide abortion care at a clinical location that has a "bubble zone"? (Check one)

A bubble zone is a designated area around a location which provides abortion services. Within the bubble zone, certain activities are prohibited, such as abortion-related protest and other activities that may intimidate or interfere with patients and abortion service providers.

◯ Yes
◯ No
◯ I don't know
◯ Other

Please explain, yes: ________________________________

Please explain, no: ________________________________

Please explain, other: ________________________________

Does your clinical location have a "bubble zone"? (Check one)

A bubble zone is a designated area around a location which provides abortion services. Within the bubble zone, certain activities are prohibited, such as abortion-related protest and other activities that may intimidate or interfere with patients and abortion service providers.

◯ Yes
◯ No
◯ I don't know

Has your "bubble zone" facilitated or hindered hiring and/or retaining providers of abortion care? (Check one)

A bubble zone is a designated area around a location which provides abortion services. Within the bubble zone, certain activities are prohibited, such as abortion-related protest and other activities that may intimidate or interfere with patients and abortion service providers.

◯ Facilitated
◯ Hindered
◯ Neither facilitated nor hindered
◯ I don't know
◯ Other

Please explain, other: ________________________________

How does your "bubble zone" impact patient care? (Check one)

A bubble zone is a designated area around a location which provides abortion services. Within the bubble zone, certain activities are prohibited, such as abortion-related protest and other activities that may intimidate or interfere with patients and abortion service providers.

- ◯ Positively
- ◯ No impact
- ◯ Negatively
- ◯ I don't know

Please explain, positively:

__________________________________

Please explain, no impact:

__________________________________

Please explain, negatively:

__________________________________

Do you have general comments relating to stigma, harassment, and/or violence?

__________________________________________

If you were to feel distress after answering this section, please find here some national resources:

CART-GRAC: https://cart-grac.ubc.ca/
Action Canada: http://www.actioncanadashr.ca/, Access Line number: 1-888-642-2725
National Abortion Federation: https://prochoice.org/

**Thank you for taking the time to answer these questions about the abortion care you provided in 2019, prior to COVID-19 restrictions.**

**For the following question, we now ask that you think about 2020, and how COVID-19 has impacted your abortion care.**

What impacts has COVID-19 had on your individual abortion practice and/or access to abortion in your province? ________________________________________

# Pilot

EVALUATION OF CLARITY OF LANGUAGE AND USABILITY

Please answer this section of the survey in regards to your evaluation of the clarity of language and usability of the online survey.

This section will take you approximately 5 minutes to complete.

Thank you very much for participating in this survey.

| | |
|---|---|
| Time survey opened | __________________________________ |
| Survey progress (%) | |
| What device did you complete the survey on? (Check one) | ◯ Desktop or laptop computer<br>◯ Mobile device<br>◯ Other |
| Please explain, other: | __________________________________ |
| What browser did you use? (Check one) | ◯ Safari<br>◯ Explorer<br>◯ Google Chrome<br>◯ Firefox<br>◯ Other |
| Please specify, other: | __________________________________ |
| Overall, how easy did you find the online functionality to complete this survey? (Check one) | ◯ Very easy<br>◯ Easy<br>◯ Neutral<br>◯ Difficult<br>◯ Very difficult |
| Please explain, difficult: | __________________________________________ |
| Please explain, very difficult: | __________________________________________ |
| Overall, what do you think about the length of the survey? (Check one) | ◯ Too long<br>◯ Just right<br>◯ Too short |
| Please explain and indicate which topics you suggest to shorten. | __________________________________________ |
| What did you like MOST about this survey and why? | __________________________________________ |

What did you like LEAST about this survey and how would you change it?

__________________________________________

Do you have any other suggestions or feedback for us?

__________________________________________

# KAP Survey

We are inviting you to take part in a short knowledge, attitudes, and current practice survey about long-acting reversible contraception (e.g. intrauterine devices and contraceptive implants) and medical abortion in primary care (Note: Medical abortion is defined as using mifepristone followed by misoprostol to end an early pregnancy up to 63 days)

It should take no more than 15 minutes to complete.

You will receive a $40 gift voucher upon completion and verification via the email that you provide.

Please read the attached Explanatory Statement in full before deciding whether or not to participate in this research.

[Attachment: "Monash Ethics Explanatory Statement_KAP Survey.pdf"]

| | |
|---|---|
| Please click yes to continue | ◯ Yes<br>◯ No |
| Please select your practitioner group | ◯ General Practitioner<br>◯ Nurse/Nurse practitioner<br>◯ Community pharmacist |

# Knowledge, Attitudes and Practices (KAP) of Primary Care Providers in relation to early medical abortion (EMA) and Long Acting Reversible Contraception (LARCs) - GP

Do you work in General practice?

○ Yes
○ No

## SECTION 1: DEMOGRAPHICS

timestamp ____________________

How many years have you worked in general practice? ____________________

Practice Postcode:

Primary practice post code ______
Secondary practice post code (if applicable) ______

Gender
- ☐ Male or man
- ☐ Female or woman
- ☐ Non-binary
- ☐ My gender identity isn't listed. I identify as: ______
- ☐ Prefer not to answer

Age (years)
- ◯ 18-24
- ◯ 25-29
- ◯ 30-34
- ◯ 35-39
- ◯ 40-44
- ◯ 45-49
- ◯ 50-54
- ◯ 55-59
- ◯ 60-64
- ◯ 65+

Please select the qualification(s) relevant to you:
- ☐ GP Registrar
- ☐ FRACGP
- ☐ FACRRM
- ☐ Other ______

What is your primary place of work?
- ◯ General practice
- ◯ Family Planning Organisation
- ◯ Refugee health
- ◯ Marie Stopes Australia
- ◯ Women's health service
- ◯ Other ______

Which of the following best describes the type of general practice in which you work?
- ◯ Small business
- ◯ Corporate chain
- ◯ Other ______

What is your secondary place of work?
- ◯ General practice
- ◯ Family Planning Organisation
- ◯ Refugee health
- ◯ Marie Stopes Australia
- ◯ Women's health service
- ◯ Other ______
- ◯ NA

Which of the following best describes the type of general practice in which you work?
- ◯ Small business
- ◯ Corporate chain
- ◯ Other ______

| | |
|---|---|
| What is your secondary place of work? | ◯ General practice<br>◯ Family Planning Organisation<br>◯ Refugee Health<br>◯ Marie Stopes Australia<br>◯ Women's health service<br>◯ Other<br>◯ NA |
| Which of the following best describes the type of general practice in which you work? | ◯ Small business<br>◯ Corporate chain<br>◯ Other ______ |
| Do you conduct your consultations in a language other than English? | ◯ Yes ______<br>◯ No |

## SECTION 2: PRACTICES

Please answer the below questions about intrauterine devices and contraceptive implants in your practice:

Intrauterine devices Contraceptive implants
Do you insert or remove? ______ ______
How many do you insert in a typical month?

(Please enter '0' if you do not insert)

______ ______
Have you received specific training in inserting or removing intrauterine devices or implants? ______

______

______

______

Where do your patients typically have their insertions? Please tick all that apply. ______ ______

| | |
|---|---|
| How often would you initiate discussions about long-acting reversible contraceptives (e.g. intrauterine devices and contraceptive implants) in your contraceptive consultations? | ◯ Never<br>◯ Rarely<br>◯ Sometimes<br>◯ Very often<br>◯ Always |
| What impact has COVID-19 had on your long-acting reversible contraception service provision? | ______________________________ |
| Do you currently provide termination of pregnancy services? | ◯ Yes, medical abortion<br>◯ Yes, medical abortion and surgical abortion<br>◯ Yes, surgical termination abortion<br>◯ No |
| Do you have any colleagues to whom you can refer for medical abortion if required? | ☐ Yes, within my practice<br>☐ Yes, external to my practice<br>☐ No |
| Are you a registered prescriber of medical abortion medicine (MS-2 Step)? | ◯ Yes<br>◯ No |
| How many years of experience do you have providing medical abortion? (years) | ______________________________ |
| Approximately how many medical abortions do you provide in a typical month? | ______________________________ |
| Do you offer medical abortion services via telemedicine? | ◯ Yes<br>◯ No |
| After counselling for medical abortion, does your practice also discuss intrauterine device insertion? | ☐ Yes, by a practice nurse<br>☐ Yes, by another GP<br>☐ Yes, by myself<br>☐ No |

| | |
|---|---|
| After counselling for medical abortion, does your practice also discuss contraceptive implant insertion? | ☐ Yes, by a practice nurse<br>☐ Yes, by another GP<br>☐ Yes, by myself<br>☐ No |
| Who is aware of your work as a medical abortion provider? (please tick all that apply) | ☐ A local pharmacist<br>☐ A local GP<br>☐ Other local health professionals<br>☐ My practice manager/receptionist<br>☐ Other GPs in my practice<br>☐ Practice nurses in my practice<br>☐ The local radiology practice(s)<br>☐ The local pathology provider(s)<br>☐ A local gynaecologist<br>☐ My local emergency department<br>☐ Other ______ |

## SECTION 3: KNOWLEDGE

**Please indicate your agreement with the following statements:**

| | True | False | Unsure |
|---|---|---|---|
| Long-acting reversible contraceptives are less effective than the contraceptive pill at preventing pregnancy | ○ | ○ | ○ |
| Intrauterine devices' are suitable for use in nulliparous women | ○ | ○ | ○ |
| GP's views and advice can influence the type of contraception selected by patients | ○ | ○ | ○ |
| Fertility can return rapidly after long-acting reversible contraceptive removal | ○ | ○ | ○ |
| In Australia, medical abortion is registered for use up to 9 weeks (63 days) gestation in all states | ○ | ○ | ○ |
| Efficacy of medical abortion is similar to that of surgical abortion | ○ | ○ | ○ |
| Misoprostol is administered before mifepristone | ○ | ○ | ○ |
| Medical abortion medicines can be self-administered at home | ○ | ○ | ○ |

What are the most common side effects from medical abortion? Please tick all that apply.

- ☐ Bleeding
- ☐ Cramping
- ☐ Nausea/vomiting
- ☐ Thrombocytopenia
- ☐ Fever/chills
- ☐ All of the above
- ☐ Don't know

## SECTION 4: ATTITUDES

**Please indicate your agreement with the following statements:**

| | Agree | Disagree | Neither |
|---|---|---|---|
| I have the knowledge to counsel women about the process of medical abortion | ◯ | ◯ | ◯ |
| I feel confident to provide medical abortion medication | ◯ | ◯ | ◯ |
| It is acceptable for GPs to provide medical abortion medication | ◯ | ◯ | ◯ |
| I think women need to know more about the availability of medical abortion | ◯ | ◯ | ◯ |

Do you think the possible side effects of intrauterine devices outweigh the benefits?

- ◯ Yes
- ◯ Unsure
- ◯ No

Do you think the possible side effects of contraceptive implants outweigh the benefits?

- ◯ Yes
- ◯ Unsure
- ◯ No

What factors influence you to recommend long-acting reversible contraceptives to an eligible patient? Please tick all that apply.

- ☐ Age
- ☐ Cost
- ☐ Patient's BMI
- ☐ Past history of abnormal cytology
- ☐ Past history of sexually transmitted infections
- ☐ Marital status
- ☐ History of full-term pregnancies
- ☐ History of abortion
- ☐ Clinician preference
- ☐ Patient preference
- ☐ I don't recommend long-acting reversible contraceptives
- ☐ Other ______

What would dissuade you from providing a medical abortion to an eligible patient? Please tick all that apply.

- ☐ I don't feel I have the knowledge to provide a medical abortion
- ☐ I don't feel I have the skills to provide a medical abortion
- ☐ Cost to patient
- ☐ Cost to the clinic
- ☐ Concerns about safety
- ☐ Concerns about efficacy
- ☐ I am afraid of the stigma associated with providing medical abortion
- ☐ Lack of referral options to specialists/hospitals if a complication occurs
- ☐ Previous negative experience supporting a patient undergoing medical abortion
- ☐ I am a conscientious objector to termination of pregnancy
- ☐ Other ______
- ☐ None/NA

What are the benefits of providing medical abortion in general practice? Please tick all that apply.

- ☐ It is a cost-effective option for women
- ☐ Opportunity to provide more comprehensive care
- ☐ Offers continuity of care for women
- ☐ Reduces women's need to travel to access an abortion service
- ☐ Increased confidentiality for the woman
- ☐ Contraceptive care can be discussed or delivered at the same time
- ☐ I can't see any benefits
- ☐ Other ______

## SECTION 5: EXPERIENCES

**Please indicate which option most closely reflects your experience**

| | Strongly disagree | Disagree | Neither | Agree | Strongly agree | NA |
|---|---|---|---|---|---|---|
| The media take a balanced view on medical abortion | ○ | ○ | ○ | ○ | ○ | ○ |
| I feel discriminated against by other healthcare professionals because of my decision to provide medical abortion services | ○ | ○ | ○ | ○ | ○ | ○ |
| I am proud that I work in abortion care | ○ | ○ | ○ | ○ | ○ | ○ |
| I feel connected to others who do this work | ○ | ○ | ○ | ○ | ○ | ○ |
| I feel that society appreciates the work I do in abortion care | ○ | ○ | ○ | ○ | ○ | ○ |
| I am afraid that if I tell people I work in abortion care I could put myself or my loved ones at risk of harassment/violence | ○ | ○ | ○ | ○ | ○ | ○ |
| I have never experienced harassment and/or violence as a result of working in abortion care | ○ | ○ | ○ | ○ | ○ | ○ |
| I did not realise I had concerns with prescribing medical abortions until I became accredited | ○ | ○ | ○ | ○ | ○ | ○ |
| I feel confident to prescribe medical abortion medicine. | ○ | ○ | ○ | ○ | ○ | ○ |

# Knowledge, Attitudes and Practices (KAP) of Primary Care Providers in relation to early medical abortion (EMA) and Long Acting Reversible Contraception (LARCs) -PN

Do you work in general practice?
- ◯ Yes
- ◯ No

## SECTION 1: DEMOGRAPHICS

timstamp

__________________________________

How many years have you worked in general practice?

__________________________________

Practice Postcode:

Primary practice post code ______
Secondary practice post code (if applicable) ______

Gender

- ☐ Male or man
- ☐ Female or woman
- ☐ Non-binary
- ☐ My gender identity isn't listed. I identify as: ______
- ☐ Prefer not to answer

Age (years)

- ◯ 18-24
- ◯ 25-29
- ◯ 30-34
- ◯ 35-39
- ◯ 40-44
- ◯ 45-49
- ◯ 50-54
- ◯ 55-59
- ◯ 60-64
- ◯ 65+

Please select the qualification(s) relevant to you:

- ☐ Enrolled Nurse
- ☐ Registered Nurse
- ☐ Registered Nurse (advanced practice)
- ☐ Nurse Practitioner
- ☐ Overseas trained
- ☐ Other ______

What is your primary place of work?

- ◯ General practice
- ◯ Family Planning Organisation
- ◯ Refugee health
- ◯ Marie Stopes Australia
- ◯ Women's health service
- ◯ Other ______

Which of the following best describes the type of general practice in which you work?

- ◯ Small business
- ◯ Corporate chain
- ◯ Other ______

What is your secondary place of work?

- ◯ General practice
- ◯ Family Planning Organisation
- ◯ Refugee health
- ◯ Marie Stopes Australia
- ◯ Women's health service
- ◯ Other ______
- ◯ NA

Which of the following best describes the type of general practice in which you work?

- ◯ Small business
- ◯ Corporate chain
- ◯ Other ______

| | |
|---|---|
| What is your secondary place of work? | ◯ General practice<br>◯ Family Planning Organisation<br>◯ Refugee health<br>◯ Marie Stopes Australia<br>◯ Women's health service<br>◯ Other<br>◯ NA |
| Which of the following best describes the type of general practice in which your work? | ◯ Small business<br>◯ Corporate chain<br>◯ Other ______ |
| Do you conduct your consultations in a language other than English? | ◯ Yes ______<br>◯ No |

## SECTION 2: PRACTICES

Please answer the below questions about intrauterine devices and contraceptive implants in your practice:

Intrauterine devices Contraceptive implants
Do you insert or remove? ______ ______
How many do you insert in a typical month?

(Please enter '0' if you do not insert)

______ ______
Have you received specific training in inserting or removing Intrauterine devices or implants? ______

______

______

______

Where do your patients typically have their insertions? Please tick all that apply. ______ ______

| | |
|---|---|
| How often would you initiate discussions about long-acting reversible contraceptives (e.g. intrauterine devices and contraceptive implants) in your contraceptive consultations? | ◯ Never<br>◯ Rarely<br>◯ Sometimes<br>◯ Very often<br>◯ Always |
| What impact has COVID-19 had on your long-acting reversible contraception service provision? | __________________________________ |
| Does your practice currently provide termination of pregnancy services? | ◯ Yes, medical abortion<br>◯ Yes, medical abortion and surgical abortion<br>◯ Yes, surgical termination abortion<br>◯ No |
| Do you have any colleagues to whom you can refer for medical abortion if required? | ◯ Yes<br>◯ No |
| What involvement do nurses/ nurse practitioners have in medical abortion services within your practice? | ◯ Nurse/nurse practitioner-led/managed<br>◯ Some nurse/nurse practitioner involvement (please indicate this involvement) ______<br>◯ No nurse involvement |
| Does your practice offer medical abortion services via telemedicine? | ◯ Yes<br>◯ No |
| After counseling for medical abortion, does your practice also discuss intrauterine device insertion? | ☐ Yes, by another nurse/nurse practitioner<br>☐ Yes, by a GP<br>☐ Yes, by myself<br>☐ No |
| After counseling for medical abortion, does your practice also discuss contraceptive implant insertion? | ☐ Yes, by another nurse/nurse practitioner<br>☐ Yes, a GP<br>☐ Yes, by myself<br>☐ No |

Who is aware of your work in medical abortion services? Please tick all that apply.

- ☐ A local pharmacist
- ☐ A local GP
- ☐ Other local health professionals
- ☐ My practice manager/receptionist
- ☐ GPs in my practice
- ☐ Other nurses in my practice
- ☐ The local radiology practice(s)
- ☐ The local pathology provider(s)
- ☐ A local gynaecologist
- ☐ My local emergency department
- ☐ Other ______

## SECTION 3: KNOWLEDGE

**Please indicate your agreement with the following statements:**

| | True | False | Unsure |
|---|---|---|---|
| Long-acting reversible contraceptives are less effective than the contraceptive pill at preventing pregnancy | ○ | ○ | ○ |
| Intrauterine devices' are suitable for use in nulliparous women | ○ | ○ | ○ |
| Nurse/nurse practitioner's views and advice can influence the type of contraception selected by patients | ○ | ○ | ○ |
| Fertility can return rapidly after long-acting reversible contraceptive removal | ○ | ○ | ○ |
| In Australia, medical abortion is registered for use up to 9 weeks (63 days) gestation in all states | ○ | ○ | ○ |
| Efficacy of medical abortion is similar to that of surgical abortion | ○ | ○ | ○ |
| Misoprostol is administered before mifepristone | ○ | ○ | ○ |
| Medical abortion medicines can be self-administered at home | ○ | ○ | ○ |

What are the most common side effects from medical abortion? Please tick all that apply.

- ☐ Bleeding
- ☐ Cramping
- ☐ Nausea/vomiting
- ☐ Thrombocytopenia
- ☐ Fever/chills
- ☐ All of the above
- ☐ Don't know

## SECTION 4: ATTITUDES

**Please indicate your agreement with the following statements:**

| | Agree | Disagree | Neither |
|---|---|---|---|
| I have the knowledge to counsel women about the process of medical abortion | ○ | ○ | ○ |
| I feel confident in managing medical abortions | ○ | ○ | ○ |
| I feel confident to assist in medical abortion provision | ○ | ○ | ○ |
| It is acceptable for nurse/nurse practitioners to assist with medical abortion | ○ | ○ | ○ |
| I think women need to know more about the availability of medical abortion | ○ | ○ | ○ |

Do you think the possible side effects of intrauterine devices outweigh the benefits?

- ○ Yes
- ○ Unsure
- ○ No

Do you think the possible side effects of contraceptive implants outweigh the benefits?

- ○ Yes
- ○ Unsure
- ○ No

What factors influence you to recommend long-acting reversible contraceptives to an eligible patient? Please tick all that apply.

- ☐ Age
- ☐ Cost
- ☐ Patient's BMI
- ☐ Past history of abnormal cytology
- ☐ Past history of sexually transmitted infections
- ☐ Marital status
- ☐ History of full-term pregnancies
- ☐ History of abortions
- ☐ Clinician preference
- ☐ I don't recommend long-acting reversible contraceptives
- ☐ Other ______

| | |
|---|---|
| What would dissuade you from recommending medical abortion to an eligible patient? Please tick all that apply. | ☐ I don't feel I have the knowledge to assist in the provision of a medical abortion<br>☐ I don't feel I have the skills to assist in the provision of a medical abortion<br>☐ Cost to patient<br>☐ Cost to the clinic<br>☐ Concerns about safety<br>☐ Concerns about efficacy<br>☐ I am afraid of the stigma associated with providing medical abortion<br>☐ Lack of referral options to specialists/hospitals if a complication occurs<br>☐ Previous negative experience supporting a patient undergoing medical abortion<br>☐ I am a conscientious objector to termination of pregnancy<br>☐ Other ______<br>☐ None/NA |
| What are the benefits of providing medical abortion in general practice? Please tick all that apply. | ☐ It is a cost-effective option for women<br>☐ Opportunity to provide more comprehensive care<br>☐ Offers continuity of care for women<br>☐ Reduces women's need to travel to access an abortion service<br>☐ Increased confidentiality for the woman<br>☐ Contraceptive care can be discussed or delivered at the same time<br>☐ I can't see any benefits<br>☐ Other ______ |

## SECTION 5: EXPERIENCES

**Please indicate which option most closely reflects your experience**

| | Strongly disagree | Disagree | Neither | Agree | Strongly agree | NA |
|---|---|---|---|---|---|---|
| The media take a balanced view on medical abortion | ○ | ○ | ○ | ○ | ○ | ○ |
| I feel discriminated against by other healthcare professionals because of my decision to assist in medical abortion services | ○ | ○ | ○ | ○ | ○ | ○ |
| I am proud that I work in abortion care | ○ | ○ | ○ | ○ | ○ | ○ |
| I feel connected to others who do this work | ○ | ○ | ○ | ○ | ○ | ○ |
| I feel that society appreciates the work I do in abortion care | ○ | ○ | ○ | ○ | ○ | ○ |
| I am afraid that if I tell people I work in abortion care I could put myself or my loved ones at risk of harassment/violence | ○ | ○ | ○ | ○ | ○ | ○ |
| I have never experienced harassment and/or violence as a result of working in abortion care | ○ | ○ | ○ | ○ | ○ | ○ |
| 8. My concerns with dispensing before becoming accredited were not realised once I became accredited | ○ | ○ | ○ | ○ | ○ | ○ |
| I feel confident to assist with medical abortions. | ○ | ○ | ○ | ○ | ○ | ○ |

# Knowledge, Attitudes and Practices (KAP) of Primary Care Providers in relation to Medical Termination of Pregnancy (MTOP) and Long Acting Reversible Contraception (LARCs) - Pharmacist

Do you work in primary care?

◯ Yes
◯ No

## SECTION 1: DEMOGRAPHICS

timestamp ____________________

How many years have you worked in primary care? ____________________

Practice Postcode:

Primary pharmacy post code ______
Secondary pharmacy post code (if applicable) ______

Gender
- ☐ Male or man
- ☐ Female or woman
- ☐ Non-binary
- ☐ My gender identity isn't listed. I identify as: ______
- ☐ Prefer not to answer

Age (years)
- ◯ 18-24
- ◯ 25-29
- ◯ 30-34
- ◯ 35-39
- ◯ 40-44
- ◯ 45-49
- ◯ 50-54
- ◯ 55-59
- ◯ 60-64
- ◯ 65+

Please select the qualification(s) relevant to you:
- ☐ BPharm
- ☐ MPharm
- ☐ Overseas Training
- ☐ Other ______

What is your role in the pharmacy? Please tick all that apply
- ☐ Intern pharmacist
- ☐ Pharmacist
- ☐ Pharmacist in charge
- ☐ Pharmacy manager
- ☐ Pharmacy owner
- ☐ Locum pharmacist
- ☐ General practice pharmacist
- ☐ Other ______

Which of the following best describes the type of community pharmacy in which you work?
- ◯ Independent pharmacy
- ◯ Banner group
- ◯ Friendly society
- ◯ Other ______

Do you conduct your consultations in a language other than English?
- ◯ Yes ______
- ◯ No

## SECTION 2: PRACTICES

Please answer the below questions about Intrauterine devices and contraceptive implants in your pharmacy:

| | Intrauterine devices | Contraceptive implants |
|---|---|---|
| Do you dispense? | ______ | ______ |
| How many do you dispense in a typical month?<br><br>(Please enter '0' if you do not dispense) | ______ | ______ |
| Where do your patients typically have their insertions? Please tick all that apply. | ______ | ______ |

How often would you initiate discussions about long-acting reversible contraceptives (e.g. intrauterine devices and contraceptive implants) in your contraceptive consultations?

- ◯ Never
- ◯ Rarely
- ◯ Sometimes
- ◯ Very often
- ◯ Always

What impact has COVID-19 had on your long-acting reversible contraception service provision?

__________________________________

Are you an accredited dispenser of medical abortion medicines (MS-2 Step)?

- ◯ Yes
- ◯ No

How many years of experience do you have providing medical abortion? (years)

__________________________________

Approximately how many medical abortions do you provide in a typical month?

__________________________________

Who is aware of your work as a medical abortion provider? Please tick all that apply.

- ☐ A local pharmacist
- ☐ A local GP
- ☐ Other local health professionals
- ☐ My practice manager/receptionist
- ☐ Other GPs in my practice
- ☐ Practice nurses in my practice
- ☐ The local radiology practice(s)
- ☐ The local pathology provider(s)
- ☐ A local gynaecologist
- ☐ My local emergency department
- ☐ Other ______

In the pharmacy that you work, are there other pharmacists accredited to dispense medical abortion medicines (MS-2 Step)?

- ◯ Yes (please specify how many) ______
- ◯ No

Do you have colleagues who you can refer to dispense medical abortion medicines (MS-2 Step)?

- ◯ Yes
- ◯ No

Do you have anyone to whom you can refer for medical abortion medicines if required?

- ☐ Private gynaecologist
- ☐ Public hospital
- ☐ Family planning clinic
- ☐ A GP
- ☐ Private abortion service
- ☐ Unknown/not sure

| | |
|---|---|
| When you are dispensing medical abortion medicines (MS-2 Step), do you discuss long-acting reversible contraceptive options with the patient? | ☐ Yes, IUDs<br>☐ Yes, implants<br>☐ Yes, IUDs and implants<br>☐ Neither |

## SECTION 3: KNOWLEDGE

**Please indicate your agreement with the following statements:**

| | True | False | Unsure |
|---|---|---|---|
| Long-acting reversible contraceptives are less effective than the contraceptive pill at preventing pregnancy | ◯ | ◯ | ◯ |
| Intrauterine devices' are suitable for use in nulliparous women | ◯ | ◯ | ◯ |
| Pharmacists' views and advice can influence the type of contraception selected by patients | ◯ | ◯ | ◯ |
| Fertility can return rapidly after long-acting reversible contraceptive removal | ◯ | ◯ | ◯ |
| In Australia, medical abortion is registered for use up to 9 weeks (63 days) gestation in all states | ◯ | ◯ | ◯ |
| Efficacy of medical abortion is similar to that of surgical abortion | ◯ | ◯ | ◯ |
| Misoprostol is administered before mifepristone | ◯ | ◯ | ◯ |
| Medical abortion medicines can be self-administered at home | ◯ | ◯ | ◯ |

What are the most common side effects from medical abortion? Please tick all that apply.

- ☐ Bleeding
- ☐ Cramping
- ☐ Nausea/vomiting
- ☐ Thrombocytopenia
- ☐ Fever/chills
- ☐ All of the above
- ☐ Don't know

## SECTION 4: ATTITUDES

**Please indicate your agreement with the following statements:**

| | Agree | Disagree | Neither |
|---|---|---|---|
| I have the knowledge to counsel women about the process of medical abortion | ◯ | ◯ | ◯ |
| I feel confident to dispense medical abortion medications | ◯ | ◯ | ◯ |
| It is acceptable for pharmacists to dispense medical abortion medications | ◯ | ◯ | ◯ |
| I think women need to know more about the availability of medical abortion | ◯ | ◯ | ◯ |

Do you think the possible side effects of intrauterine devices outweigh the benefits?

- ◯ Yes
- ◯ Unsure
- ◯ No

Do you think the possible side effects of contraceptive implants outweigh the benefits?

- ◯ Yes
- ◯ Unsure
- ◯ No

What factors influence you to recommend long-acting reversible contraceptives to an eligible patient? Please tick all that apply.

- ☐ Age
- ☐ Cost
- ☐ Patient's BMI
- ☐ Past history of abnormal cytology
- ☐ Past history of sexually transmitted infections
- ☐ Marital status
- ☐ History of full-term pregnancies
- ☐ History of abortion
- ☐ I don't not recommend long-acting reversible contraceptives
- ☐ Other ______

What would dissuade you from dispensing/counselling on medical abortion to an eligible patient? Please tick all that apply.

- ☐ I don't feel I have the knowledge to recommend MS-2 Step
- ☐ I don't feel I have the skills to recommend MS-2 Step
- ☐ Cost to patient
- ☐ Concerns about safety
- ☐ Concerns about efficacy
- ☐ I am afraid of the stigma associated with dispensing medical abortion
- ☐ Lack of referral options to specialists/hospitals if a complication occurs
- ☐ Previous negative experience supporting a patient undergoing medical abortion
- ☐ I am a conscientious objector to termination of pregnancy
- ☐ Lack of confidential space
- ☐ Lack of time
- ☐ None
- ☐ Other ______

What are the benefits of dispensing medical abortion medicines in a pharmacy? Please tick all that apply.

- ☐ It is a cost-effective option for women
- ☐ Opportunity to provide more comprehensive care
- ☐ Offers continuity of care for women
- ☐ Reduces women's need to travel to access an abortion services
- ☐ Increased confidentiality for the woman
- ☐ Contraceptive care can be delivered at the same time
- ☐ I can't see any benefit
- ☐ Other ______

**SECTION 5: EXPERIENCES**

**Please indicate which option most closely reflects your experience**

| | Strongly disagree | Disagree | Neither | Agree | Strongly agree | NA |
|---|---|---|---|---|---|---|
| Media take a balanced view on medical abortion | ○ | ○ | ○ | ○ | ○ | ○ |
| I feel discriminated against by other healthcare professionals because of my decision to provide medical abortion services | ○ | ○ | ○ | ○ | ○ | ○ |
| I am proud that I work in abortion care | ○ | ○ | ○ | ○ | ○ | ○ |
| I feel connected to others who do this work | ○ | ○ | ○ | ○ | ○ | ○ |
| I feel that society appreciates the work I do in abortion care | ○ | ○ | ○ | ○ | ○ | ○ |
| I am afraid that if I tell people I work in abortion care I could put myself or my loved ones at risk of harassment/violence | ○ | ○ | ○ | ○ | ○ | ○ |
| I have never experienced harassment and/or violence as a result of working in abortion care | ○ | ○ | ○ | ○ | ○ | ○ |
| I did not realise my concerns with dispensing before becoming accredited were not realised once I became accredited | ○ | ○ | ○ | ○ | ○ | ○ |
| I feel confident to dispense medical abortion medicine | ○ | ○ | ○ | ○ | ○ | ○ |

## Thank you for completing the survey.

Please provide your full name as shown on the health practitioner register:

This will be used for verification and will be kept confidential

__________________________________

Please provide your practice postcode as shown on the health practitioner register:

This will be used for verification and will be kept confidential

__________________________________

Please provide your email address for reimbursement purposes:

Within one week of completing the survey, you will be sent your $40 gift voucher to the email you have provided. If you do not receive it please check your junk mail and/or get in touch with us via email AusCAPPS.trial@monash.edu

This will be kept confidential

__________________________________

Please tell us how you found out about the survey?

- ◯ Mail
- ◯ Organisation newsletter/email
- ◯ Friend/colleague
- ◯ Social media
- ◯ Conference
- ◯ Radio/podcast
- ◯ Other

AusCAPPS Network (The Australian Contraception and Abortion Primary Care Practitioner Support Network) is an NHMRC-funded project within the Centre of Research Excellence in Women's Sexual and Reproductive Health (SPHERE). It is designed to connect the primary care workforce and increase women's access to contraception and abortion.
AusCAPPS Network aims to:

- Increase women's access to long-acting reversible contraceptive methods (IUDs and implants).
- Increase women's access to safe, affordable medical abortion, including for women from the most vulnerable populations. JOIN NOW AusCAPPS Network is hosted by Medcast. When joining the AusCAPPS network you may be required to register to Medcast before registering into the AusCAPPS Network.

______

This initiative is funded by the NHMRC in partnership with APNA, DoH, FPTas, FPWNT, FPVic, PSA, RACGP, RANZCOG, SHINE SA, SHQ, Jean Hailes, ASHHNA, Bayer, and MAS.

Thank you again for your time and we hope you consider joining the AusCAPPs Network. Please click submit to complete the survey.

# Join AusCAPPS

## Thank you for your interest. Unfortunately, the survey has now closed.

Thank you for your interest. Unfortunately, the survey has now closed.

You might be interested in the newly launched AusCAPPS (The Australian Contraception and Abortion Primary Care Practitioner Support) Network.
This free online network aims to connect and support GPs, nurses and community pharmacists in the provision of long-acting reversible contraception and medical abortion.

Through the network you will be able to:

- Connect with peers
- Access our national network of providers
- Access our national resource library
- Discuss case studies and chat with experts
- Find training opportunities to become a provider
- Read the latest news and research

JOIN NOW

AusCAPPS Network is hosted by Medcast. When joining the AusCAPPS network you may be required to register to Medcast before registering into the AusCAPPS Network.

# EXTEND PREFER: Eligibility

Thank you for your interest in our research. First, please answer some simple questions to find out whether this study is suitable for you to participate in.

1. How old are you? (years)

○ Less than 16 years

○ 16 to 25 years

○ Greater than 25 years

Please record today's date (DD/MM/YYYY)

________________________________________________________________

2. Have you been sexually active with a male partner in the past 6 months or do you anticipate sexual activity in the next 6 months?

○ Yes

○ No

3. Are you pregnant?

○ Yes

○ No

4. Do you hope to become pregnant in the next year?

○ Yes

○ No

5. Have you had a tubal ligation or hysterectomy?
   Tubal ligation is a surgical procedure in which a woman's fallopian tubes are clipped, cut, tied or sealed shut to prevent pregnancy.
   A hysterectomy is an operation which involves the removal of the uterus (womb).

○ Yes

○ No

6. Has your partner had a vasectomy?
   A vasectomy is an operation that involves cutting and blocking the tubes that carry sperm from the testicles.
   (if you do not have a regular sexual partner, select 'no')

○ Yes

○ No

7. Do you identify with any of the following backgrounds?

○ Chinese

○ Indian

○ Middle-Eastern

○ None of the above

Thank you for answering our questions.
Your responses to these questions indicate that you are eligible to take part in our study! You will now be redirected to the next survey.

8. Do you live in a rural or remote area?

○ Yes

○ No

9. Please provide your postcode

________________________________________

# Survey one (pre-video)

Please answer these questions as openly and honestly as you can. There are no right or wrong answers. Some of the questions are very personal but we would like to remind you that all your responses will remain confidential.

This survey is made up of 5 sections:

Your contact detailsAbout youSex and relationshipsContraceptive experienceCurrent contraceptive preferencesIt should take you about 5-10 minutes to complete this survey.

After the survey you will be able to watch our contraceptive education video (13 minutes). You will then be asked to complete survey two (5-10 minutes)

## Your contact details

We are asking for your contact details so that we can contact you at different stages of this research. We will contact you mainly via email and text messages. Your details will remain confidential and will only be used for this Monash University study.

Full name

__________________________________

Mobile number

__________________________________

Email address

__________________________________

## About you

| | |
|---|---|
| Were you born in Australia? | ◯ Yes<br>◯ No |
| What is the primary language you speak at home? Select one or more of the following options: | ☐ English<br>☐ Mandarin<br>☐ Cantonese<br>☐ Hindi<br>☐ Arabic<br>☐ Other |
| Language spoken at home | ________________________________ |
| Do you currently hold a Health Care or pension card? | ◯ Yes<br>◯ No<br>◯ Don't know |
| What is the postcode of your current residential address? | ________________________________ |

## Sex and relationships

| | |
|---|---|
| What is your current relationship status? | ◯ Single<br>◯ In a relationship but not living with a partner<br>◯ In a relationship and living with a partner<br>◯ Married<br>◯ Separated<br>◯ Divorced<br>◯ Other |
| Relationship status | __________________________________ |
| Do you currently have more than one male sexual partner? | ◯ Yes<br>◯ No |
| Have you ever had an unintended pregnancy?<br><br>By this we mean that you didn't want to be pregnant then or anytime in the future, or you didn't want to be pregnant then but wanted to be pregnant later | ◯ No<br>◯ Yes<br>◯ Don't know |
| How many pregnancies were unintended? | __________________________________ |

**Contraceptive preference The next set of questions are about contraception (birth control). By contraception, we mean anything a person might take, do or use to prevent becoming pregnant. This could also be for pregnancy spacing (which is to increase the gap between children) or to delay a pregnancy.**

**We would like to remind you that there are no right or wrong answers**

Which of the following contraceptive methods have you ever heard of (even if you haven't used the method yourself)?

The oral contraceptive pill (the pill or minipill)
Pills containing the hormones oestrogen and progestogen or progestogen-only pills

◯ Yes - I have heard of it
◯ No - I have not heard of it

| | Know nothing | Know a little | Know a lot | Know everything |
|---|---|---|---|---|
| How much do you feel you know about the oral contraceptive pill and its use? | ◯ | ◯ | ◯ | ◯ |

| | Very unlikely | Unlikely | Likely | Very likely |
|---|---|---|---|---|
| How likely are you to consider using the oral contraceptive pill? | ◯ | ◯ | ◯ | ◯ |

Contraceptive injection (Depo-Provera)
A hormonal injection taken every three months

◯ Yes - I have heard of it
◯ No - I have not heard of it

| | Know nothing | Know a little | Know a lot | Know everything |
|---|---|---|---|---|
| How much do you feel you know about the contraceptive injection and its use? | ◯ | ◯ | ◯ | ◯ |

| | Very unlikely | Unlikely | Likely | Very likely |
|---|---|---|---|---|
| How likely are you to consider using the contraceptive injection? | ◯ | ◯ | ◯ | ◯ |

Contraceptive rod (Implanon)
A match-size rod placed under the skin in your upper arm

◯ Yes - I have heard of it
◯ No - I have not heard of it

| | Know nothing | Know a little | Know a lot | Know everything |
|---|---|---|---|---|
| How much do you feel you know about the contraceptive rod and its use? | ◯ | ◯ | ◯ | ◯ |

| | Very unlikely | Unlikely | Likely | Very likely |
|---|---|---|---|---|
| How likely are you to consider using the contraceptive rod? | ◯ | ◯ | ◯ | ◯ |

Hormonal intrauterine device (also called a hormonal IUD, e.g. Mirena)
A small device that is inserted by a doctor into your uterus that releases hormones

◯ Yes - I have heard of it
◯ No - I have not heard of it

| | Know nothing | Know a little | Know a lot | Know everything |
|---|---|---|---|---|
| How much do you feel you know about the hormonal IUD and its use? | ◯ | ◯ | ◯ | ◯ |

| | Very unlikely | Unlikely | Likely | Very likely |
|---|---|---|---|---|
| How likely are you to consider using the hormonal IUD? | ◯ | ◯ | ◯ | ◯ |

Copper (non-hormonal) IUD
A small device with fine copper wire that is inserted by a doctor into your uterus

◯ Yes - I have heard of it
◯ No - I have not heard of it

| | Know nothing | Know a little | Know a lot | Know everything |
|---|---|---|---|---|
| How much do you feel you know about the copper IUD and its use? | ◯ | ◯ | ◯ | ◯ |

| | Very unlikely | Unlikely | Likely | Very likely |
|---|---|---|---|---|
| How likely are you to consider using the copper IUD? | ◯ | ◯ | ◯ | ◯ |

Contraceptive ring (NuvaRing)

A small plastic ring inserted into the vagina that releases hormones

◯ Yes - I have heard of it
◯ No - I have not heard of it

| | Know nothing | Know a little | Know a lot | Know everything |
|---|---|---|---|---|
| How much do you feel you know about the contraceptive ring and its use? | ◯ | ◯ | ◯ | ◯ |

| | Very unlikely | Unlikely | Likely | Very likely |
|---|---|---|---|---|
| How likely are you to consider using the contraceptive ring? | ◯ | ◯ | ◯ | ◯ |

Male condom

A thin, soft sheath placed over the penis during sex to prevent pregnancy and/or sexually transmitted infections

◯ Yes - I have heard of it
◯ No - I have not heard of it

| | Know nothing | Know a little | Know a lot | Know everything |
|---|---|---|---|---|
| How much do you feel you know about male condoms and their use? | ◯ | ◯ | ◯ | ◯ |

| | Very unlikely | Unlikely | Likely | Very likely |
|---|---|---|---|---|
| How likely are you to consider using male condoms as a contraceptive method? | ◯ | ◯ | ◯ | ◯ |

Withdrawal (pull-out method)
When a man removes his penis from a woman's vagina before he ejaculates (cums)

◯ Yes - I have heard of it
◯ No - I have not heard of it

| | Know nothing | Know a little | Know a lot | Know everything |
|---|---|---|---|---|
| How much do you feel you know about withdrawal? | ◯ | ◯ | ◯ | ◯ |

| | Very unlikely | Unlikely | Likely | Very likely |
|---|---|---|---|---|
| How likely are you to consider using withdrawal? | ◯ | ◯ | ◯ | ◯ |

Emergency contraceptive pill (morning-after pill)

A pill taken after unprotected sex to reduce your chance of pregnancy. This includes if you forget to use your regular contraception or if you think the method has failed

◯ Yes - I have heard of it
◯ No - I have not heard of it

| | Know nothing | Know a little | Know a lot | Know everything |
|---|---|---|---|---|
| How much do you feel you know about the emergency contraceptive pill and its use? | ◯ | ◯ | ◯ | ◯ |

| | Very unlikely | Unlikely | Likely | Very likely |
|---|---|---|---|---|
| How likely are you to consider using the emergency contraceptive pill? | ◯ | ◯ | ◯ | ◯ |

Natural family planning (fertility awareness or rhythm method)

Based on identifying the fertile days of your menstrual cycle and avoiding sex (or using condoms) on those days to prevent pregnancy

◯ Yes - I have heard of it
◯ No - I have not heard of it

| | Know nothing | Know a little | Know a lot | Know everything |
|---|---|---|---|---|
| How much do you feel you know about natural family planning? | ◯ | ◯ | ◯ | ◯ |

| | Very unlikely | Unlikely | Likely | Very likely |
|---|---|---|---|---|
| How likely are you to consider using natural family planning as your contraceptive method? | ◯ | ◯ | ◯ | ◯ |

## Contraceptive preference

| | |
|---|---|
| If you could choose any of these contraceptive methods as your main form of contraception, which one would you choose? | ◯ Oral contraceptive pill (the pill or minipill)<br>◯ Contraceptive injection (Depo-Provera)<br>◯ Contraceptive rod (Implanon)<br>◯ Hormonal intrauterine device (hormonal IUD)<br>◯ Copper intrauterine device (or non-hormonal IUD)<br>◯ Contraceptive ring (NuvaRing)<br>◯ Male Condoms<br>◯ Withdrawal (pull-out method)<br>◯ Emergency contraceptive pill (morning-after pill)<br>◯ Natural family planning (fertility awareness or rhythm method) |

## Contraceptive experience

Which of these contraceptive methods have you ever used?

You can select all of the methods you've used

- ☐ Oral contraceptive pill (the pill or minipill)
- ☐ Contraceptive injection (Depo-Provera)
- ☐ Contraceptive rod (Implanon)
- ☐ Hormonal intrauterine device (hormonal IUD)
- ☐ Copper intrauterine device  (non-hormonal IUD)
- ☐ Contraceptive ring (NuvaRing)
- ☐ Male Condoms
- ☐ Withdrawal (pull-out method)
- ☐ Emergency contraceptive pill (morning-after pill)
- ☐ Natural family planning (fertility awareness or rhythm method)

## Contraceptive experience

| | |
|---|---|
| Do you currently use the oral contraceptive pill? | ◯ Yes<br>◯ No |
| Are you satisfied with using the oral contraceptive pill? | ◯ Yes<br>◯ Somewhat<br>◯ No |
| Do you currently use the contraceptive injection? | ◯ Yes<br>◯ No |
| Are you satisfied with using the contraceptive injection? | ◯ Yes<br>◯ Somewhat<br>◯ No |
| Do you currently use the contraceptive rod? | ◯ Yes<br>◯ No |
| Are you satisfied with using the contraceptive rod? | ◯ Yes<br>◯ Somewhat<br>◯ No |
| Do you currently use the hormonal IUD? | ◯ Yes<br>◯ No |
| Are you satisfied with using the hormonal IUD? | ◯ Yes<br>◯ Somewhat<br>◯ No |
| Do you currently use the copper (non-hormonal) IUD? | ◯ Yes<br>◯ No |
| Are you satisfied with using the copper (non-hormonal) IUD? | ◯ Yes<br>◯ Somewhat<br>◯ No |
| Do you currently use the contraceptive ring? | ◯ Yes<br>◯ No |
| Are you satisfied with using the contraceptive ring? | ◯ Yes<br>◯ Somewhat<br>◯ No |
| In the past 6 months, did you regularly use male condoms as contraception (in other words, you used condoms every time you had sex or almost every time)? | ◯ Yes<br>◯ No<br>◯ Not applicable (didn't have sex) |
| Are you satisfied with using male condoms? | ◯ Yes<br>◯ Somewhat<br>◯ No |
| In the past 6 months, did you regularly use withdrawal as a contraceptive method (in other words, you used withdrawal every time you had sex or almost every time)? | ◯ Yes<br>◯ No<br>◯ Not applicable (didn't have sex) |

| | |
|---|---|
| Are you satisfied with using withdrawal? | ◯ Yes<br>◯ Somewhat<br>◯ No |
| In the past 6 months, did you regularly use the emergency contraceptive pill as a contraceptive method (in other words, you used the emergency contraceptive pill every time you had sex or almost every time)? | ◯ Yes<br>◯ No<br>◯ Not applicable (didn't have sex) |
| Are you satisfied with using the emergency contraceptive pill? | ◯ Yes<br>◯ Somewhat<br>◯ No |
| Do you currently use natural family planning? | ◯ Yes<br>◯ No |
| Are you satisfied with using natural family planning? | ◯ Yes<br>◯ Somewhat<br>◯ No |

## Contraceptive experience

Have any of the following situations ever prevented you from using contraception when you wanted to?

Please select all that you have experienced (You may go to the next page if this question is not relevant to you)

- ☐ Concern about side effects
- ☐ Cost
- ☐ Difficulty finding trustworthy information about contraception
- ☐ Distance between your home and health services
- ☐ Concern that health professionals would judge you
- ☐ Negative healthcare experiences
- ☐ Partner disapproval
- ☐ Partner actively prevented you from using contraception
- ☐ Concern that members of your family or community would disapprove if they found out
- ☐ Using contraception is against your cultural or religious beliefs
- ☐ Other

You may tell us about these other situations here (optional):

__________________________________________

**You have completed survey one Please watch the video below to learn about your contraceptive options**

| | |
|---|---|
| Have you watched the video? | ◯ Yes<br>◯ No |

Please watch the video and confirm above before going to the next page :)

Please go to the next page to start survey two

# Survey two (post-video)

Thank you for watching our educational video about contraception. We now have some further questions we would like you to answer.

Please answer these questions as openly and honestly as you can. There are no right or wrong answers. Some of the questions are very personal but we would like to take this opportunity to remind you that all your responses will remain confidential.

It should take you about 5-10 minutes to complete this survey.

Please submit this page to begin

| | |
|---|---|
| Which of these contraceptive methods is the most effective for preventing pregnancy: | ◯ Condoms<br>◯ Oral contraceptive pill (the pill or minipill)<br>◯ Hormonal intrauterine device (hormonal IUD, e.g. Mirena)<br>◯ Emergency contraceptive pill (morning-after pill) |
| Which of these methods of contraception can make your periods lighter (i.e., reduce bleeding during your menstrual period)?<br><br>You may select one or more answers | ☐ Hormonal intrauterine device (hormonal IUD, e.g. Mirena)<br>☐ Copper (non-hormonal) IUD<br>☐ Contraceptive ring (NuvaRing) |
| Which of these contraceptive methods is effective for more than 3 years without requiring repeated doses?<br><br>You may select one or more answers | ☐ Contraceptive implant (Implanon)<br>☐ Hormonal intrauterine device (hormonal IUD e.g. Mirena)<br>☐ Copper (non-hormonal) IUD<br>☐ Contraceptive ring (NuvaRing)<br>☐ Contraceptive injection (Depo-Provera) |
| Which of these forms of regular contraception can also be used as emergency contraception (i.e., to reduce the likelihood of a pregnancy after unprotected sex)? | ◯ Contraceptive injection (Depo-Provera)<br>◯ Hormonal intrauterine device (Hormonal IUD e.g. Mirena)<br>◯ Progestogen-only pill (POP or minipill)<br>◯ Copper (non-hormonal) IUD |

## Contraceptive preference

Which of the following contraceptive methods have you ever heard of (even if you haven't used the method yourself)?

| | |
|---|---|
| The oral contraceptive pill (the pill or minipill)<br>Pills containing the hormones oestrogen and progestogen or progestogen-only pills | ◯ Yes - I have heard of it<br>◯ No - I have not heard of it |

| | Know nothing | Know a little | Know a lot | Know everything |
|---|---|---|---|---|
| How much do you feel you know about the oral contraceptive pill and its use? | ◯ | ◯ | ◯ | ◯ |

| | Very unlikely | Unlikely | Likely | Very likely |
|---|---|---|---|---|
| How likely are you to consider using the oral contraceptive pill? | ◯ | ◯ | ◯ | ◯ |

| | |
|---|---|
| Contraceptive injection (Depo-Provera)<br>A hormonal injection taken every three months | ◯ Yes - I have heard of it<br>◯ No - I have not heard of it |

| | Know nothing | Know a little | Know a lot | Know everything |
|---|---|---|---|---|
| How much do you feel you know about the contraceptive injection and its use? | ◯ | ◯ | ◯ | ◯ |

| | Very unlikely | Unlikely | Likely | Very likely |
|---|---|---|---|---|
| How likely are you to consider using the contraceptive injection? | ◯ | ◯ | ◯ | ◯ |

| | |
|---|---|
| Contraceptive rod (Implanon)<br>A match-size rod placed under the skin in your upper arm | ◯ Yes - I have heard of it<br>◯ No - I have not heard of it |

| | Know nothing | Know a little | Know a lot | Know everything |
|---|---|---|---|---|
| How much do you feel you know about the contraceptive rod and its use? | ◯ | ◯ | ◯ | ◯ |

| | Very unlikely | Unlikely | Likely | Very likely |
|---|---|---|---|---|
| How likely are you to consider using the contraceptive rod? | ◯ | ◯ | ◯ | ◯ |

| | |
|---|---|
| Hormonal intrauterine device (hormonal IUD, e.g. Mirena)<br>A small device that is inserted by a doctor into your uterus that releases hormones | ◯ Yes - I have heard of it<br>◯ No - I have not heard of it |

| | Know nothing | Know a little | Know a lot | Know everything |
|---|---|---|---|---|
| How much do you feel you know about the hormonal IUD and its use? | ◯ | ◯ | ◯ | ◯ |

| | Very unlikely | Unlikely | Likely | Very likely |
|---|---|---|---|---|
| How likely are you to consider using the hormonal IUD? | ◯ | ◯ | ◯ | ◯ |

Copper (non-hormonal) IUD
A small device with fine copper wire that is inserted by a doctor into your uterus

◯ Yes - I have heard of it
◯ No - I have not heard of it

| | Know nothing | Know a little | Know a lot | Know everything |
|---|---|---|---|---|
| How much do you feel you know about the copper IUD and its use? | ◯ | ◯ | ◯ | ◯ |

| | Very unlikely | Unlikely | Likely | Very likely |
|---|---|---|---|---|
| How likely are you to consider using the copper IUD? | ◯ | ◯ | ◯ | ◯ |

Contraceptive ring (NuvaRing)

A small plastic ring inserted into the vagina that releases hormones

◯ Yes - I have heard of it
◯ No - I have not heard of it

| | Know nothing | Know a little | Know a lot | Know everything |
|---|---|---|---|---|
| How much do you feel you know about the contraceptive ring and its use? | ◯ | ◯ | ◯ | ◯ |

| | Very unlikely | Unlikely | Likely | Very likely |
|---|---|---|---|---|
| How likely are you to consider using the contraceptive ring? | ◯ | ◯ | ◯ | ◯ |

Male condom

A thin, soft sheath placed over the penis during sex to prevent pregnancy and/or sexually transmitted infections

◯ Yes - I have heard of it
◯ No - I have not heard of it

| | Know nothing | Know a little | Know a lot | Know everything |
|---|---|---|---|---|
| How much do you feel you know about male condoms and their use? | ◯ | ◯ | ◯ | ◯ |

| | Very unlikely | Unlikely | Likely | Very likely |
|---|---|---|---|---|
| How likely are you to consider using male condoms as a contraceptive method? | ◯ | ◯ | ◯ | ◯ |

Withdrawal (pull-out method)
When a man removes his penis from a woman's vagina before he ejaculates (cums)

◯ Yes - I have heard of it
◯ No - I have not heard of it

| | Know nothing | Know a little | Know a lot | Know everything |
|---|---|---|---|---|
| How much do you feel you know about withdrawal? | ◯ | ◯ | ◯ | ◯ |

| | Very unlikely | Unlikely | Likely | Very likely |
|---|---|---|---|---|
| How likely are you to consider using withdrawal? | ◯ | ◯ | ◯ | ◯ |

Emergency contraceptive pill (morning-after pill)

A pill taken after unprotected sex to reduce your chance of pregnancy. This includes if you forget to use your regular contraception or if you think the method has failed

◯ Yes - I have heard of it
◯ No - I have not heard of it

| | Know nothing | Know a little | Know a lot | Know everything |
|---|---|---|---|---|
| How much do you feel you know about the emergency contraceptive pill and its use? | ◯ | ◯ | ◯ | ◯ |

| | Very unlikely | Unlikely | Likely | Very likely |
|---|---|---|---|---|
| How likely are you to consider using the emergency contraceptive pill? | ◯ | ◯ | ◯ | ◯ |

Natural family planning (fertility awareness or rhythm method)

Based on identifying the fertile days of your menstrual cycle and avoiding sex (or using condoms) on those days to prevent pregnancy

◯ Yes - I have heard of it
◯ No - I have not heard of it

| | Know nothing | Know a little | Know a lot | Know everything |
|---|---|---|---|---|
| How much do you feel you know about natural family planning? | ◯ | ◯ | ◯ | ◯ |

| | Very unlikely | Unlikely | Likely | Very likely |
|---|---|---|---|---|
| How likely are you to consider using natural family planning as your contraceptive method? | ◯ | ◯ | ◯ | ◯ |

## Contraceptive preference

| | |
|---|---|
| After watching the video, if you could choose any of these contraceptive methods as your main form of contraception, which one would you choose? | ◯ Oral contraceptive pill (the pill or minipill)<br>◯ Contraceptive injection (Depo-Provera)<br>◯ Contraceptive rod (Implanon)<br>◯ Hormonal intrauterine device (hormonal IUD)<br>◯ Copper intrauterine device (or non-hormonal IUD)<br>◯ Contraceptive ring (NuvaRing)<br>◯ Male Condoms<br>◯ Withdrawal (pull-out method)<br>◯ Emergency contraceptive pill (morning-after pill)<br>◯ Natural family planning (fertility awareness or rhythm method) |
| After watching the video, how likely would you be to start using contraception or change from your current contraceptive behaviours? | ◯ Very unlikely<br>◯ Unlikely<br>◯ Likely<br>◯ Very likely |

# 3. Post-intervention survey

Thank you for watching our educational video about contraception.

We now have some further questions we would like you to answer. Please answer these questions as openly and honestly as you can. There are no right or wrong answers to these questions. Some of the questions are very personal but we would like to take this opportunity to remind you that all your responses will remain confidential.

It should take you about ___ to complete this survey.

---

Which two methods are effective for greater than 3 years

- ◯ Implanon
- ◯ Intrauterine device (IUD)
- ◯ NuvaRing
- ◯ Depo-Provera

---

Rank the following methods of contraception from the most effective to the least effective:

Condoms

Most Effective — Moderate Effective — Least Effective

*(Place a mark on the scale above)*

---

IUD (Mirena or Copper IUD)

Most Effective — Moderate Effective — Least Effective

*(Place a mark on the scale above)*

---

Oral contraceptive pill (the pill)

Most Effective — Moderate Effective — Least Effective

*(Place a mark on the scale above)*

**Which of the following birth control or contraceptive methods have you ever heard of prior to this video? (even if you haven't used the method yourself)?**

The combined oral contraceptive pill (the pill)
◯ Yes
◯ No

| | Unlikely | Slightly likely | Very likely | Extremely likely |
|---|---|---|---|---|
| How likely are you likely to consider using any of these contraceptive methods? | ◯ | ◯ | ◯ | ◯ |

The minipill
◯ Yes
◯ No

| | Know nothing | Know a little | Know a lot | Know everything |
|---|---|---|---|---|
| How much do you feel you know about each form of contraception and its use? | ◯ | ◯ | ◯ | ◯ |

| | Unlikely | Slightly likely | Very likely | Extremely likely |
|---|---|---|---|---|
| How likely are you likely to consider using any of these contraceptive methods? | ◯ | ◯ | ◯ | ◯ |

Depo-Provera (3 monthly injection)
◯ Yes
◯ No

| | Know nothing | Know a little | Know a lot | Know everything |
|---|---|---|---|---|
| How much do you feel you know about each form of contraception and its use? | ◯ | ◯ | ◯ | ◯ |

| | Unlikely | Slightly likely | Very likely | Extremely likely |
|---|---|---|---|---|
| How likely are you likely to consider using any of these contraceptive methods? | ◯ | ◯ | ◯ | ◯ |

Implanon (1 match-size rod placed under the skin in your upperarm)
◯ Yes
◯ No

| | Know nothing | Know a little | Know a lot | Know everything |
|---|---|---|---|---|
| How much do you feel you know about each form of contraception and its use? | ◯ | ◯ | ◯ | ◯ |

| | Unlikely | Slightly likely | Very likely | Extremely likely |
|---|---|---|---|---|
| How likely are you likely to consider using any of these contraceptive methods? | ◯ | ◯ | ◯ | ◯ |

Intrauterine device (or 'IUD' - small device put in your uterus by doctor, e.g. Mirena, or Copper T)
◯ Yes
◯ No

| | Know nothing | Know a little | Know a lot | Know everything |
|---|---|---|---|---|
| How much do you feel you know about each form of contraception and its use? | ◯ | ◯ | ◯ | ◯ |

| | Unlikely | Slightly likely | Very likely | Extremely likely |
|---|---|---|---|---|
| How likely are you likely to consider using any of these contraceptive methods? | ◯ | ◯ | ◯ | ◯ |

Contraceptive ring (NuvaRing)
◯ Yes
◯ No

| | Know nothing | Know a little | Know a lot | Know everything |
|---|---|---|---|---|
| How much do you feel you know about each form of contraception and its use? | ◯ | ◯ | ◯ | ◯ |

| | Unlikely | Slightly likely | Very likely | Extremely likely |
|---|---|---|---|---|
| How likely are you to consider using any of these contraceptive methods? | ◯ | ◯ | ◯ | ◯ |

Diaphragm
◯ Yes
◯ No

| | Know nothing | Know a little | Know a lot | Know everything |
|---|---|---|---|---|
| How much do you feel you know about this form of contraception and its use? | ◯ | ◯ | ◯ | ◯ |

| | Unlikely | Slightly likely | Very likely | Extremely likely |
|---|---|---|---|---|
| How likely are you to consider using this contraceptive methods? | ◯ | ◯ | ◯ | ◯ |

Condoms
◯ Yes
◯ No

| | Know nothing | Know a little | Know a lot | Know everything |
|---|---|---|---|---|
| How much do you feel you know about this form of contraception and its use? | ◯ | ◯ | ◯ | ◯ |

| | Unlikely | Slightly likely | Very likely | Extremely likely |
|---|---|---|---|---|
| How likely are you to consider using this contraceptive methods? | ◯ | ◯ | ◯ | ◯ |

Withdrawal
◯ Yes
◯ No

| | Know nothing | Know a little | Know a lot | Know everything |
|---|---|---|---|---|

| | | | | |
|---|---|---|---|---|
| How much do you feel you know about this form of contraception and its use? | ◯ | ◯ | ◯ | ◯ |

| | Unlikely | Slightly likely | Very likely | Extremely likely |
|---|---|---|---|---|
| How likely are you to consider using this contraceptive methods? | ◯ | ◯ | ◯ | ◯ |

Emergency contraception ◯ Yes ◯ No

| | Know nothing | Know a little | Know a lot | Know everything |
|---|---|---|---|---|
| How much do you feel you know about this form of contraception and its use? | ◯ | ◯ | ◯ | ◯ |

| | Unlikely | Slightly likely | Very likely | Extremely likely |
|---|---|---|---|---|
| How likely are you to consider using this contraceptive methods? | ◯ | ◯ | ◯ | ◯ |

Natural family planning (based on identifying the fertile days of your menstrual cycle and avoiding sex in order to avoid a pregnancy). ◯ Yes ◯ No

| | Know nothing | Know a little | Know a lot | Know everything |
|---|---|---|---|---|
| How much do you feel you know about this form of contraception and its use? | ◯ | ◯ | ◯ | ◯ |

| | Unlikely | Slightly likely | Very likely | Extremely likely |
|---|---|---|---|---|
| How likely are you to consider using this contraceptive methods? | ◯ | ◯ | ◯ | ◯ |

## Factors of Contraception

| | |
|---|---|
| After watching the video, if you could choose any contraceptive method which would you choose? | ◯ The combined oral contraceptive pill (the pill)<br>◯ The minipill<br>◯ Depo-Provera (3 monthly injection)<br>◯ Implanon (1 match-size rod placed under the skin in your upperarm)<br>◯ Intrauterine device (or 'IUD' - small device put in your uterus by doctor, e.g. Mirena, or Copper T)<br>◯ Contraceptive ring (NuvaRing)<br>◯ Diaphragm<br>◯ Condoms<br>◯ Withdrawal<br>◯ Emergency contraception<br>◯ Natural family planning (based on identifying the fertile days of your menstrual cycle and avoiding sex in order to avoid a pregnancy). |
| How likely would you be to start/change from your current practice? | ◯ Unlikely<br>◯ Slightly likely<br>◯ Very likely<br>◯ Extremely likely |
| Does family decision still have a significant impact on your choice of contraception? | ◯ Yes<br>◯ No |
| Do you still consider any of the following a barrier to using contraception?<br>Choose one or more of the following options | ◯ Distance to health services<br>◯ Lack of privacy in the community and risk of stigma<br>◯ Lack of privacy from the health provider and risk of stigma<br>◯ Partner disapproval or coercion<br>◯ Parent influence<br>◯ Worries about side effects<br>◯ Cultural reasons |
| How likely are you to consider using a method that does not require daily, weekly or monthly dosing? | ◯ Unlikely<br>◯ Not at all likely<br>◯ Very likely<br>◯ Extremely likely |

## Teaching by Texting to Promote Health Behaviours in Pregnancy

### Eligibility Screen

| **Please answer the following questions:** | **Yes** | **No** |
|---|---|---|
| 1. Are you able to read and understand English at grade 8 level? | ☐ | ☐ |
| 2. Do you live in Canada but outside of British Columbia? | ☐ | ☐ |
| 3. What is your estimated due date? ___________________________ | | |
| 4. Are you expecting one baby? | ☐ | ☐ |
| 5. Are you 15 years of age or older? | ☐ | ☐ |
| 6. Did you have any of the health conditions below prior to pregnancy:<br>High blood pressure requiring medication<br>Heart disease requiring treatment by your doctor<br>Diabetes requiring medication | ☐ | ☐ |
| 7. Do you have a cell phone with cell and internet access? | ☐ | ☐ |
| 8. Have you previously had a baby while participating in SmartMom? | ☐ | ☐ |
| 9. Please provide your cell phone number: __________________ | | |

## Teaching by Texting to Promote Health Behaviours in Pregnancy

### Enrollment Questionnaire

**Part 1 of 6: Your Provincial Health Number**

Please provide your province/territory and your health number:

- ☐ Alberta
- ☐ Manitoba
- ☐ New Brunswick
- ☐ Newfoundland and Labrador
- ☐ Northwest Territories
- ☐ Nova Scotia
- ☐ Nunavut
- ☐ Ontario
- ☐ Prince Edward Island
- ☐ Quebec
- ☐ Saskatchewan
- ☐ Yukon

Health number: ________________________

This is the number used to access health services in your province/territory. It is sometimes called a Personal Health Number (PHN), Health Card Number, Personal Health Identification Number (PHIN), Health Insurance Number, or Health Care Plan Number.

If you provide this number, it will be stored on a secure, fire-wall protected server at BC Children's Hospital in Vancouver, BC. It will not be linked to your study data including your questionnaires. Your PHN will be sent via a secure, encrypted process to your provincial perinatal database and the Canadian Institute for Health Information and will be linked to your pregnancy outcome data. The number is removed from the pregnancy data before the data are returned to the study team. You do not have to provide this number in order to participate in the study, but if you do provide it, it will help us determine if our SmartMom messages help people to have better pregnancy outcomes.

**Part 2 of 6: Learning about You**

We would like to learn more about you. You may answer all or only some of the questions below.

1. How many weeks pregnant are you now?
   _____________ weeks

2. How did you hear about this study?
   - ☐ Nurse (Public Health Nurse, Primary Care Nurse)
   - ☐ Doctor
   - ☐ Midwife

- ☐ Doula
- ☐ Community health program
- ☐ Friend/Family member
- ☐ Facebook
- ☐ Instagram
- ☐ Twitter
- ☐ TikTok
- ☐ Poster
- ☐ Other: _______________

3. Who is your primary caregiver for prenatal care?
   - ☐ Doctor
   - ☐ Midwife
   - ☐ Obstetrician
   - ☐ I haven't gone to prenatal care

4. How old are you?
   _______ Years

5. How many times have you been pregnant?
   ________

6. How many children have you had?
   ________

7. To which ethnic or cultural groups do you identify with? (check all that apply)
   - ☐ Indigenous (e.g. First Nations, Métis, Inuit)
   - ☐ African
   - ☐ East Asian (e.g. Chinese, Vietnamese, Japanese, Korean)
   - ☐ European/Caucasian
   - ☐ Latin/Central/South American
   - ☐ Middle Eastern/Arabic
   - ☐ South Asian (e.g. Indian, Pakistani, Sri Lankan)
   - ☐ Other ____________________

8. If Indigenous, are you
   - ☐ First Nations
   - ☐ Métis
   - ☐ Inuit
   - ☐ Other ____________________

9. Are you status?
   - ☐ Non-Status
   - ☐ Status

10. Do you live on reserve?

- ☐ Yes
- ☐ No

11. Have you moved to Canada from another country?
    - ☐ Yes
    - ☐ No

12. How many years have you been in Canada?
    ________ years

13. What language do you speak at home?
    - ☐ English
    - ☐ Other: _________

14. What is the highest level of education you have completed?
    - ☐ Less than Grade 12
    - ☐ High school (Grade 12) diploma or equivalent
    - ☐ Some technical/trades training (apprenticeship)
    - ☐ Some college or university courses
    - ☐ Full technical or trades training (journeyman)
    - ☐ Bachelor's degree
    - ☐ Master's degree or higher

15. What is your marital status?
    - ☐ Single
    - ☐ Common-law or married
    - ☐ Other: _________

16. What sources of information, other than SmartMom, do you currently use or plan to use for prenatal information? Check all that apply. Add others as needed.
    - ☐ Prenatal education classes
    - ☐ Books
    - ☐ TV/DVDs
    - ☐ Family
    - ☐ Friends
    - ☐ Nurse
    - ☐ Midwife
    - ☐ Doctor
    - ☐ Google searching as needed
    - ☐ website: ___________
    - ☐ Pregnancy apps: _________
    - ☐ Other: ____________

17. Do you have any pre-existing conditions that may affect your pregnancy? (check all that apply)
    - ☐ Diabetes

- ☐ High Blood Pressure
- ☐ Other: ________
- ☐ No pre-existing conditions

18. Would you describe yourself as living in a community where there is at least one hospital with an obstetrician and offering caesarean section if needed?
    - ☐ Yes
    - ☐ No

19. Do you have to travel:
    - ☐ Less than half an hour to see a doctor or midwife?
    - ☐ 30-60 minutes to see a doctor or midwife?
    - ☐ More than an hour to see a doctor or midwife?

20. Please provide the first three digits of your postal code:

    ________

21. How tall were you at the beginning of your pregnancy? Please choose either measure option.
    - ☐ Centimeters: ____________________________________.
    - ☐ Feet/inches: ____________________________________.

22. How much did you weigh at the beginning of your pregnancy? Please choose either option.
    - ☐ Pounds: ___________
    - ☐ Kilograms: _________

23. Please share with us (answers are anonymous) the following:

Currently smoking
- ☐ Yes _____ cigarettes per day
- ☐ No

Currently vaping
- ☐ Yes _____ times per day
- ☐ No

Currently consuming alcohol
- ☐ Yes _____ drinks per week
- ☐ No

Currently using marijuana (cannabis)
- ☐ Yes _____ times cannabis products used per week
- ☐ No

Other recreational or prescription drugs (specify)
- ☐ Yes _____ times used per ___day or per ___ week
- ☐ No

24. What was your gestational age, in weeks, at your first prenatal care visit? ____________.

25. If you have previously had a caesarean section, what are you planning for this pregnancy?
- ☐ Vaginal birth
- ☐ Caesarean section
- ☐ Not sure yet
- ☐ Not applicable (I have not had a previous caesarean section)

26. Some women (such as those over 35 yrs. of age) may be offered genetic screening to check for birth defects or other concerns that are associated with genetics. Are you aware of the options that you have for genetic screening?
- ☐ Yes
- ☐ No

27. Are you aware that you could have a prenatal glucose screening to test for gestational diabetes?
- ☐ Yes
- ☐ No

SmartMom is not intended to replace the advice of your care provider or to give advice about pregnancy complications. Please talk to your care provider about how SmartMom information is relevant to your pregnancy care.

**Thank you for completing our questionnaire. This will help us to understand what kinds of information that women need during pregnancy.**

**Part 3 of 6: Pop Quiz**

**Take our pop quiz and find out what you already know about pregnancy and birth!**

1. Breast milk can protect your baby from getting infections.
   - ☐ True
   - ☐ False
2. Getting enough folic acid (a B vitamin) early in pregnancy prevents some birth defects.
   - ☐ True
   - ☐ False
3. Getting the flu shot while pregnant is unsafe
   - ☐ True
   - ☐ False
4. First labours on average last 10-14 hours once contractions are regular.
   - ☐ True
   - ☐ False
5. Swelling in the hands and face can be a sign of high blood pressure in pregnancy.
   - ☐ True
   - ☐ False
6. The only way to make labour pain manageable is to use an epidural, narcotics such as morphine or fentanyl, or laughing gas (nitrous oxide or Entonox).
   - ☐ True
   - ☐ False
7. Babies should sleep on their backs.
   - ☐ True
   - ☐ False
8. For healthy moms and babies, caesarean section (c-section) is as safe as vaginal birth.
   - ☐ True
   - ☐ False
9. Women should gain approximately 15-25 lbs during pregnancy.
   - ☐ True
   - ☐ False
10. Smoking can cause babies to have low birthweight
    - ☐ True
    - ☐ False

**{Score and commentary}**
**8-10** Thank you for completing the quiz! Congratulations, you are well on your way to supporting a healthy pregnancy.

**6-7** Thank you for completing the quiz! You’re on the right track!

**5 or less** Thank you for completing the quiz!

**Part 4 of 6: How Are You Feeling?**

**This questionnaire, the Edinburgh Postnatal Depression Scale, is commonly used to see how people are coping with the life changes of pregnancy and childbirth. Please select the answer which comes closest to how you have felt in the past 7 days – not just how you feel today.**

1. I have been able to laugh and see the funny side of things
(0) As much as I always could (1) Not quite so much now (2) Definitely not so much now (3) Not at all

2. I have looked forward with enjoyment to things
(0) As much as I ever did (1) Rather less than I used to (2) Definitely less than I used to (3) Hardly at all

3. I have blamed myself unnecessarily when things went wrong
(0) No, never (1) Not very often (2) Yes, some of the time (3) Yes, most of the time

4. I have been anxious or worried for no good reason
(0) No, not at all (1) Hardly ever (2) Yes, sometimes (3) Yes, very often

5. I have felt scared or panicky for no very good reason
(0) No, not at all (1) No, not much (2) Yes, sometimes (3) Yes, quite a lot

6. Things have been getting on top of me
(0) No, I have been coping as well as ever (1) No, most of the time I have coped quite well (2) Yes, sometimes I haven't been coping as well as usual (3) Yes, most of the time I haven't been able to cope

7. I have been so unhappy that I have had difficulty sleeping
(0) No, not at all (1) Not very often (2) Yes, sometimes (3) Yes, most of the time

8. I have felt sad or miserable
(0) No, not at all (1) Not very often (2) Yes, quite often (3) Yes, most of the time

9. I have been so unhappy that I have been crying
(0) No, never (1) Only occasionally (2) Yes, quite often (3) Yes, most of the time

10. The thought of harming myself has occurred to me
(0) Never (1) Hardly ever (2) Sometimes (3) Yes, quite often

**{Score and commentary}**

**8 or less:** Depression is unlikely, you are doing well!

**9-11:** You may be experiencing some symptoms of depression. Check in with yourself in a couple of weeks and consider talking to your maternity care provider or someone you can talk to about your feelings.

**12 or 13:** You have a fairly high possibility of depression. Please be in touch with your maternity care provider and tell them how you are feeling.

**14 and higher:** You are probably experiencing depression. Please make a call to your maternity care provider soon (this week) and tell them how you are feeling.

Here is a list of resources for you to consider in addition to talking to your health care provider:

1) Suicide Crisis Helpline - Call 988 or visit https://988.ca
2) Postpartum Support International – finding a provider in Canada. https://psidirectory.com/canada,canada
3) Anxiety Canada - https://www.anxietycanada.com/
4) Centre for Addiction and Mental Health – Postpartum Depression - https://www.camh.ca/en/health-info/mental-illness-and-addiction-index/postpartum-depression
5) Mental Health Support in Canada - https://www.omama.com/en/newborn/mental-health-supports.asp
6) Mother's Mental Health Toolkit - https://www.iwk.nshealth.ca/MMH

**Part 5 of 6: How Are You Feeling?**

**This Pregnancy-Specific Anxiety Tool is used to see how people are coping with the life changes of pregnancy and childbirth.**

*The following questions are about how often you have experienced each statement during the past week (**last 7 days**). Please choose the option that most closely describes your experience for each statement. Please remember there are no right or wrong answers*

1. I have been able to concentrate on tasks/things that I was doing.
(1) Never (2) Sometimes (3) Most times (4) Always

2. My worries have been constantly on my mind.
(1) Never (2) Sometimes (3) Most times (4) Always

3. I have worried about a lot of things.
(1) Never (2) Sometimes (3) Most times (4) Always

4. There has been so much on my mind that I could not take care of myself properly.
(1) Never (2) Sometimes (3) Most times (4) Always

5. My worries have interfered with my sleep.
(1) Never (2) Sometimes (3) Most times (4) Always

6. When I worried about something, I could not stop thinking about it.
(1) Never (2) Sometimes (3) Most times (4) Always

7. I could not make decisions because I have been worried to think about them.
(1) Never (2) Sometimes (3) Most times (4) Always

8. I have worried so much that it made me cry.
(1) Never (2) Sometimes (3) Most times (4) Always

9. My relationships have been affected negatively because of my worry.
(1) Never (2) Sometimes (3) Most times (4) Always

10. My mind has gone blank because of my worry.
(1) Never (2) Sometimes (3) Most times (4) Always

11. My anxiety has interfered with my daily life.
(1) Never (2) Sometimes (3) Most times (4) Always

12. I have been so worried that I couldn't think about anything else.
(1) Never (2) Sometimes (3) Most times (4) Always

13. I have experienced panic attacks.
(1) Never (2) Sometimes (3) Most times (4) Always

14. I am worried that my baby is being affected by my worry.
(1) Never (2) Sometimes (3) Most times (4) Always

The following questions ask about your feelings about your pregnancy and baby. For each *statement, please indicate your feelings and experiences by choosing one of the response options. Please remember there are no right or wrong answers.*

*[Response Options:1. Not at all; 2. Somewhat; 3. Moderately; 4. Very much]*

15. I did not want to think about my pregnancy because I might lose the baby.
(1) Not at all (2) Somewhat (3) Moderately (4) Very much

16. I have been very afraid of doing something that could harm the baby.
(1) Never (2) Sometimes (3) Most times (4) Always

17. I am concerned (worried) about the health and well-being of my baby.
(1) Never (2) Sometimes (3) Most times (4) Always

18. I am concerned (worried) that my baby could have problems with development.
(1) Never (2) Sometimes (3) Most times (4) Always

19. I am confident that my baby will be healthy.
(1) Never (2) Sometimes (3) Most times (4) Always

20. I am scared about labour.
(1) Never (2) Sometimes (3) Most times (4) Always

21. I am concerned (worried) that the baby could be injured during labour.
(1) Never (2) Sometimes (3) Most times (4) Always

22. I am concerned (worried) that I might have a difficult delivery/labour.
(1) Never (2) Sometimes (3) Most times (4) Always

23. I am afraid that I could die during the pregnancy or labour.
(1) Never (2) Sometimes (3) Most times (4) Always

24. I am worried about getting back into shape after the birth.
(1) Never (2) Sometimes (3) Most times (4) Always

25. I am worried whether I am going to be a good parent.
(1) Never (2) Sometimes (3) Most times (4) Always

26. I am worried that I won't be able to bond with this baby.
(1) Never (2) Sometimes (3) Most times (4) Always

27. I am worried that my partner/support people have to make up for the income I lose during maternity/parental leave.
(1) Never (2) Sometimes (3) Most times (4) Always

28. I am worried if I can afford the baby's expenses.
(1) Never (2) Sometimes (3) Most times (4) Always

29. I am worried how my pregnancy and raising the baby will impact my career/study.
(1) Never (2) Sometimes (3) Most times (4) Always

30. I am worried that my health care provider won't support my decisions about my pregnancy.
(1) Never (2) Sometimes (3) Most times (4) Always

31. I feel my partner/support people is/are available when I need them.
(1) Never (2) Sometimes (3) Most times (4) Always

32. I am worried that I don't have enough support.
(1) Never (2) Sometimes (3) Most times (4) Always

33. I am worried because my relationship with my partner/support people is not going well.
(1) Never (2) Sometimes (3) Most times (4) Always

**{Score and commentary}**
**Score of 10 or less:**. Your scores are within the normal range. If your feelings change or you have concerns, please speak with your maternity care provider.

**Score of 11 or more:** You are probably experiencing pregnancy-specific anxiety, indicating a need for further assessment. You may benefit from access to care and resources. Please call your maternity care provider soon and tell them how you are feeling.

**Part 6 of 6: Your Feelings about Labour and Birth**

**We want to have a sense of how you are feeling about labour and birth at this stage of your pregnancy.**

1. I am worried that labour pain will be too intense.
   (1) strongly disagree, (2) disagree, (3) somewhat disagree, (4) somewhat agree, (5) agree and (6) strongly agree.

2. I feel that I will be able to handle the pain of childbirth.
   (6) strongly disagree, (5) disagree, (4) somewhat disagree, (3) somewhat agree, (2) agree and (1) strongly agree.

3. I trust myself to not panic and know what to do during labour and birth.
   (6) strongly disagree, (5) disagree, (4) somewhat disagree, (3) somewhat agree, (2) agree and (1) strongly agree.

4. I am fearful of birth.
   (1) strongly disagree, (2) disagree, (3) somewhat disagree, (4) somewhat agree, (5) agree and (6) strongly agree.

5. I believe that the baby will be safe throughout labour and birth.
   (6) strongly disagree, (5) disagree, (4) somewhat disagree, (3) somewhat agree, (2) agree and (1) strongly agree.

6. I am afraid that I will be out of control during labour and birth.
   (1) strongly disagree, (2) disagree, (3) somewhat disagree, (4) somewhat agree, (5) agree and (6) strongly agree.

7. Complications may occur during labour and birth but they can be managed.
   (6) strongly disagree, (5) disagree, (4) somewhat disagree, (3) somewhat agree, (2) agree and (1) strongly agree.

8. Birth is unpredictable and risky.
   (1) strongly disagree, (2) disagree, (3) somewhat disagree, (4) somewhat agree, (5) agree and (6) strongly agree.

9. I am afraid of what the labour and birth process will do to my body
   (1) strongly disagree, (2) disagree, (3) somewhat disagree, (4) somewhat agree, (5) agree and (6) strongly agree.

10. I believe that my body will return to a normal healthy state after birth.
    (6) strongly disagree, (5) disagree, (4) somewhat disagree, (3) somewhat agree, (2) agree and (1) strongly agree.

11. I believe that I have adequate support from friends or family to support me through labour and birth. (6) strongly disagree, (5) disagree, (4) somewhat disagree, (3) somewhat agree, (2) agree and (1) strongly agree.

{Adds up and provides score from only first ten questions}

**<42**: It sounds like you've spoken with your caregiver(s) about your expectations and concerns. You might have some natural worries about labour and birth, but you generally feel ready to handle what's up ahead. Keep communicating any concerns you may have as you move forward towards birth-day!

**If you scored 42 – 47**: You have a moderate fear of childbirth. It sounds like you have a few worries about childbirth. It's natural to have some worries about labour and birth, but learning more about it might help you feel less anxiety towards it. Speak to your care provider about your expectations and concerns as you move towards birth-day!

**If your score is 48 or higher**: It sounds like you have a few worries about childbirth. It's natural to feel nervous about labour and birth. Talking about those fears and learning more about the details of delivery may help you feel less anxiety and more able to enjoy these days leading up to labour! Talk to your caregiver or Public Health Unit closest to you about what is realistic to expect when it's time for you to give birth and what your worries are about.

**Thank you for completing our questionnaires. We will send you a $15 gift card to thank you for your time. The gift card will come via text within 2 weeks. You will also be asked to complete a questionnaire at 38 weeks of pregnancy.**

# Teaching by Texting to Promote Health Behaviours in Pregnancy

## 38 Week Questionnaire – Intervention Group

**Part 1 of 5: Your Experience with SmartMom**

1. Have you already had your baby?
   - ☐ Yes, my baby has been born
   - ☐ No, I have not yet had my baby
   - ☐ I experienced a pregnancy loss

2. Throughout your pregnancy, what were your top 3 sources for prenatal information?
   - ☐ SmartMom
   - ☐ Prenatal Education classes
   - ☐ Books
   - ☐ TV/DVDs
   - ☐ Family
   - ☐ Friends
   - ☐ Nurse
   - ☐ Midwife
   - ☐ Doctor
   - ☐ Website(s): ___________
   - ☐ Google searching as needed
   - ☐ Pregnancy app(s): _________
   - ☐ Other: ____________

   How many hours per week in total do you spend on websites, blogs or apps looking at information related to pregnancy and childbirth? _________________ hours

3. How many of your SmartMom messages did you read?
   - ☐ 0-24%
   - ☐ 25-49%
   - ☐ 50-74%
   - ☐ 75-99%
   - ☐ 100%

4. Of the SmartMom message you read, how many did you learn something new from?
   - ☐ None
   - ☐ ~25% of the information is new
   - ☐ ~50% of the information is new
   - ☐ ~75% of the information is new
   - ☐ All of the information is new

5. Which topics were you most likely to read? (select all that apply)

- ☐ How to find prenatal resources online
- ☐ How to find prenatal resources in your community
- ☐ Nutrition
- ☐ Food safety
- ☐ Prenatal screening/testing
- ☐ What to expect at your stage of pregnancy
- ☐ Environmental health and safety
- ☐ Workplace issues
- ☐ Mental health support
- ☐ Physical activity
- ☐ Breastfeeding
- ☐ Oral health
- ☐ Sexual health
- ☐ Encouragement
- ☐ Labour and delivery
- ☐ Other: ___________

6. Of the messages you read, how often did you visit the web links provided?
   - ☐ 0-24%
   - ☐ 25-49%
   - ☐ 50-74%
   - ☐ 75-99%
   - ☐ 100%

7. Have you ever called a phone number included in a SmartMom message?
   - ☐ Yes
   - ☐ No

   {If Yes to 8}

   For which topic? (Select all that apply)
   - ☐ To talk to a dietician, nutritionist, or pharmacist
   - ☐ To talk about safe and healthy relationships
   - ☐ Reducing smoking
   - ☐ Reducing alcohol
   - ☐ Reducing drugs
   - ☐ Other topic_____________________________

8. Did you find the resources you needed when you called the number?
   - ☐ Yes
   - ☐ No

9. Did you bring up topics with your care provider as a result of reading the SmartMom messages?
   - ☐ Yes
   - ☐ No

UBC SmartMom

10. How much do you weigh now, at the end of your pregnancy? Please choose an option.
- ☐ Pounds: ___________.
- ☐ Kilograms: _________.

11. How many visits did you have to your doctor (s) or midwife in total during your pregnancy?
______ visits

12. How many weeks pregnant were you at your first prenatal visit?_____ weeks.

13. We are interested in finding out whether or not SmartMom has helped women adopt healthy behaviours during pregnancy. Please share with us (answers are anonymous) your current behaviour in the following areas:

Currently smoking
- ☐ Yes _____ cigarettes per day
- ☐ No

Currently vaping
- ☐ Yes _____ times per day
- ☐ No

Currently consuming alcohol
- ☐ Yes _____ drinks per week
- ☐ No

Currently using marijuana (cannabis)
- ☐ Yes _____ times cannabis products used per week
- ☐ No

Other recreational or prescription drugs (specify)
- ☐ Yes _____ times used per day or per week
- ☐ No

14. If you have previously had a caesarean section, have you been told by your care provider that vaginal birth is an option for you?
- ☐ Yes, vaginal birth is an option for me
- ☐ No, my care provider has not told me that vaginal birth is an option
- ☐ I have not had a previous caesarean section

15. If you have previously had a caesarean section, what are you planning for this pregnancy?
- ☐ Vaginal birth
- ☐ Caesarean section
- ☐ I have not had a previous caesarean section

16. Can you tell us who was most supportive to you during your pregnancy?

17. Some women (such as those over 35 yrs. of age) may be offered genetic screening to determine if their baby may have any problems associated with genetics. Were you aware that you had a choice to have these tests?

- ☐ Yes
- ☐ No

18. There are currently blood tests available to determine if you were developing diabetes during your pregnancy, called serum glucose testing. Were you aware that you had the choice to have these tests?

- ☐ Yes
- ☐ No

19. Do you find SmartMom messages easy to understand?

- ☐ Yes
- ☐ No
- ☐ Sometimes

If No, or Sometimes, please explain

______________________________

20. How would you rate the overall quality of information of the messages and web links?

- ☐ Excellent
- ☐ Good
- ☐ Satisfactory
- ☐ Not very good
- ☐ Bad

21. Are there other topics we should have covered?

- ☐ Yes
- ☐ No

If yes, please explain

______________________________

22. Would you recommend SmartMom to a friend?

- ☐ Yes
- ☐ No

23. Did you forward any SmartMom messages to someone who was in the study but not receiving SmartMom program messages (i.e. was in the Control arm)?

- ☐ Yes

☐ No

24. What do you see as the advantages of using a texting program compared to an app?

____________________________________

Disadvantages?

____________________________________

25. Do you have suggestions for us to improve SmartMom?

________________________________________

**Thank you!**

**Part 2 of 5: Pop Quiz**

**We would like to find out if SmartMom increases women's knowledge about how to have a healthy pregnancy and birth. To help us do this, please complete the same quiz (below) that you did at the beginning of the pregnancy.**

Breast milk can protect your baby from getting infections.

- ☐ True
- ☐ False

1. Getting enough folic acid (a B vitamin) early in pregnancy prevents some birth defects.
   - ☐ True
   - ☐ False
2. Getting the flu shot while pregnant is unsafe
   - ☐ True
   - ☐ False
3. First labours on average last 10-14 hours once contractions are regular.
   - ☐ True
   - ☐ False
4. Swelling in the hands and face can be a sign of high blood pressure in pregnancy.
   - ☐ True
   - ☐ False
5. The only way to make labour pain manageable is to use an epidural, narcotics such as morphine or fentanyl, or laughing gas (nitrous oxide or Entonox).
   - ☐ True
   - ☐ False
6. Babies should sleep on their backs.
   - ☐ True
   - ☐ False
7. For healthy moms and babies, caesarean section (c-section) is as safe as vaginal birth.
   - ☐ True
   - ☐ False
8. Women should gain approximately 15-25 lbs during pregnancy.
   - ☐ True
   - ☐ False

10. Smoking can cause babies to have low birthweight
    - ☐ True
    - ☐ False

**{Score and commentary}**

**8-10** You know quite a bit about supporting a healthy pregnancy. Congratulations and enjoy your new baby!

**6-7** You're on the right track. Thanks for participating in SmartMom and enjoy your new baby!

**5 or less** Thanks for participating in SmartMom and enjoy your new baby!

**Part 3 of 5: How Are You Feeling?**

**This questionnaire, the Edinburgh Postnatal Depression Scale, is commonly used to see how women are coping with the life changes of pregnancy and childbirth. Please select the answer which comes closest to how you have felt in the past 7 days – not just how you feel today.**

1. I have been able to laugh and see the funny side of things
(0) As much as I always could (1) Not quite so much now (2) Definitely not so much now (3) Not at all

2. I have looked forward with enjoyment to things
(0) As much as I ever did (1) Rather less than I used to (2) Definitely less than I used to (3) Hardly at all

3. I have blamed myself unnecessarily when things went wrong
(0) No, never (1) Not very often (2) Yes, some of the time (3) Yes, most of the time

4. I have been anxious or worried for no good reason
(0) No, not at all (1) Hardly ever (2) Yes, sometimes (3) Yes, very often

5. I have felt scared or panicky for no very good reason
(0) No, not at all (1) No, not much (2) Yes, sometimes (3) Yes, quite a lot

6. Things have been getting on top of me
(0) No, I have been coping as well as ever (1) No, most of the time I have coped quite well (2) Yes, sometimes I haven't been coping as well as usual (3) Yes, most of the time I haven't been able to cope

7. I have been so unhappy that I have had difficulty sleeping
(0) No, not at all (1) Not very often (2) Yes, sometimes (3) Yes, most of the time

8. I have felt sad or miserable
(0) No, not at all (1) Not very often (2) Yes, quite often (3) Yes, most of the time

9. I have been so unhappy that I have been crying
(0) No, never (1) Only occasionally (2) Yes, quite often (3) Yes, most of the time

10. The thought of harming myself has occurred to me
(0) Never (1) Hardly ever (2) Sometimes (3) Yes, quite often

**{Score and commentary}**

**8 or less:** Depression is unlikely, you are doing well!

**9-11:** You may be experiencing some symptoms of depression. Check in with yourself in a couple of weeks and consider talking to your maternity care provider or someone you can talk to about your feelings.

**12 or 13:** You have a fairly high possibility of depression. Please be in touch with your maternity care provider and tell them how you are feeling.

**14 and higher:** You are probably experiencing depression. Please make a call to your maternity care provider soon (this week) and tell them how you are feeling.

Here is a list of resources for you to consider in addition to talking to your health care provider:

1) Talk Suicide Canada - https://talksuicide.ca/
2) Postpartum Support International – finding a provider in Canada. https://psidirectory.com/canada,canada
3) Anxiety Canada - https://www.anxietycanada.com/
4) Centre for Addiction and Mental Health – Postpartum Depression - https://www.camh.ca/en/health-info/mental-illness-and-addiction-index/postpartum-depression
5) Mental Health Support in Canada - https://www.omama.com/en/newborn/mental-health-supports.asp
6) Mother's Mental Health Toolkit - https://www.iwk.nshealth.ca/MMH

**Part 4 of 5: How Are You Feeling?**

**This Pregnancy-Specific Anxiety Tool is used to see how people are coping with the life changes of pregnancy and childbirth.**

*The following questions are about how often you have experienced each statement during the past week (**last 7 days**). Please choose the option that most closely describes your experience for each statement. Please remember there are no right or wrong answers*

1. I have been able to concentrate on tasks/things that I was doing.
(1) Never (2) Sometimes (3) Most times (4) Always

2. My worries have been constantly on my mind.
(1) Never (2) Sometimes (3) Most times (4) Always

3. I have worried about a lot of things.
(1) Never (2) Sometimes (3) Most times (4) Always

4. There has been so much on my mind that I could not take care of myself properly.
(1) Never (2) Sometimes (3) Most times (4) Always

5. My worries have interfered with my sleep.
(1) Never (2) Sometimes (3) Most times (4) Always

6. When I worried about something, I could not stop thinking about it.
(1) Never (2) Sometimes (3) Most times (4) Always

7. I could not make decisions because I have been worried to think about them.
(1) Never (2) Sometimes (3) Most times (4) Always

8. I have worried so much that it made me cry.
(1) Never (2) Sometimes (3) Most times (4) Always

9. My relationships have been affected negatively because of my worry.
(1) Never (2) Sometimes (3) Most times (4) Always

10. My mind has gone blank because of my worry.
(1) Never (2) Sometimes (3) Most times (4) Always

11. My anxiety has interfered with my daily life.
(1) Never (2) Sometimes (3) Most times (4) Always

12. I have been so worried that I couldn't think about anything else.
(1) Never (2) Sometimes (3) Most times (4) Always

13. I have experienced panic attacks.
(1) Never (2) Sometimes (3) Most times (4) Always

14. I am worried that my baby is being affected by my worry.
(1) Never (2) Sometimes (3) Most times (4) Always

The following questions ask about your feelings about your pregnancy and baby. For each *statement, please indicate your feelings and experiences by choosing one of the response options. Please remember there are no right or wrong answers.*

*[Response Options:1. Not at all; 2. Somewhat; 3. Moderately; 4. Very much; *5. Not Applicable]*

15. I did not want to think about my pregnancy because I might lose the baby.
(1) Not at all (2) Somewhat (3) Moderately (4) Very much

16. I have been very afraid of doing something that could harm the baby.
(1) Never (2) Sometimes (3) Most times (4) Always

17. I am concerned (worried) about the health and well-being of my baby.
(1) Never (2) Sometimes (3) Most times (4) Always

18. I am concerned (worried) that my baby could have problems with development.
(1) Never (2) Sometimes (3) Most times (4) Always

19. I am confident that my baby will be healthy.
(1) Never (2) Sometimes (3) Most times (4) Always

20. I am scared about labour.
(1) Never (2) Sometimes (3) Most times (4) Always

21. I am concerned (worried) that the baby could be injured during labour.
(1) Never (2) Sometimes (3) Most times (4) Always

22. I am concerned (worried) that I might have a difficult delivery/labour.
(1) Never (2) Sometimes (3) Most times (4) Always

23. I am afraid that I could die during the pregnancy or labour.
(1) Never (2) Sometimes (3) Most times (4) Always

24. I am worried about getting back into shape after the birth.
(1) Never (2) Sometimes (3) Most times (4) Always

25. I am worried whether I am going to be a good parent.
(1) Never (2) Sometimes (3) Most times (4) Always

26. I am worried that I won't be able to bond with this baby.
(1) Never (2) Sometimes (3) Most times (4) Always

27. * I am worried that my partner has to make up for the income I lose during maternity/parental leave.
(1) Never (2) Sometimes (3) Most times (4) Always (5) Not applicable

28. I am worried if I can afford the baby's expenses.

(1) Never (2) Sometimes (3) Most times (4) Always

29. I am worried how my pregnancy and raising the baby will impact my career/study.
(1) Never (2) Sometimes (3) Most times (4) Always

30. I am worried that my health care provider won't support my decisions about my pregnancy.
(1) Never (2) Sometimes (3) Most times (4) Always

31. * I feel my partner is available when I need him/her.
(1) Never (2) Sometimes (3) Most times (4) Always (5) Not applicable

32. I am worried that I don't have enough support.
(1) Never (2) Sometimes (3) Most times (4) Always

33. * I am worried because my relationship with my partner is not going well.
(1) Never (2) Sometimes (3) Most times (4) Always (5) Not applicable

**{Score and commentary}**
**Score of 10 or less:** Your scores are within the normal range. If your feelings change or you have concerns, speak with your maternity care provider.
**Score of 11 or more:** You are probably experiencing pregnancy-specific anxiety, indicating a need for further assessment. Please call your maternity care provider soon and tell them how you are feeling.

**Part 5 of 5: Your Feelings about Labour and Birth**

**We would also like to know how you feel about labour and birth as your due date approaches. Please take a few moments to tell us how confident you are about labour and birth now.**

1. I am worried that labour pain will be too intense.
   (1) strongly disagree, (2) disagree, (3) somewhat disagree, (4) somewhat agree, (5) agree and (6) strongly agree.

2. I feel that I will be able to handle the pain of childbirth.
   (6) strongly disagree, (5) disagree, (4) somewhat disagree, (3) somewhat agree, (2) agree and (1) strongly agree.

3. I trust myself to not panic and know what to do during labour and birth.
   (6) strongly disagree, (5) disagree, (4) somewhat disagree, (3) somewhat agree, (2) agree and (1) strongly agree.

4. I am fearful of birth.
   (1) strongly disagree, (2) disagree, (3) somewhat disagree, (4) somewhat agree, (5) agree and (6) strongly agree.

5. I believe that the baby will be safe throughout labour and birth.
   (6) strongly disagree, (5) disagree, (4) somewhat disagree, (3) somewhat agree, (2) agree and (1) strongly agree.

6. I am afraid that I will be out of control during labour and birth.
   (1) strongly disagree, (2) disagree, (3) somewhat disagree, (4) somewhat agree, (5) agree and (6) strongly agree.

7. Complications may occur during labour and birth, but they can be managed.
   (6) strongly disagree, (5) disagree, (4) somewhat disagree, (3) somewhat agree, (2) agree and (1) strongly agree.

8. Birth is unpredictable and risky.
   (1) strongly disagree, (2) disagree, (3) somewhat disagree, (4) somewhat agree, (5) agree and (6) strongly agree.

9. I am afraid of what the labour and birth process will do to my body
   (1) strongly disagree, (2) disagree, (3) somewhat disagree, (4) somewhat agree, (5) agree and (6) strongly agree.

10. I believe that my body will return to a normal healthy state after birth.
    (6) strongly disagree, (5) disagree, (4) somewhat disagree, (3) somewhat agree, (2) agree and (1) strongly agree.

11. I believe that I have adequate support from friends or family to support me through labour and birth. (6) strongly disagree, (5) disagree, (4) somewhat disagree, (3) somewhat agree, (2) agree and (1) strongly agree.

{Adds up and provides score from only first ten questions}

**<42**: It sounds like you've spoken with your caregiver(s) about your expectations and concerns. You might have some natural worries about labour and birth, but you generally feel ready to handle what's up ahead. Keep communicating any concerns you may have as you move forward towards birth-day!

**If you scored 42 – 47**: You have a moderate fear of childbirth. It sounds like you have a few worries about childbirth. It's natural to have some worries about labour and birth but learning more about it might help you feel less anxiety towards it. Speak to your care provider about your expectations and concerns as you move towards birth-day!

**If your score is 48 or higher**: It sounds like you have a few worries about childbirth. It's natural to feel nervous about labour and birth. Talking about those fears and learning more about the details of delivery may help you feel less anxiety and more able to enjoy these days leading up to labour! Talk to your caregiver or Public Health Unit closest to you about what is realistic to expect when it's time for you to give birth and what your worries are about.

**Thank you! You will receive an gift card of $20 for completing this questionnaire. You will also be asked to complete a questionnaire at one month after your baby is born.**

# Teaching by Texting to Promote Health Behaviours in Pregnancy

## 38 Week Questionnaire – Control Group

### Part 1 of 5: Your Experience with SmartMom

1. Have you already had your baby?
   - ☐ Yes, my baby has been born
   - ☐ No, I have not yet had my baby
   - ☐ I experienced a pregnancy loss

2. Throughout your pregnancy, what were your top 3 sources for prenatal information?
   - ☐ Prenatal Education classes
   - ☐ Books
   - ☐ TV/DVDs
   - ☐ Family
   - ☐ Friends
   - ☐ Nurse
   - ☐ Midwife
   - ☐ Doctor
   - ☐ Other website: ___________
   - ☐ Google searching as needed
   - ☐ Pregnancy apps: _________
   - ☐ Other: ____________

   How many hours per week in total do you spend on websites, blogs or apps looking at information related to pregnancy and childbirth? _________________ hours

3. How much do you weigh now, at the end of your pregnancy? Please choose an option.
   - ☐ Pounds: ____________.
   - ☐ Kilograms: __________.

4. How many visits did you have to your doctor (s) or midwife in total during your pregnancy?
   ______ visits

5. How many weeks pregnant were you at your first prenatal visit?_____ weeks.

6. We are interested in finding out whether or not texting programs help women adopt healthy behaviours during pregnancy. Please share with us (answers are anonymous) your current behaviour in the following areas:

   Currently smoking
   - ☐ Yes _____ cigarettes per day
   - ☐ No

Currently vaping

☐ Yes _____ times per day
☐ No

Currently consuming alcohol

☐ Yes _____ drinks per week
☐ No

Currently using marijuana (cannabis)

☐ Yes _____ times cannabis products used per week
☐ No

Other recreational or prescription drugs (specify)

☐ Yes _____ times used per _____day or___week
☐ No

7. If you have previously had a caesarean section, have you been told by your care provider that vaginal birth is an option for you?
   ☐ Yes, vaginal birth is an option for me
   ☐ No, my care provider has not told me that vaginal birth is an option
   ☐ I have not had a previous caesarean section

8. If you have previously had a caesarean section, what are you planning for this pregnancy?
   ☐ Vaginal birth
   ☐ Caesarean section
   ☐ I have not had a previous caesarean section

9. Can you tell us who was most supportive to you during your pregnancy?

_____________________________________________

10. Some women (such as those over 35 yrs. of age) may be offered genetic screening to determine if their baby may have any problems associated with genetics. Were you aware that you had a choice to have these tests?

    ☐ Yes
    ☐ No

11. There are currently blood tests available to determine if you were developing diabetes during your pregnancy called serum glucose testing. Were you aware that you had the choice to have these tests?

    ☐ Yes
    ☐ No

12. Did you receive any SmartMom program messages forwarded from someone else (i.e. on the Intervention arm)?

- ☐ Yes
- ☐ No

13. What do you see as the advantages of using a texting program compared to an app?

______________________________________

Disadvantages?

______________________________________

**Thank you!**

**Part 2 of 5: Pop Quiz**

**We would like to find out if SmartMom increases women's knowledge about how to have a healthy pregnancy and birth. To help us do this, please complete the same quiz (below) that you did at the beginning of the pregnancy.**

Breast milk can protect your baby from getting infections.

- ☐ True
- ☐ False

1. Getting enough folic acid (a B vitamin) early in pregnancy prevents some birth defects.
   - ☐ True
   - ☐ False
2. Getting the flu shot while pregnant is unsafe
   - ☐ True
   - ☐ False
3. First labours on average last 10-14 hours once contractions are regular.
   - ☐ True
   - ☐ False
4. Swelling in the hands and face can be a sign of high blood pressure in pregnancy.
   - ☐ True
   - ☐ False
5. The only way to make labour pain manageable is to use an epidural, narcotics such as morphine or fentanyl, or laughing gas (nitrous oxide or Entonox).
   - ☐ True
   - ☐ False
6. Babies should sleep on their backs.
   - ☐ True
   - ☐ False
7. For healthy moms and babies, caesarean section (c-section) is as safe as vaginal birth.
   - ☐ True
   - ☐ False
8. Women should gain approximately 15-25 lbs during pregnancy.
   - ☐ True
   - ☐ False

10. Smoking can cause babies to have low birthweight
    - ☐ True
    - ☐ False

**{Score and commentary}**

**8-10** You know quite a bit about supporting a healthy pregnancy. Congratulations and enjoy your new baby!
**6-7** You're on the right track. Thanks for participating in the SmartMom study and enjoy your new baby!
**5 or less** Thanks for participating in the SmartMom study and enjoy your new baby!

**Part 3 of 5: How Are You Feeling?**

**This questionnaire, the Edinburgh Postnatal Depression Scale, is commonly used to see how women are coping with the life changes of pregnancy and childbirth. Please select the answer which comes closest to how you have felt in the past 7 days – not just how you feel today.**

1. I have been able to laugh and see the funny side of things
(0) As much as I always could (1) Not quite so much now (2) Definitely not so much now (3) Not at all

2. I have looked forward with enjoyment to things
(0) As much as I ever did (1) Rather less than I used to (2) Definitely less than I used to (3) Hardly at all

3. I have blamed myself unnecessarily when things went wrong
(0) No, never (1) Not very often (2) Yes, some of the time (3) Yes, most of the time

4. I have been anxious or worried for no good reason
(0) No, not at all (1) Hardly ever (2) Yes, sometimes (3) Yes, very often

5. I have felt scared or panicky for no very good reason
(0) No, not at all (1) No, not much (2) Yes, sometimes (3) Yes, quite a lot

6. Things have been getting on top of me
(0) No, I have been coping as well as ever (1) No, most of the time I have coped quite well (2) Yes, sometimes I haven't been coping as well as usual (3) Yes, most of the time I haven't been able to cope

7. I have been so unhappy that I have had difficulty sleeping
(0) No, not at all (1) Not very often (2) Yes, sometimes (3) Yes, most of the time

8. I have felt sad or miserable
(0) No, not at all (1) Not very often (2) Yes, quite often (3) Yes, most of the time

9. I have been so unhappy that I have been crying
(0) No, never (1) Only occasionally (2) Yes, quite often (3) Yes, most of the time

10. The thought of harming myself has occurred to me
(0) Never (1) Hardly ever (2) Sometimes (3) Yes, quite often

**{Score and commentary}**

**8 or less:** Depression is unlikely, you are doing well!

**9-11:** You may be experiencing some symptoms of depression. Check in with yourself in a couple of weeks and consider talking to your maternity care provider or someone you can talk to about your feelings.

**12 or 13:** You have a fairly high possibility of depression. Please be in touch with your maternity care provider and tell them how you are feeling.

**14 and higher:** You are probably experiencing depression. Please make a call to your maternity care provider soon (this week) and tell them how you are feeling.

Here is a list of resources for you to consider in addition to talking to your health care provider:

1) Talk Suicide Canada - https://talksuicide.ca/
2) Postpartum Support International – finding a provider in Canada. https://psidirectory.com/canada,canada
3) Anxiety Canada - https://www.anxietycanada.com/
4) Centre for Addiction and Mental Health – Postpartum Depression - https://www.camh.ca/en/health-info/mental-illness-and-addiction-index/postpartum-depression
5) Mental Health Support in Canada - https://www.omama.com/en/newborn/mental-health-supports.asp
6) Mother's Mental Health Toolkit - https://www.iwk.nshealth.ca/MMH

**Part 4 of 5: How Are You Feeling?**

**This Pregnancy-Specific Anxiety Tool is used to see how people are coping with the life changes of pregnancy and childbirth.**

*The following questions are about how often you have experienced each statement during the past week (**last 7 days**). Please choose the option that most closely describes your experience for each statement. Please remember there are no right or wrong answers*

1. I have been able to concentrate on tasks/things that I was doing.
(1) Never (2) Sometimes (3) Most times (4) Always

2. My worries have been constantly on my mind.
(1) Never (2) Sometimes (3) Most times (4) Always

3. I have worried about a lot of things.
(1) Never (2) Sometimes (3) Most times (4) Always

4. There has been so much on my mind that I could not take care of myself properly.
(1) Never (2) Sometimes (3) Most times (4) Always

5. My worries have interfered with my sleep.
(1) Never (2) Sometimes (3) Most times (4) Always

6. When I worried about something, I could not stop thinking about it.
(1) Never (2) Sometimes (3) Most times (4) Always

7. I could not make decisions because I have been worried to think about them.
(1) Never (2) Sometimes (3) Most times (4) Always

8. I have worried so much that it made me cry.
(1) Never (2) Sometimes (3) Most times (4) Always

9. My relationships have been affected negatively because of my worry.
(1) Never (2) Sometimes (3) Most times (4) Always

10. My mind has gone blank because of my worry.
(1) Never (2) Sometimes (3) Most times (4) Always

11. My anxiety has interfered with my daily life.
(1) Never (2) Sometimes (3) Most times (4) Always

12. I have been so worried that I couldn't think about anything else.
(1) Never (2) Sometimes (3) Most times (4) Always

13. I have experienced panic attacks.
(1) Never (2) Sometimes (3) Most times (4) Always

14. I am worried that my baby is being affected by my worry.
(1) Never (2) Sometimes (3) Most times (4) Always

The following questions ask about your feelings about your pregnancy and baby. For each *statement, please indicate your feelings and experiences by choosing one of the response options. Please remember there are no right or wrong answers.*

*[Response Options:1. Not at all; 2. Somewhat; 3. Moderately; 4. Very much]*

15. I did not want to think about my pregnancy because I might lose the baby.
(1) Not at all (2) Somewhat (3) Moderately (4) Very much

16. I have been very afraid of doing something that could harm the baby.
(1) Never (2) Sometimes (3) Most times (4) Always

17. I am concerned (worried) about the health and well-being of my baby.
(1) Never (2) Sometimes (3) Most times (4) Always

18. I am concerned (worried) that my baby could have problems with development.
(1) Never (2) Sometimes (3) Most times (4) Always

19. I am confident that my baby will be healthy.
(1) Never (2) Sometimes (3) Most times (4) Always

20. I am scared about labour.
(1) Never (2) Sometimes (3) Most times (4) Always

21. I am concerned (worried) that the baby could be injured during labour.
(1) Never (2) Sometimes (3) Most times (4) Always

22. I am concerned (worried) that I might have a difficult delivery/labour.
(1) Never (2) Sometimes (3) Most times (4) Always

23. I am afraid that I could die during the pregnancy or labour.
(1) Never (2) Sometimes (3) Most times (4) Always

24. I am worried about getting back into shape after the birth.
(1) Never (2) Sometimes (3) Most times (4) Always

25. I am worried whether I am going to be a good parent.
(1) Never (2) Sometimes (3) Most times (4) Always

26. I am worried that I won't be able to bond with this baby.
(1) Never (2) Sometimes (3) Most times (4) Always

27. I am worried that my partner/support people have to make up for the income I lose during maternity/parental leave.
(1) Never (2) Sometimes (3) Most times (4) Always

28. I am worried if I can afford the baby's expenses.
(1) Never (2) Sometimes (3) Most times (4) Always

29. I am worried how my pregnancy and raising the baby will impact my career/study.
(1) Never (2) Sometimes (3) Most times (4) Always

30. I am worried that my health care provider won't support my decisions about my pregnancy.
(1) Never (2) Sometimes (3) Most times (4) Always

31. I feel my partner/support people is/are available when I need them.
(1) Never (2) Sometimes (3) Most times (4) Always

32. I am worried that I don't have enough support.
(1) Never (2) Sometimes (3) Most times (4) Always

33. I am worried because my relationship with my partner/support people is not going well.
(1) Never (2) Sometimes (3) Most times (4) Always

**{Score and commentary}**
**Score of 10 or less:** Your scores are within the normal range. If your feelings change or you have concerns, speak with your maternity care provider.
**Score of 11 or more:** You are probably experiencing pregnancy-specific anxiety, indicating a need for further assessment. You may benefit from access to care and resources. Please call your maternity care provider soon and tell them how you are feeling.

**Part 5 of 5: Your Feelings about Labour and Birth**

**We would also like to know how you feel about labour and birth as your due date approaches. Please take a few moments to tell us how confident you are about labour and birth now.**

1. I am worried that labour pain will be too intense.
   (1) strongly disagree, (2) disagree, (3) somewhat disagree, (4) somewhat agree, (5) agree and (6) strongly agree.

2. I feel that I will be able to handle the pain of childbirth.
   (6) strongly disagree, (5) disagree, (4) somewhat disagree, (3) somewhat agree, (2) agree and (1) strongly agree.

3. I trust myself to not panic and know what to do during labour and birth.
   (6) strongly disagree, (5) disagree, (4) somewhat disagree, (3) somewhat agree, (2) agree and (1) strongly agree.

4. I am fearful of birth.
   (1) strongly disagree, (2) disagree, (3) somewhat disagree, (4) somewhat agree, (5) agree and (6) strongly agree.

5. I believe that the baby will be safe throughout labour and birth.
   (6) strongly disagree, (5) disagree, (4) somewhat disagree, (3) somewhat agree, (2) agree and (1) strongly agree.

**6.** I am afraid that I will be out of control during labour and birth**.**
   (1) strongly disagree, (2) disagree, (3) somewhat disagree, (4) somewhat agree, (5) agree and (6) strongly agree.

**7.** Complications may occur during labour and birth, but they can be managed**.**
   (6) strongly disagree, (5) disagree, (4) somewhat disagree, (3) somewhat agree, (2) agree and (1) strongly agree.

8. Birth is unpredictable and risky.
   (1) strongly disagree, (2) disagree, (3) somewhat disagree, (4) somewhat agree, (5) agree and (6) strongly agree.

9. I am afraid of what the labour and birth process will do to my body
   (1) strongly disagree, (2) disagree, (3) somewhat disagree, (4) somewhat agree, (5) agree and (6) strongly agree.

10. I believe that my body will return to a normal healthy state after birth.
    (6) strongly disagree, (5) disagree, (4) somewhat disagree, (3) somewhat agree, (2) agree and (1) strongly agree.

11. I believe that I have adequate support from friends or family to support me through labour and birth. (6) strongly disagree, (5) disagree, (4) somewhat disagree, (3) somewhat agree, (2) agree and (1) strongly agree.

{Adds up and provides score from only first ten questions}

**<42**: It sounds like you've spoken with your caregiver(s) about your expectations and concerns. You might have some natural worries about labour and birth, but you generally feel ready to handle what's up ahead. Keep communicating any concerns you may have as you move forward towards birth-day!

**If you scored 42 – 47**: You have a moderate fear of childbirth. It sounds like you have a few worries about childbirth. It's natural to have some worries about labour and birth but learning more about it might help you feel less anxiety towards it. Speak to your care provider about your expectations and concerns as you move towards birth-day!

**If your score is 48 or higher**: It sounds like you have a few worries about childbirth. It's natural to feel nervous about labour and birth. Talking about those fears and learning more about the details of delivery may help you feel less anxiety and more able to enjoy these days leading up to labour! Talk to your caregiver or Public Health Unit closest to you about what is realistic to expect when it's time for you to give birth and what your worries are about.

**Thank you! You will receive an gift card of $20 for completing this questionnaire. You will also be asked to complete a questionnaire at one month after your baby is born.**

## Teaching by Texting to Promote Health Behaviours in Pregnancy

### One Month Postnatal Questionnaire

1. When was your baby born? ___________________________

2. How much did you weigh at the end of your pregnancy? (Choose either option)
   a. Pounds: ____________.
   b. Kilograms: __________.

3. How many visits did you have to your doctor (s) or midwife in total during your pregnancy?
   ______ visits

4. Did you have any complications during your pregnancy? (check all that apply)
   - ☐ Diabetes
   - ☐ High Blood Pressure
   - ☐ Depression
   - ☐ Placenta previa
   - ☐ RH iso-immunization
   - ☐ Preterm labour
   - ☐ Hyperemesis gravidarium
   - ☐ Other ___________________
   - ☐ None

5. What was your employment status during your pregnancy? (tick one only)?
   - ☐ Employed full time
   - ☐ Employed part time
   - ☐ Not employed (SKIP to question 10)
   - ☐ Other (please specify) _____________

6. On average, how many days did you work per week during pregnancy?

   ______ days per week

7. On average, how many hours did you work per day during pregnancy?
   ______ hours per day

Did you miss work during your pregnancy due to concerns related to your pregnancy?
- ☐ No, I did not miss work
- ☐ Yes, I missed work due to: (check all that apply)
  - ☐ Fatigue
  - ☐ Nausea and/or vomiting
  - ☐ Complications of pregnancy _______________

If no for above, SKIP TO QUESTION 8

8. During your pregnancy, how many work days in total were you absent from work because of health concerns related to your pregnancy?
- ☐ 0-3 days
- ☐ 4-7 days
- ☐ 1-2 weeks
- ☐ 3-4 weeks
- ☐ 4+ weeks

9. Did you begin your maternity leave earlier than planned:
- ☐ Yes
- ☐ No

10. When did you begin maternity leave?
_____ weeks of pregnancy

11. If you have previously had a caesarean section, how did you deliver your baby this time?
    a. Vaginal birth
    b. Caesarean section
    c. I did not have a previous caesarean section

12. Did you develop gestational diabetes during your pregnancy?
- ☐ Yes
- ☐ No

13. A preterm birth is when a baby is born before 37 weeks of pregnancy. Did you have a preterm birth?
- ☐ Yes
- ☐ No

14. Were you told by your care provider that your baby was small for gestational age at birth?
- ☐ Yes
- ☐ No

15. While in hospital or for the first couple of days after a home delivery, did you exclusively breastfeed your baby (i.e.: no formula)?
- ☐ Yes
- ☐ No

**Thank you! This concludes our SmartMom questionnaires. Your answers will help us continue to improve our program so that we can give pregnant people the information they need to make decisions about their health during their pregnancy and birth.**
